# SRSF3 plays a key role in liver cancer sex disparity by facilitating hepatocyte expression of ERα and FOXA

**DOI:** 10.1073/pnas.2536317123

**Published:** 2026-09-29

**Authors:** Lulu Yu, Masahiko Ajiro, Vladimir Majerciak, Alexei Lobanov, Yafeng He, Maggie Cam, Baktiar Karim, Deanna Gotte, Chuxia Deng, Keji Zhao, Douglas R. Lowy, Nicholas J. G. Webster, Zhi-Ming Zheng

**Affiliations:** ^a^Tumor Virus RNA Biology Section, HIV Dynamics and Replication Program, Center for Cancer Research, National Cancer Institute, NIH, Frederick, MD 21702; ^b^Center for Cancer Research Collaborative Bioinformatics Resource, National Cancer Institute, NIH, Bethesda, MD 20892; ^c^ Laboratory of Epigenome Biology, Systems Biology Center, National Heart, Lung, and Blood Institute, NIH, Bethesda, MD 20892; ^d^ Frederick National Laboratory for Cancer Research, Frederick, MD 21702; ^e^ Faculty of Synthetic Biology, Shenzhen University of Advanced Technology, Shenzhen 518107, China; ^f^Laboratory of Cellular Oncology, Center for Cancer Research, National Cancer Institute, NIH, Bethesda, MD 20892; ^g^ Department of Medicine, University of California San Diego, La Jolla, CA 92093

**Keywords:** SRSF3, RNA splicing, liver cancer, breast cancer, gender disparity

## Abstract

SRSF3 (SRp20) is an essential RNA splicing factor. By conditional knockout (KO) of Srsf3, we found Srsf3 at its physiological level plays a tumor-suppressive role in mouse liver carcinogenesis. Srsf3 KO in hepatocytes disrupted sex disparity in the development of DEN-induced liver cancer and resulted in high incidence of liver cancer in both male and female mice. In contrast, Srsf3 is oncogenic in the mouse mammary glands and Srsf3 KO significantly delayed the development of Erbb2 breast cancer. Srsf3 KO significantly increased the expression of transcription factors Sox4, E2f1, and Myc, calcium channel factor Trpv4, and zinc-binding Trim6 in liver cancer, but did not in Erbb2 breast cancer. Srsf3 KO enhanced the expression of glucose transmembrane transporter Mfsd4a and translation initiator Eif4a2 in DEN-induced liver cancer but reduced Mfsd4a and Eif4a2 expression in breast cancer. More importantly, Srsf3 KO reduced the expression of hepatocyte ERα and transcription factor Foxa family genes essential for sexual dimorphism in liver cancer and their downstream targets leukemia inhibitory factor receptor Lifr and epidermal growth factor receptor Egfr, but increased the expression of Myc. We further evidenced that SRSF3 binds to FOXA1 promoter and is responsible for FOXA1 promoter activity and FOXA1 expression in liver cancer cells. Together, our data highlight a functional paradigm of SRSF3 at its physiological level in tissue context-dependent gene regulation and in facilitating hepatocyte ERα and FOXA1 expression for sex disparity in liver cancer development.

## Significance

Conditional Srsf3 KO from mouse liver hepatocytes or mammary glands revealed that Srsf3 is tumor-suppressive in development of DEN-induced liver cancer but oncogenic in Erbb2-mammary tumorigenesis by differential regulation of different set of genes in two different organs. Despite ERα and FOXA family are involved in the sex disparity in development of liver cancer, what controls the expression of ERα and FOXA family genes was a mystery. We found that SRSF3, in addition to regulate RNA splicing for expression of a subset genes, is a key regulator for sex disparity in the development of liver cancer and is required, presumably through transcriptional regulation, for the expression of ERα and FOXA family genes and their downstream targets LIFR, EGFR, and Myc.

Serine/arginine-rich splicing factor 3 (SRSF3), also called SRp20, is the smallest member of serine/arginine-rich proteins (SR proteins) (1–4). SRSF3 promotes viral and host RNA splicing when binding to an exonic cis-element (5–9), but inhibits RNA splicing when binding to an intronic element (10). Besides mRNA splicing, SRSF3 acts as a cytoplasm-nuclear shuttling protein and plays a critical role in many cellular processes, including RNA polyadenylation (11, 12), RNA export (13, 14), miRNA biogenesis (7, 15, 16), and protein translation (17, 18). SRSF3 was found indispensable for embryo development (19). Moreover, we recently discovered that SRSF3 is an antiapoptotic and antisenescence factor essential for cell proliferation by promoting the G2/M transition and is oncogenic as a proto-oncogene when overexpressed (20, 21) by regulating genome-wide expression of numerous genes at transcriptional and posttranscriptional levels (7, 22). SRSF3 also prevents a cryptic intron in SRSF1 exon 4 from RNA splicing to escape nonsense-mediated decay (NMD) of the spliced SRSF1 mRNA (7) and to express SRSF1 as a proto-oncoprotein (23–26). Cancer is a chronic and polygenic disease involving a complex, multistep process and multiple risk factors (27–29). A variety of human cancer tissues exhibit highly increased expression of SRSF3 (4, 20, 30, 31).

Liver cancer is the sixth most diagnosed cancer and ranks third in cancer mortality worldwide, with liver cancer incidence and death in men significantly higher than in women (32). Estrogen and estrogen receptor α (ERα) are contributing factors for female resistance to hepatocellular carcinoma (HCC) (33, 34). Foxa1 and Foxa2 are also essential for sexual dimorphism in liver cancer and in cooperating ERα, suppress Myc expression (35). SRSF3 expression is high in most liver cancer cases (20) and is essential for the differentiation of hepatocytes and their metabolic function (36). SRSF3 reduction contributes to progressive liver disease (37), and mice with Srsf3 knock-out (KO) developed liver cancer with aging (38), suggesting a possible tumor suppressive effect of SRSF3. However, the molecular mechanisms of how Srsf3 functions at a physiological level and in the development of liver cancer remain to be determined.

Breast cancer is the most frequently diagnosed cancer and a leading cause of cancer death in women worldwide (32), with over 70% of breast cancers harboring the activation of the nuclear hormone receptor ERα (39). A recent study shows that ERα RNA-binding activity contributes to breast cancer progression (40). Breast cancer tissues also show increased SRSF3 expression (20), and the increased expression of SRSF3 was found to correlate with breast cancer progression and 5-y overall survival (20). SRSF3 was found to be essential for triple-negative breast cancer progression by interacting with TDP43 and regulation of alternative RNA splicing of CD44 variants (41, 42). Nevertheless, the function of SRSF3 in other types of breast cancer remains to be explored.

Human SRSF3 gene is in size of 8,422 bp on chromosome 6 and mouse Srsf3 gene is in size of 9,423 bp on chromosome 17 (43). Both contain seven exons and six introns and encode 164-aa SRSF3 protein with 100% amino acid identity between the mouse and human homologues. Their transcription start sites and polyadenylation cleavage sites were recently remapped and updated (43). The major SRSF3 mRNA isoform with exclusion of exon 4 (a poison exon), which encodes the full-length protein, is 1,411-nt long generated by alternative RNA splicing and alternative polyadenylation (43). Similarly, major mouse Srsf3 mRNA isoform is only 1,295-nt long with a shorter 3′ UTR over the human SRSF3 3′ UTR. The sizes of both human and mouse SRSF3 mRNAs are much shorter than the NCBI annotated SRSF3 transcripts (43). By using Cre-LoxP technology and albumin (Alb)-promoter-driving Cre expression in liver tissues and mouse mammary tumor virus (MMTV) promoter-driving Cre expression in mammary tissues, we conditionally knocked out Srsf3 expression separately from liver and mouse mammary glands. Srsf3 KO on liver carcinogenesis was evaluated by intraperitoneal injection of 15-d-old mice with diethylnitrosamine (DEN), a chemical liver carcinogen (34). Srsf3 KO on breast carcinogenesis was evaluated by further crossing the Srsf3 KO mouse with the mouse carrying a mutant c-neu (Erbb2 or HER2) (44). We demonstrated that Srsf3 at its physiological level is tumor-suppressive in liver cancer development but oncogenic in breast cancer formation by regulation of expression of a distinguished set of genes. Importantly, we found that SRSF3 is a key player facilitating the expression of ERα and FOXA family genes which contribute to sex disparity in the development of liver cancer.

## Results

### Srsf3 Conditional KO in Mouse Liver Tissues and Mammary Glands.

SRSF3 is overexpressed in a variety of human cancers (4, 20, 30, 31), including liver cancer and breast cancer (*SI Appendix*, Fig. S1). To further understand how SRSF3 functions in regulation of expression of host genes which ensure cell proliferation and cell cycle progression (20, 21), we investigated roles of SRSF3 at its physiological level in tissues in development of liver and breast cancer. Conditional liver-specific Srsf3 KO by Alb-Cre expression and Srsf3 KO in mouse mammary glands by MMTV-Cre expression were based on Srsf3-floxed mice (19) (*SI Appendix*, Fig. S2 *A* and *B*). We confirmed the specific homozygous Srsf3 KO in liver tissues and mouse mammary gland tissues first by RNA-seq (*SI Appendix*, Fig. S2 *C* and *D*) and subsequently by RT-PCR (*SI Appendix*, Fig. S2 *E* and *F*), showing the KO of Srsf3 exon 2 and exon 3. Because the poison exon 4 is normally skipped in the WT mRNA (45, 46), the detected Srsf3 mRNA having the Cre-mediated KO of exon 2 and exon 3 in liver and breast tissues appeared as the spliced product from the exon 1 to exon 5 (*SI Appendix*, Fig. S2 *E* and *F*). The residual WT Srsf3 expression in the Srsf3 KO liver or mammary gland tissues (*SI Appendix*, Fig. S2 *C–F*) was also detected from infiltrated leukocytes or other types of cells in the tissues lacking Albumin or MMTV promoter activities.

We noticed that mice with conditional Srsf3 KO in livers and mammary glands did not show embryonic lethality. However, mice with liver conditional Srsf3 KO were born in a smaller size, less active, and have less appetite. The most (~90%) died by ~14 d of birth (36) (*SI Appendix*, Fig. S3*A*). After chemical carcinogen diethylnitrosamine (DEN) intraperitoneal (i.p.) injection of survived mice at day 15 of age (*SI Appendix*, Fig. S3*B*), the death incidence of liver homozygous Srsf3 KO mice reduced to ~24% in a month after birth (*SI Appendix*, Fig. S3*C*). However, the survived male and female mice from the death crisis recovered their body weight and size comparable to the Srsf3 WT mice by 2 mo of age, consistent with previous study (38). Such growth failure or early death phenotype did not happen to the mice with mammary Srsf3 KO, nor to the mice bearing only heterozygous Srsf3 KO (hetKO) in the liver tissues (*SI Appendix*, Fig. S3 *B* and *C*). Data suggest that conditional Srsf3 KO in liver had transient impacts on liver function responsible for the newborn growth, which subsequently could be overcome at the adulthood age. Liver function tests indicated that mice livers bearing Srsf3 KO displayed more damage to their liver function, with significant increase of ALT, ALP, and amylase activities, whereas urea nitrogen and total bilirubin were normal (*SI Appendix*, Fig. S4).

### Srsf3 Is Tumor-Suppressive in DEN-Induced Liver Carcinogenesis but Oncogenic in the Development of Erbb2 Breast Cancer.

We then investigated the tissue-specific KO of Srsf3 on cancer induction in two well-established mouse cancer models (*SI Appendix*, Fig. S3*B*): DEN-induced liver cancer (34) and Erbb2-induced breast cancer (44) (Erbb2 breast cancer in this report). The mice with conditional KO of Srsf3 in the liver hepatocytes with intraperitoneal (i.p.) DEN injection (25 mg/kg at 15 d of age) and in the mammary glands bearing the mutant rat c-neu (V664E) replacement of mouse Erbb2 were examined weekly by palpation for liver tumor development from 6 mo of age and for breast tumor development from a month of age, up to 18 mo (a total of 78 wk). Additionally, a subgroup of mice was killed humanely in the sixth month for tissue pathological examination. The Kaplan-Meier curves for animals with palpable tumors were plotted up to 18 mo.

**Fig. 1.**
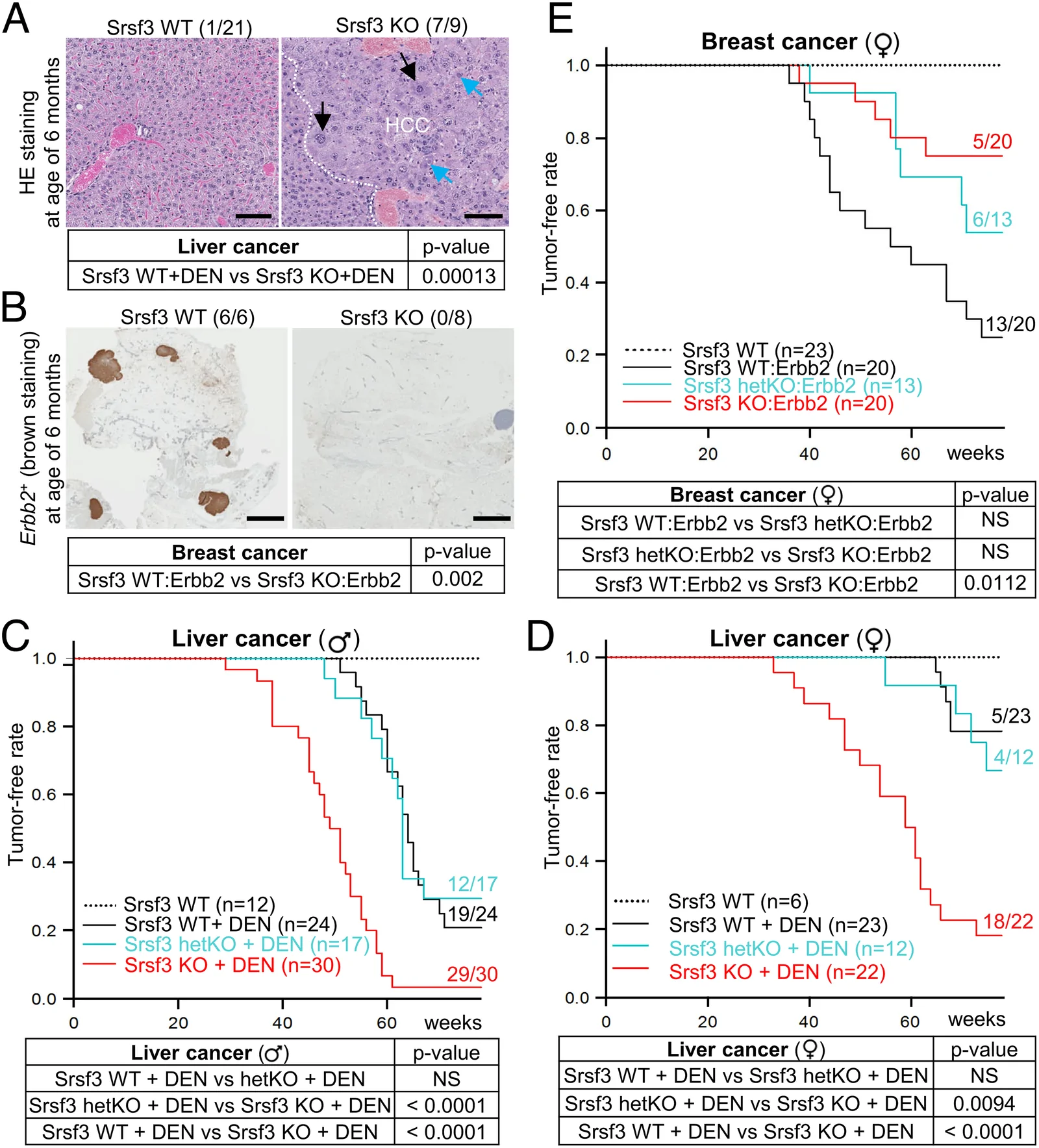
Srsf3-KO promotes and disrupts sex disparity in DEN-induced liver cancer, but delays development of Erbb2 breast cancer. (*A*) H&E staining of Srsf3 WT and Srsf3 KO liver tissues at age of 6 mo. Seven of nine Srsf3 KO mice developed HCC, but only one of 21 Srsf3 WT mice developed HCC. Black arrow, karyomegaly. Blue arrow, intranuclear cytoplasmic invagination. (Scale bar, 100 µm). (*B*) Erbb2 (c-neu) staining of Srsf3 WT and Srsf3 KO mammary tissues at 6 mo of age. Six of six Srsf3 WT mice examined developed Erbb2-tumors, but none of all eight Srsf3 KO mice had Erbb2 tumors (Scale bar, 2 mm). The *P*-values in (*A*) and (*B*) were calculated by Fisher’s exact test. (*C* and *D*) Kaplan-Meier curves for the observed tumor formation rates were plotted up to 18 mo of mouse age for DEN-induced liver cancer in male (*C*) and female (*D*) mice with or without liver Srsf3 hetKO or homozygous Srsf3 KO. (*E*) Kaplan-Meier curves for the observed tumor formation rates were plotted till 18 mo of mouse age for Erbb2 breast cancer in female mice with or without mammary Srsf3 hetKO or homozygous Srsf3 KO. The *P*-values (*C*–*E*) were determined by the log-rank test between indicated groups.

We found that Srsf3 plays opposite roles in the development of liver and breast cancers. In DEN-induced liver cancer model, Srsf3 was tumor suppressive. We found seven out of nine mice (78%) with liver Srsf3 KO in the sixth month of animal age, but only one of 21 mice (4.8%) bearing WT Srsf3 with DEN injection, developed pathological HCC (Fig. 1*A*). In contrast, Srsf3 in the Erbb2 breast tumor model was oncogenic in promoting breast tumor formation because all mammary glands with WT Srsf3 expression in six Erbb2 mice exhibited c-neu (Erbb2)-positive tumor foci, but none of eight Erbb2 mice with the Srsf3 KO had any detectable tumor foci in the mammary glands at the sixth month of mice age (Fig. 1*B*).

Srsf3’s tumor suppressive role in liver became more obvious during 18 mo of observation, of which cancer was further confirmed by histopathological examination of killed humanely mouse livers. Surprisingly, Srsf3 was found to play an important sex-disparity role in the development of DEN-induced liver cancer, which male mice are prone to develop DEN-induce liver cancer more often than female mice (34). Because of female sex hormone, female mice in general are more resistant than male mice to liver cancer induction (34) by DEN (*P* < 0.01) (*SI Appendix*, Fig. S5*A*). We found Srsf3 KO diminished this sex disparity in the liver cancer development induced by DEN (compare Fig. 1*C* with Fig. 1*D*, also *SI Appendix*, Fig. S5*B*). Both male (Fig. 1*C*) and female (Fig. 1*D*) mice with Srsf3 KO show a much higher incidence in DEN-induced liver cancer (*P* < 0.0001) when compared to WT Srsf3 mice, with no difference in incidence of palpable liver tumor up to 50 wk of DEN injection (*SI Appendix*, Fig. S5*B*). The female mice with Srsf3 KO exhibited substantially increased tumor burden than WT mice by pathological measurement (*SI Appendix*, Fig. S5*C*). The mice with hetSrsf3 KO showed no difference in DEN-induction of liver cancer from WT Srsf3 mice (Fig. 1 *C* and *D*), suggesting a single copy of Srsf3 allele expression (hetSrsf3 KO) is sufficient to delay liver cancer formation.

In contrast, Srsf3’s oncogenic role in the development of mouse breast tumors by 18 mo was striking. A significant delay (*P* = 0.0112) in tumor development in Erbb2 mice with Srsf3 KO was observed when compared to Erbb2 mice with WT Srsf3 (Fig. 1*E*), whereas the Erbb2 mice with hetSrsf3 KO showed intermediate outcome (no statistical significance) in overall tumor formation. Mice without Erbb2 exhibited no breast tumor during 18 mo of observation period (Fig. 1*E*).

### Srsf3 Regulates the Expression of a Different Set of the Genes Associated with DEN-Induced Liver Cancer Distinguishable from Erbb2 Breast Cancer.

To understand how Srsf3 KO in liver hepatocytes and mammary glands could have an opposite effect on carcinogenesis, which might associate with altered gene expression and RNA splicing in two different organs, we conducted RNA-seq analyses for DEN-induced Srsf3 KO and Srsf3 WT liver cancer tissues (*SI Appendix*, Fig. S6 *A* and *B* and Dataset S1), and Erbb2-induced Srsf3 KO and Srsf3 WT breast cancer tissues (*SI Appendix*, Fig. S6 *D* and *E* and Dataset S2). We found that the gene expression profile from DEN-induced Srsf3 KO liver cancer was very different from Srsf3 KO Erbb2 breast cancer. In addition to tissue-specific gene expression, this difference could be also interpreted as different carcinogens used in our studies, with DEN as a chemical carcinogen in liver cancer induction and Erbb2 as an oncogene in breast cancer induction.

In DEN-induced liver cancer tissues, we found 927 genes significantly upregulated, and 1,017 genes significantly downregulated in Srsf3 KO vs. Srsf3 WT liver cancers (*SI Appendix*, Fig. S6*B* and Dataset S1). The genes upregulated in Srsf3 KO liver cancer tissues were involved in cancer-related oxidative phosphorylation (downregulated in Srsf3 KO breast cancer), epithelial–mesenchymal transition, fatty acid metabolism, and E2F targets based on the GSEA hallmark gene-sets pathway analysis, whereas those downregulated genes in Srsf3 KO liver cancer were the genes responsible for interferon responses (also in Srsf3 KO breast cancer), coagulation, and KRAS signaling (*SI Appendix*, Fig. S6*C*). Subsequently, we identified and verified by NanoString analysis of 43 genes with altered expression by RNA-seq in Srsf3 KO liver cancer tissues including the top listed genes with upregulated or down-regulated expression (*SI Appendix*, Fig. S7*A* and Dataset S3) and the genes not on the RNA-seq top list but with important functions (*SI Appendix*, Fig. S7*B*, in black, and Dataset S3), of which Nol3, Egfr, and Ahnak2 are involved in RNA processing, Cxcl11, Zbp1, and Fgl2 participate the regulated interferon pathway, and Slc12a2 and Errfi1 are Srsf3-regulated genes identified in our previous publication (7). Interestingly at sixth month of animal age, 11 out of the 43 validated genes already showed the up- or down-regulated expression in Srsf3 KO liver cancer (*SI Appendix*, Fig. S8*A* and Dataset S3), suggesting that these genes could remain the altered expression up to 18 mo (terminal time).

In the Erbb2 breast cancer, a total of 425 differentially expressed genes were identified, of which 159 genes were upregulated, and 266 genes were downregulated in Srsf3 KO breast cancer when compared to WT Srsf3 breast cancer (*SI Appendix*, Fig. S6*E* and Dataset S2). The top enriched gene sets in upregulated pathways were involved in cancer-related hypoxia, mitotic spindle, epithelial–mesenchymal transition, and p53 pathways, but the top enriched gene sets in the downregulated pathways were involved in interferon responses and oxidative phosphorylation (*SI Appendix*, Fig. S6*F*). The heatmap showed the expression of RNA-seq identified top 25 genes either abundantly (RPKM ≥ 2) upregulated or downregulated (*SI Appendix*, Fig. S7*C*). By NanoString technology, we verified the differential expression of top 40 genes identified by RNA-seq (*SI Appendix*, Fig. S7*D* and Dataset S3). Among them, upregulated Pcbp4, Ahnak2, and Ctif are the genes involved in RNA processing and translation initiation, and downregulated Stat2, IL18bp, and Casp7 are the genes involved in interferon responsive pathway. Interestingly, 20 out of the 40 NanoString-validated genes at the sixth month of animal age (*SI Appendix*, Fig. S8*B* and Dataset S3) remained persistently being upregulated or downregulated in Srsf3 KO breast cancer tissues for 18 mo (terminal time) when compared to Srsf3 WT breast cancer tissues.

**Fig. 2.**
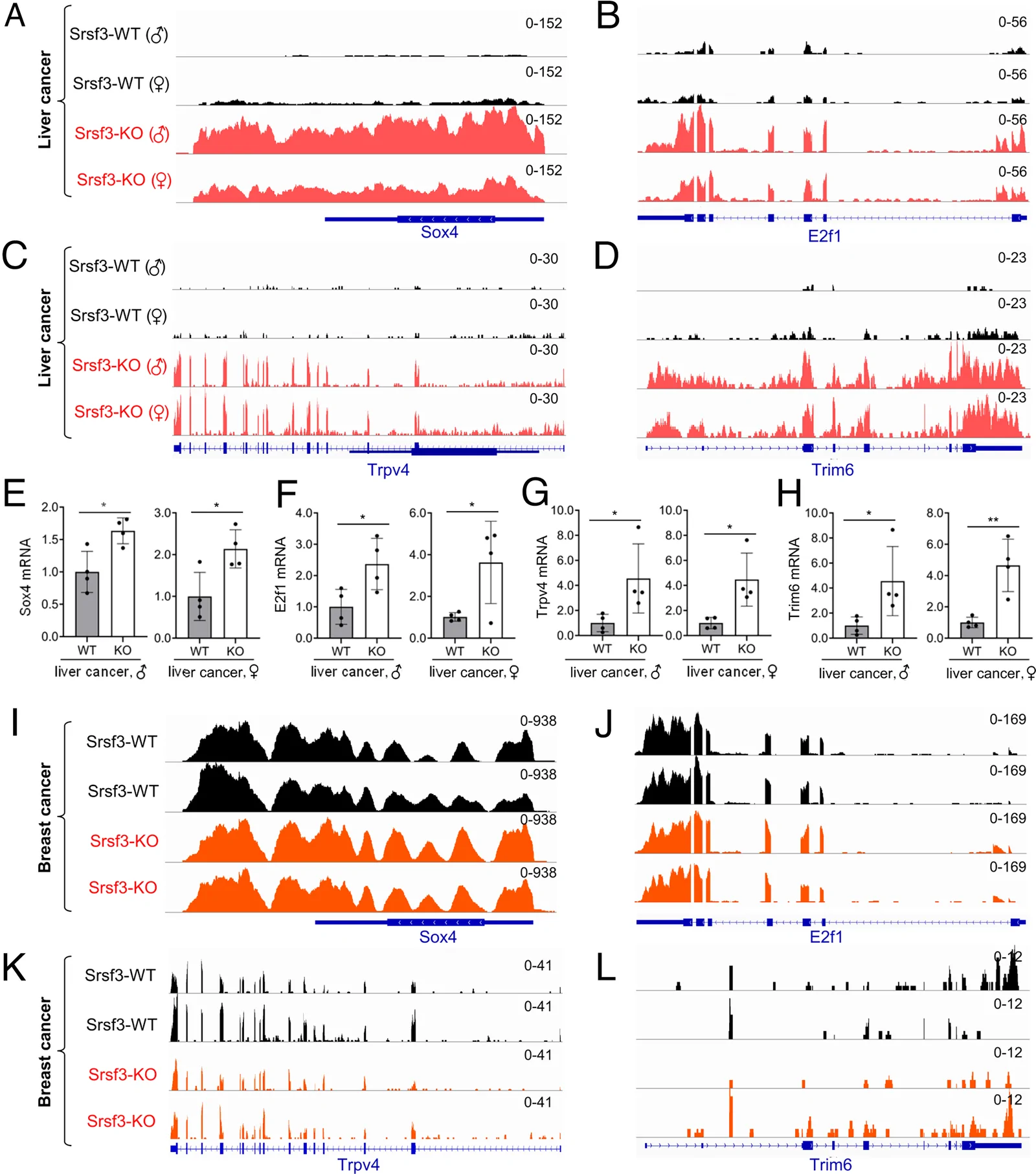
SRSF3-regulated differential expression of a subset of genes from liver to breast cancer. (*A-D*) IGV illustrations show the distribution of RNA-Seq reads mapped to Sox4, E2f1, Trpv4, and Trim6 genes in liver cancer. (*E-H*) The increased expressions of Sox4, E2f1, Trpv4, and Trim6 genes in Srsf3 KO liver cancer tissues (n=4) were validated by TaqMan RT-qPCR (*E-H*). *, *p* < 0.05; **, *p* < 0.01 by Student’s t-test. (*I-L*) IGV illustrations show the distribution of RNA-Seq reads mapped to Sox4, E2f1, Trpv4, and Trim6 genes in breast cancer.

We also analyzed the differential expressions of Sox4, E2f1, Trpv4, and Trim6, which are the genes important for cancer stem cell behavior and function (47–52) -(Fig. 2), and found that Srsf3 KO increased the expression of Sox4, E2f1, Trpv4, and Trim6 expression in DEN-induced liver cancer when compared to DEN-induced Srsf3 WT liver cancer -(Fig. 2 *A*–*D*). We further verified the increased expression of Sox4, E2f1, Trpv4, and Trim6 in DEN-induced Srsf3 KO liver cancer by RT-qPCR (Fig. 2 *E*–*H*). In contrast, Srsf3 KO had no effect on the expression of Sox4, E2f1, Trpv4, and Trim6 in the Srsf3 KO Erbb2 breast cancer when compared to the Srsf3 WT Erbb2 breast cancer -(Fig. 2 *I*–*L*). Together, these data clearly indicate that Srsf3 functions differently in a tissue-context manner by altering the expression of different genes or the same genes with opposite expression in two organs, which could partially explain the differential role Srsf3 might play in the DEN-induced liver cancer and Erbb2 breast cancer.

### Identification and Validation of Srsf3-Regulated Splicing Events from DEN-Induced Liver Cancer to Erbb2 Breast Cancer.

**Fig. 3.**
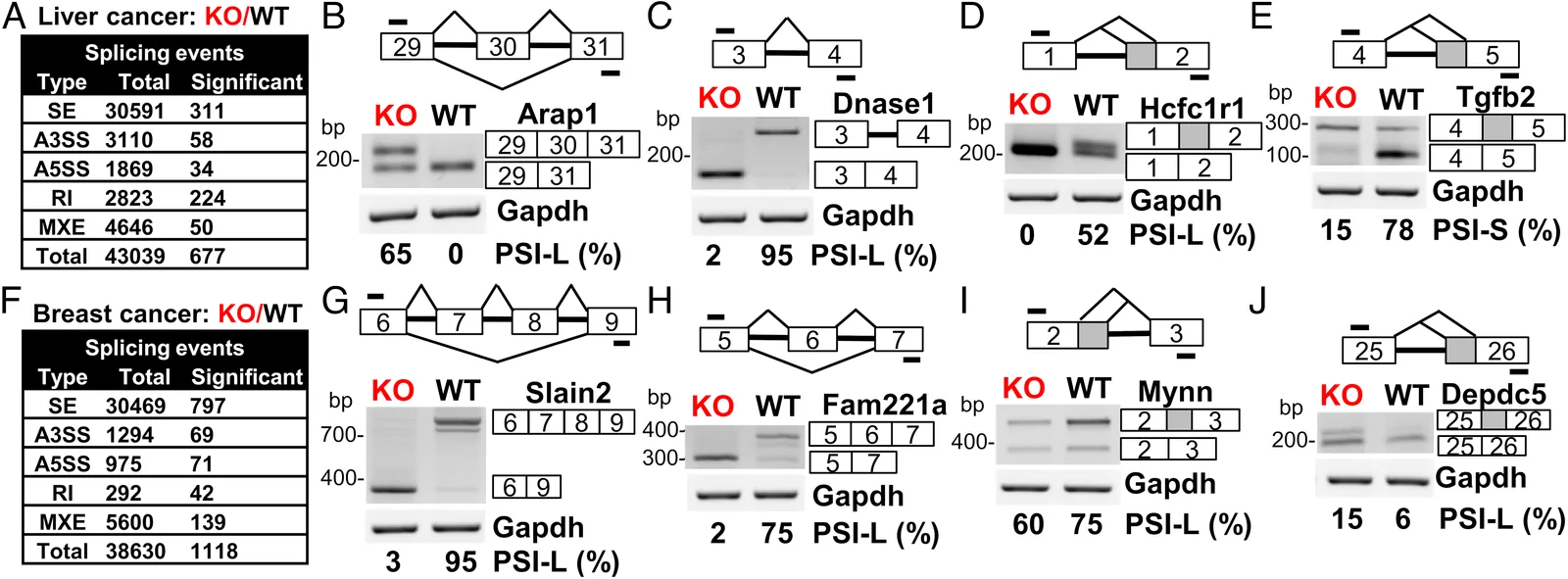
Identification and validation of differential RNA splicing events from Srsf3 KO over Srsf3 WT liver cancer and breast cancer. (*A*–*E*) Srsf3 KO regulates alternative RNA splicing events from liver Srsf3 KO cancer over Srsf3 WT cancer. (*A*) Summarized genome-wide alternative splicing events regulated by Srsf3 KO and identified by rMATS analysis (*P* ≤ 0.05, FDR ≤ 0.05) from liver Srsf3 KO over Srsf3 WT cancer. SE, skipped exon; A5SS, alternative 5′ splice site; A3SS, alternative 3′ splice site; RI, retained intron; MXE, mutually exclusive exons. (*B*–*E*) The genes highly susceptible to Srsf3 KO regulation in liver cancer tissues selectively verified by RT-PCR on exon skipping of the Arap1 exon 30 (*B*), intron 3 splicing of Dnase1 (*C*), and alternative 3′ss splicing of Hcfc1r1 exon 2 (*D*) and Tgfb2 exon 5 (*E*). (*F*–*J*) Srsf3 KO regulates alternative RNA splicing events from Srsf3 KO over Srsf3 WT breast cancer. (*F*) Summarized genome-wide alternative RNA splicing events regulated by Srsf3 KO and identified by rMATS analysis (*P* ≤ 0.05, FDR ≤ 0.05) from breast Srsf3 KO over Srsf3 WT cancer. See other details in (*A*). (*G*–*J*) The genes highly susceptible to Srsf3 KO regulation in breast cancer selectively verified by RT-PCR on exon skipping of Slain2 exons 7-8 (*G*) and Fam221a exon 6 (*H*), alternative 5′ss splicing of Mynn exon 2 (*I*), and alternative 3′ss splicing of Depdc5 exon 26 (*J*). The primers used in RT-PCR are shown as bars above (forward primers) and below (reverse primers) each RNA diagram. Gapdh RNA served as a loading control. PSI, percent spliced-in inclusion (L = long RNA isoform) or exclusion (S = short RNA isoform) of an alternative exon, intron, or splice site (% spliced-in = inclusion/sum of inclusion + exclusion for long RNA isoform or exclusion/sum of exclusion + inclusion for short RNA isoform).

To understand Srsf3-induced changes in RNA splicing in contribution to the observed opposite effect of Srsf3 KO on liver and breast carcinogenesis, we performed alternative splicing analysis using rMATS (53) and identified 677 Srsf3-regulated splicing events of 575 genes in DEN-induced Srsf3 KO over Srsf3 WT liver cancer (Fig. 3*A* and Dataset S4). We selectively validated exon skipping of Arap1 exon 30, Nprl3 exon 3, Clip1 exon 9, and Pabpc4 exon 11, retention of Dnase1 intron 3 and Kin intron 2, selection of an alternative 3′ splice site of Hcfc1r1 exon 2, Tgfb2 exon 5, and Arv1 exon 3, and selection of an alternative 5′ splice site of Zfp512b exon 8 (Fig. 3 *B*–*E* and *SI Appendix*, Fig. S9 *A*–*F*). These alternative RNA splicing changes led to altered production of the corresponding protein isoforms in DEN-induced Srsf3 KO liver cancer (*SI Appendix*, Fig. S10 *A*–*J*). The exon skipping of Pabpc4 targeted by Srsf3 was reported in our previous study (5). In addition, we found Srsf3 control of Rnf128 promoter usage and found an alternative promoter being activated in Srsf3 KO liver cancer (*SI Appendix*, Fig. S11 *A* and *B*) for expression of a shorter isoform (402 aa residues) than the authentic promoter-derived full-length (FL, 428 aa residues) Rnf128 protein, an E3 ubiquitin-protein ligase abundantly expressed in liver and other digestive tract organs (54). Two isoforms of Rnf128 appear differently on p53 degradation (55, 56). Because two alternative exon 1 are separately spliced to a common exon 2 (*SI Appendix*, Fig. S11 *A* and *B*), the short Rnf128 has the N-terminal 135 aa residues differing from the N-terminal 161 aa residues of FL Rnf128 (*SI Appendix*, Fig. S11*C*).

A total of 1,118 Srsf3-responsive splicing events in 899 genes in Srsf3 KO vs. Srsf3 WT Erbb2 breast cancers were also identified by rMATS (Fig. 3*F* and Dataset S5). We further selectively validated the Srsf3-regulated skipping of Slain2 exon 7-8, Fam221a exon 6, and Ralgapa1 exon 41, retention of Yipf2 intron 9 in the 3′ UTR region, selection of an alternative 5′ splice site in Mynn exon 2, Srsf5 exon 1 in the 5′ UTR region, and Tmem161b exon 8, selection of an alternative 3′ splice site in Depdc5 exon 26, and a cryptic intron inclusion within Srsf1 exon 4 (Fig. 3 *G*–*J* and *SI Appendix*, Fig. S9 *G*–*K*). Data indicate that these alternative RNA splicing changes led to altered production of the corresponding protein isoforms (except Yipf2 and Srsf5) (*SI Appendix*, Fig. S10 *K*–*P*). Srsf3 KO in Erbb2 breast cancer was also promoting RNA splicing of a cryptic intron within the 3′ UTR region of Srsf1 exon 4 (*SI Appendix*, Fig. S9*K*), leading to NMD of Srsf1 RNA.

### Identification of Common Srsf3-Responsive Genes with Opposite Expression Directions from DEN-Induced Liver Cancer to Erbb2 Breast Cancer.

**Fig. 4.**
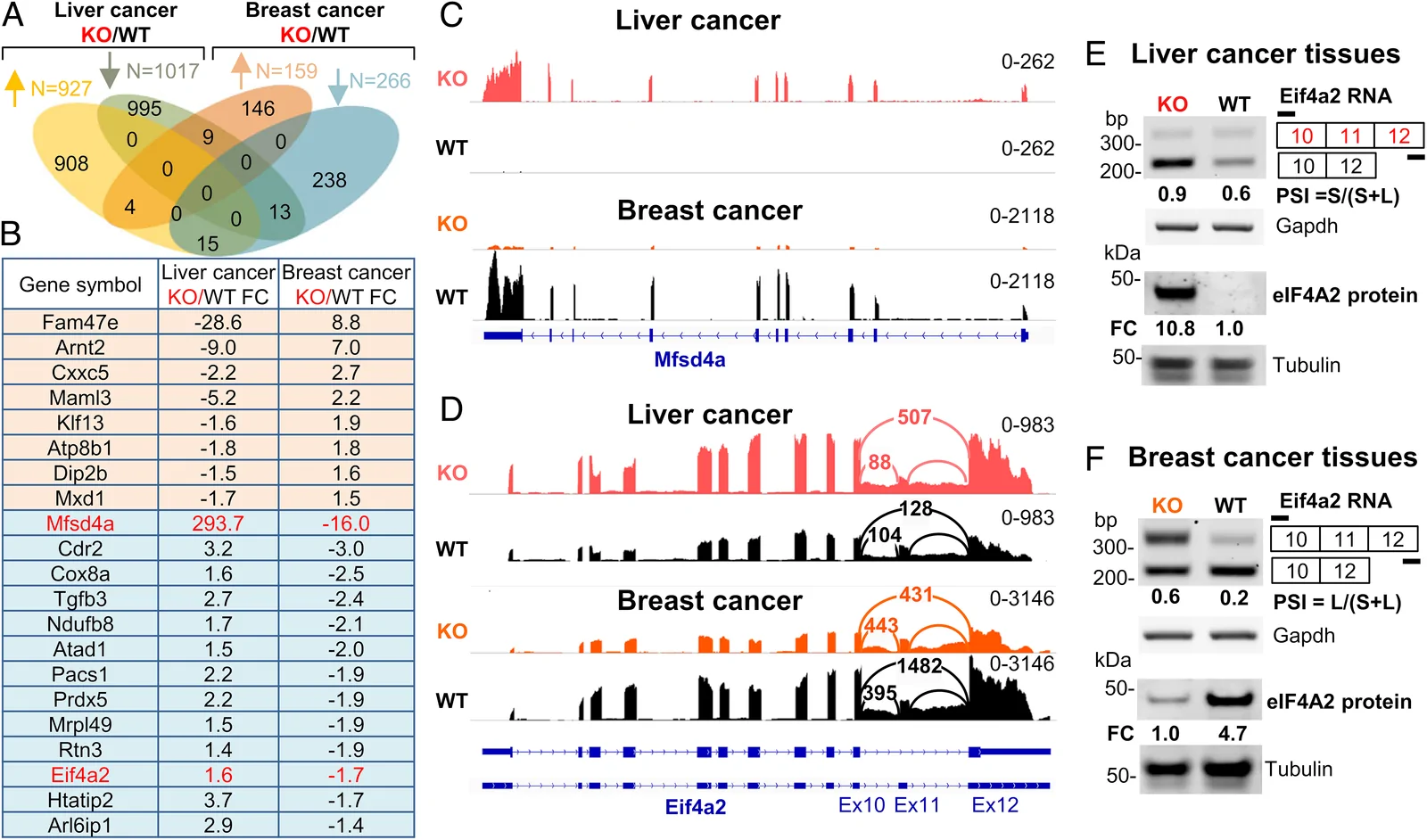
Differential expression and RNA splicing of the identified Srsf3 targets in response to Srsf3 KO from liver cancer to breast cancer. (*A*) The Venn diagram shows the overlapped genes with differential expression changes (adjusted *P* ≤ 0.05) from Srsf3 KO to WT liver cancer and breast cancer by RNA-seq analysis. (*B*) RNA-seq analyses identified 9 overlapped genes significantly downregulated by Srsf3 KO in liver cancer but upregulated in breast cancer, and 15 overlapped genes significantly upregulated by Srsf3 KO in liver cancer but downregulated in breast cancer, by fold change (FC). Genes Gm9855 in the table yellow and Gm14403 and 2610307P16Rik in the table blue color were removed due to lack of functional annotation. Eif4a2 in red was the only gene also undergoing alternative RNA splicing by Srsf3 KO in both liver and breast cancer tissues detected by rMATS. (*C* and *D*) Srsf3 KO-induced differential expression of Mfsd4a and Eif4a2 from liver cancer to breast cancer illustrated by RNA-seq reads-coverage. Srsf3 KO induced Mfsd4a and Eif4a2 expression in liver cancer but blocked the expression of Mfsd4a and Eif4a2 in breast cancer when compared to its Srsf3 WT cancer tissues. The numbered arches (*D*) represent splice junction reads detected in Sashimi plots. (*E* and *F*) Alternative splicing validation of Eif4a2 RNA by RT-PCR with an indicated primer pair (short lines) above exon 10 and below exon 12 and Western blot analysis of Eif4A2 protein expression in Srsf3 KO and WT liver cancer (*E*) and breast cancer (*F*) tissues. Srsf3 KO reduced the exon 11 inclusion and increased Eif4a2 protein expression in liver cancer (*E*) but promoted the exon 11 inclusion in Eif4a2 RNA splicing and thereby inhibited Eif4a2 protein expression in breast cancer (*F*), when compared to its corresponding Srsf3 WT cancer tissues, with Gapdh RNA served as a loading control (*E* and *F*). PSI, percent spliced-in of the alternative exon(s), was used to quantify alternative exon inclusion or exclusion. S, small size band; L, large size band. In Western blot analysis, the FC of Eif4a2 protein between Srsf3 KO and WT cancer tissues was calculated after being normalized to β-tubulin.

Although the difference in Srsf3 KO-induced gene expression profiles identified above could be most likely resulted from the different organs (liver vs. breast) and different carcinogens (DEN vs. Erbb2) used in the tumor induction, we further investigated the common Srsf3-responsive genes to explore how Srsf3 at its physiological level plays its differential roles in the development of DEN-induced liver cancer as a tumor suppressor and of Erbb2 breast cancer as an oncogene. By Venn diagram analysis (Fig. 4*A*), we identified 41 genes commonly responsive to Srsf3 KO, with 15 genes significantly upregulated in their expression in liver cancer but downregulated in breast cancer, and 9 genes significantly downregulated in their expression in liver cancer upon Srsf3 KO but upregulated in breast cancer (Fig. 4*B*). The remaining 17 genes were common Srsf3 KO responders with the same expression trend, of which 4 genes were upregulated, and 13 genes were downregulated. Major facilitator superfamily domain containing 4A protein (Mfsd4a) is a tumor suppressor (57, 58) involved in glucose transmembrane transport and was on the top list of dysregulated genes in their expression. Mfsd4a was up-regulated 293-fold in DEN-induced Srsf3 KO liver cancer, but down-regulated 16-fold in Srsf3 KO Erbb2 breast cancer (Fig. 4*B*). This opposite response of Mfsd4a expression to Srsf3 KO from DEN-induced liver cancer to Erbb2 breast cancer was remarkable by IGV visualization of RNA-seq reads-coverage (Fig. 4*C*) and easily validated by NanoString analysis (*SI Appendix*, Fig. S7 *B* and *D*). Similarly, Htatip2 (Tip30), a tumor suppressor in various cancers (59–61), was upregulated in DEN-induced liver cancer, but downregulated in Erbb2 breast cancer following Srsf3 KO (*SI Appendix*, Fig. S12*A*). Srsf3 KD in Hepa1-6 cells also promoted the expression of Htatip2 (*SI Appendix*, Fig. S12 *B* and *C*).

Eukaryotic translation initiation factor 4A2 (Eif4a2 or DDX2B), a protumor RNA helicase (62, 63), was identified as another Srsf3 KO responder, of which expression was upregulated in DEN-induced Srsf3 KO liver cancer but downregulated in Srsf3 KO Erbb2 breast cancer (Fig. 4*C*). Eif4a2 gene has 12 exons and its exon 11 is a “poison exon.” Inclusion of exon 11 in Eif4a2 RNA leads to Eif4a2 RNA degradation by NMD (64, 65). Only the Eif4a2 mRNAs without exon 11 encode a full-length (FL) Eif4a2 protein. Therefore, inclusion of the exon 11 in Eif4a2 RNA splicing will reduce the expression of FL Eif4a2 protein. We found by RNA-seq that DEN-induced Srsf3 KO liver cancer exhibited more exon 11 exclusion and enhanced the expression of Eif4a2 RNA (Fig. 4*D*). In contrast, Srsf3 KO Erbb2 breast cancer displayed more Eif4a2 exon 11 inclusion and reduced Eif4a2 RNA level due to induction of Eif4a2 RNA degradation by NMD (Fig. 4*D*). RT-PCR analyses of the total RNA from DEN-induced liver cancer and Erbb2 breast cancer tissues and western blot analyses of their tissue lysates, respectively, confirmed Srsf3 KO promoting exon 11 exclusion and increasing the production of eIF4A2 protein in the DEN-induced liver cancer (Fig. 4*E*), but exon 11 inclusion and decreasing eIF4A2 protein production in the Erbb2 breast cancer (Fig. 4*F*). Consistently with Erbb2 cancer tissues, knockdown of Srsf3 expression in mouse c-neu^+^ NF639 cells and two human breast cancer cell lines ER^+^ MCF7 and HER2^+^ SKBR3 cells was also found to promote exon 11 inclusion of Eif4a2 by RT-PCR analysis of total cell RNA and subsequent reduction of eIF4A2 protein by immunoblotting of cell lysates (*SI Appendix*, Fig. S12*D*).

Srsf3 KO also affected the opposite expression of other genes involved in RNA export and translation (Datasets S4 and S5). These were Srsf3 KO-mediated reduction of RNA export factor Aly/REF in DEN-induced liver cancer but not in Erbb2 breast cancer, and Srsf3 KO reduced expression of nuclear export factor NXF1 in Erbb2 breast cancer but not in DEN-induced liver cancer. Besides translation initiation factor eIF4a2, Srsf3 KO also decreased the expression of eIF4ebp2 in DEN-induced liver cancer and eIF1ad and eIF3A in Erbb2 breast cancer but increased the expression of eIF4e only in DEN-induced liver cancer.

### Srsf3 KO-Mediated Reduction of ERα and Foxa Responsible for Sex Disparity in the Development of DEN-Induced Srsf3 KO Liver Cancer.

Liver cancer incidence and death in men are significantly higher than in women (32). Male mice are prone to the development of liver cancer over female mice induced by chemical carcinogen (34, 66). Estrogen and estrogen receptor ERα appear to be protective for female from suffering liver cancer (33, 34, 67). Foxa gene family members also contribute to sex disparity in the development of liver cancer and female Foxa1/2-deficient livers are prone to HCC as male livers. Thus, ERα and Foxa gene family are assumed to coregulate the certain genes responsible for female mouse resistance to liver cancer induction by DEN (35). Consistent with the previous report, our result also showed higher DEN-induced liver cancer incidence in Srsf3 WT male mice than in Srsf3 WT female mice (*SI Appendix*, Fig. S5*A*). Surprisingly, we found that Srsf3 KO remarkably reduced the gender disparity of DEN-induced liver cancer incidence between male and female mice (*SI Appendix*, Fig. S5*B*).

**Fig. 5.**
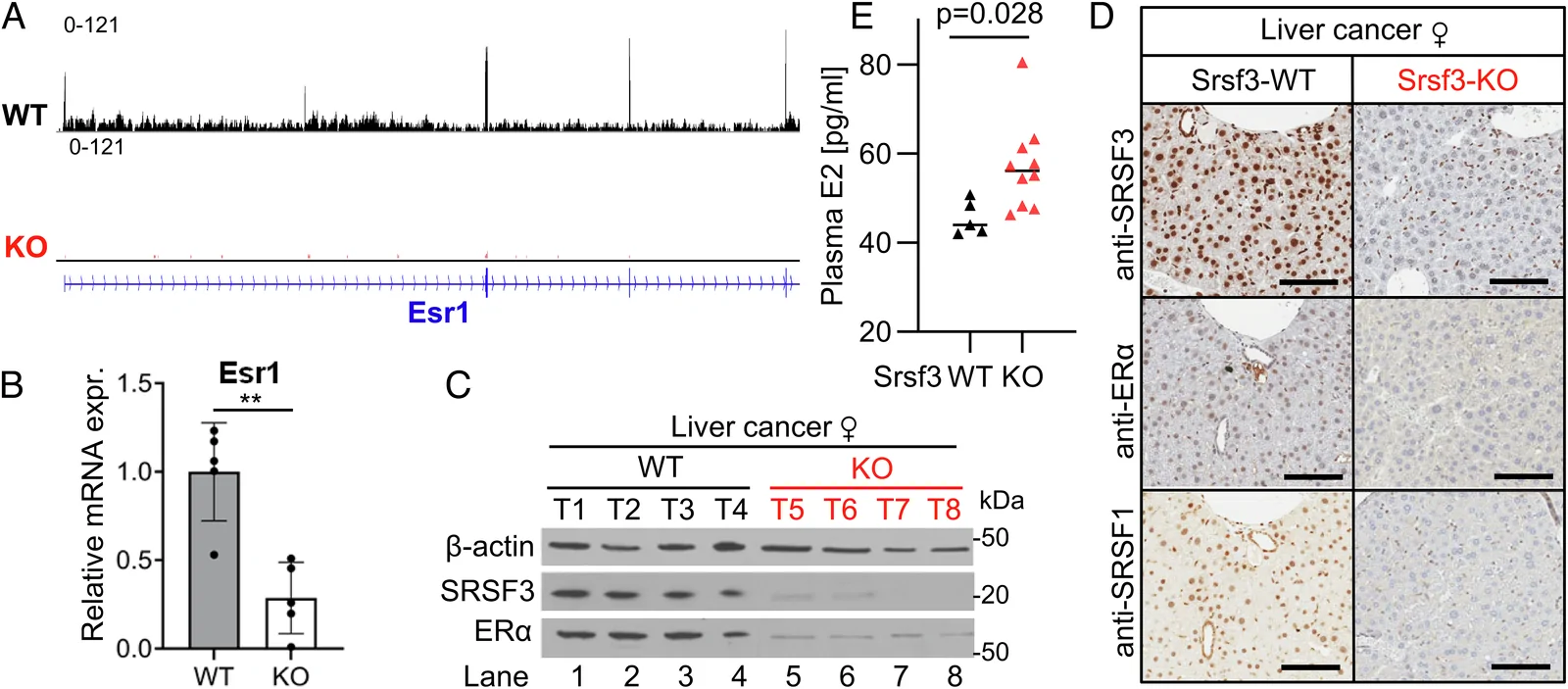
Srsf3 KO disrupts liver cancer sex disparity by blocking transcription of mouse Esr1 gene and reduces the production of ERα protein in female hepatocytes. (*A* and *B*) Srsf3 KO reduces the production of mouse liver Esr1 RNA (encoding ERα protein) from the Esr1 gene as illustrated by IGV RNA-seq reads-coverage (*A*) and verified by RT-qPCR of RNA samples extracted separately from five Srsf3 WT and five Srsf3 KO liver cancer tissues (*B*). **P < 0.01, Student’s t test. (*C*) Srsf3 KO decreases the expression of liver ERα protein in female (♀) mice. Four tumor (T) tissue extracts in each group (T1–T4 for Srsf3 WT and T5–T8 for Srsf3 KO) were examined for SRSF3 and ERα proteins by Western blot using a corresponding antibody as indicated. β-actin served as a protein loading control. (*D*) Immunohistology staining of SRSF3, ERα, and SRSF1 protein in female (♀) Srsf3 WT and Srsf3 KO liver cancer tissues. One of three pairs of representative samples in each group were shown (Scale bar, 100 µm). (*E*) Srsf3 KO led to increased levels of plasma estradiol (E2) in liver-specific Srsf3-KO female mice (P = 0.028, Student’s t test) by blocking liver expression of ERα. Five Srsf3 WT and 10 Srsf3 KO female mice at 12 mo of DEN injection were examined for their plasma E2 level by ELISA assays.

**Fig. 6.**
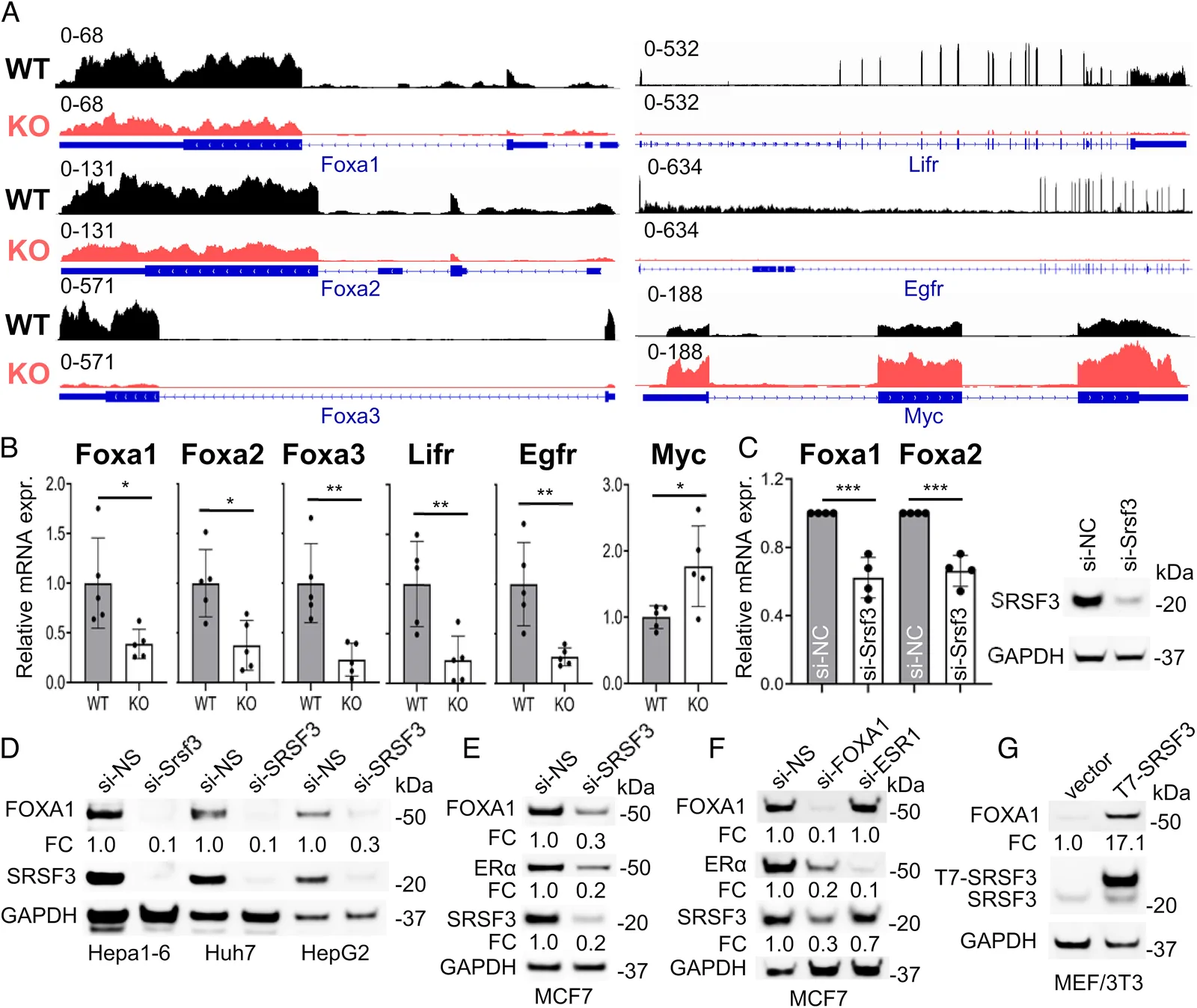
Srsf3 KO in female mouse liver disrupts sex disparity in development of liver cancer by reducing the expression of Foxa1, Foxa2, and Foxa3, Lifr, and Egfr, but increasing Myc expression. (*A*) Srsf3 KO decreases RNA expression of Foxa1, Foxa2, Foxa3, Lifr, and Egfr but increases Myc RNA. RNA-seq reads-coverage of indicated genes in female Srsf3-WT (black) and Srsf3-KO liver tumor (red) were visualized by IGV. (*B*) Validation of RNA expression for Foxa1, Foxa2, Foxa3, Lifr, Egfr, and Myc in female Srsf3-WT and Srsf3-KO liver cancer tissues by RT-qPCR. Five samples in each group were examined. (*C*) Hepatocyte Foxa RNA expression depends on Srsf3 and KD of Srsf3 expression in a mouse liver cancer cell line Hepa1-6 reduces RNA expression of Foxa1 and Foxa2. Hepa1-6 cells were transfected with 40 nM of siRNA. Total RNA and protein were harvested at 48 h after transfection and measured by RT-qPCR (*C*, bar graph) or Western blot for Srsf3 KD efficiency (*C*, *Left* panel). The data were averaged from four independent experiments. *P < 0.05; **P< 0.01; ***P< 0.001 by Student’s t test (*B* and *C*). (*D* and *E*) SRSF3 is essential for expression of FOXA1 in liver cancer cells (mouse Hepa1-6, human Huh7, and HepG2) and of both FOXA1 and ERα in human ERα-positive breast cancer MCF7. SRSF3 KD in liver cancer cells and MCF7 decreases the protein expression of FOXA1 (*D* and *E*) and ERα (*E*). (*F*) FOXA1 KD in MCF7 cells reduces the protein expression of ERα and SRSF3, but ESR1 KD does not affect FOXA1 expression and only minimally on SRSF3. Gene-specific siRNA KD in the indicated cell lines (*D*–*F*) was performed by two transfections of 40 nM of the indicated siRNA at a 48-h interval (0 and 48 h). Cell lysates were harvested at 96 h for Western blot analysis. (*G*) Inducible expression of Foxa1 protein in SRp20 (SRSF3) MEF/3T3 Tet-Off cells. T7-SRp20 (SRSF3) MEF/3T3 Tet-Off cells under control by doxycycline (20) were grown in doxycycline-free DMEM for inducible T7-SRp20 (SRSF3) expression. Cell lysates were collected at the third passage for western blot analysis. GAPDH served as a loading control (*C*–*G*). FC was calculated after normalization to GAPDH (*D*–*G*).

To investigate how Srsf3 KO led to reduction of gender disparity in DEN-induced liver cancer, we examined potential effect of Srsf3 KO on estrogen signaling (33, 34, 67) (Fig. 5) and expression of Foxa family genes (35) (Fig. 6). RNA-seq analyses and RT-qPCR validation showed a significantly reduced expression of ERα RNA from Esr1 gene (Fig. 5 *A* and *B*) in DEN-induced Srsf3 KO liver cancer than DEN-induced Srsf3 WT liver cancer in female mice. Western blot analysis (Fig. 5*C*) and immunostaining (Fig. 5*D*) of DEN-induced liver cancer tissues also showed a decreased level of ERα protein in the Srsf3 KO female hepatocytes. Interestingly, the Srsf3 KO-mediated reduction of ERα protein in hepatocytes led to an increased level of plasma estradiol E2 in Srsf3 KO female mice than the corresponding Srsf3 WT female mice (Fig. 5*E*), suggesting the impaired uptake of estradiol E2 by hepatocytes in the Srsf3 KO female mice. Srsf3 KO mediated reduction of ERα expression was not limited to female, but also to male hepatocytes (*SI Appendix*, Fig. S13 *A–C*). When SRSF1, a sensitive SRSF3 responder (*SI Appendix*, Fig. S9*K*) (7), was used as a positive control, we found that hepatocyte Srsf3 KO in both male and female liver cancer and breast cancer displayed, as expected, a reduced expression of SRSF1 (Fig. 5*D* and *SI Appendix*, Fig. S13).

Cancer-related leukemia inhibitory factor receptor Lifr, epidermal growth factor receptor Egfr, and oncogene Myc are three downstream genes coregulated by ERα and Foxa for their expression (35). Our RNA-seq and RT-qPCR validation of DEN-induced Srsf3 WT and Srsf3 KO liver cancer demonstrated a significantly decreased expression of Foxa1 (also known as hepatocyte nuclear factor 3), Foxa2, and Foxa3, and Lifr and Egfr, but increased expression of Myc (68) in DEN-induced Srsf3 KO liver cancer in both female (Fig. 6 *A* and *B*) and male mice (*SI Appendix*, Fig. S14). Analyses of the TCGA datasets show that high SRSF3 level is associated with decreased liver HCC (LIHC) survival (*SI Appendix*, Fig. S15*A*), a positive coexpression with FOXA1 in LIHC and LIHC-adjacent normal tissues (*SI Appendix*, Fig. S15*B*), and a positive correlation of FOXA1 and ERα in the adjacent normal livers (*SI Appendix*, Fig. S15*C*). Consistently, mouse Srsf3 KO liver cancer with a reduced Foxa1 had further reduction of Esr1 expression (*SI Appendix*, Fig. S15*D*). We further confirmed the reduction of Foxa1/2 RNA expression after Srsf3 KD in a mouse liver cancer cell line Hepa1-6 (Fig. 6*C*) and of FOXA1 protein expression from Hepa1-6 cells and two human liver cancer cell lines, Huh7 and HepG2 cells (Fig. 6*D*). As these three liver cancer cell lines do not express detectable ERα (*SI Appendix*, Fig. S16*A*) by Western blot (69), we chose a human breast cancer cell line, ERα^+^ MCF7 cells (*SI Appendix*, Fig. S16*A*), and demonstrated that SRSF3 KD in MCF7 cells reduces ERα expression, in addition to FOXA1 (Fig. 6*E*). Altogether, our data indicate that SRSF3 at its physiological level is essential for expression of FOXA1/2/3 and ERα, subsequently, Lifr, Egfr, and Myc, and thereby is responsible for sex disparity in the development of DEN-induced liver cancer in the presence of estrogen.

### SRSF3 Regulates the Expression of FOXA1 and ERα Presumably Via Transcription.

Both FOXA1 and ERα are nuclear transcription factors crucial for expression of hormone-responsible genes, organ development and animal reproduction, of which FOXA1 and ERα mutually affect their recruitment and DNA binding to its cis-regulatory elements (35, 70–72). We next examined this mutual regulation in MCF7 cells and found that FOXA1 controls both ERα and SRSF3 expression evidenced by their reduction upon FOXA1 KD but did not see ERα KD-induced reduction of FOXA1 expression and only minimal on SRSF3 (Fig. 6*F*). Data indicate the existence of a feedback loop in this regulation mainly between SRSF3 and FOXA1.

To provide mechanistic evidence supporting SRSF3 regulation of FOXA1 and ERα expression, we first aimed to generate SRSF3-deficient hepatocytes in vitro to examine whether exogenous SRSF3 could rescue FOXA1 expression. Unfortunately, SRSF3 KD inhibits cell proliferation and induces cell apoptosis (*SI Appendix*, Fig. S16 *B–D*) as we reported (20). However, hepatoblastoma-derived HepG2 (73, 74) appeared resilient to SRSF3 KD but displayed distinguishable cell spread-out morphology and increased lipid accumulation upon SRSF3 KD when compared with HCC-derived Hepa1-6 and Huh7 cells (*SI Appendix*, Fig. S17) as reported (75). Consequently, HepG2 cells were chosen for the rescue experiments and showed that overexpressed FLAG-SRSF3 could rescue FOXA1 expression in the SRSF3-deficient cells (*SI Appendix*, Fig. S16*E*). By using doxycycline-inducible T7-SRp20 (SRSF3) MEF/3T3 cells (20), we obtained a 17-fold enhancement of FOXA1 expression by exogenous T7-SRSF3 in the cells (Fig. 6*G*).

**Fig. 7.**
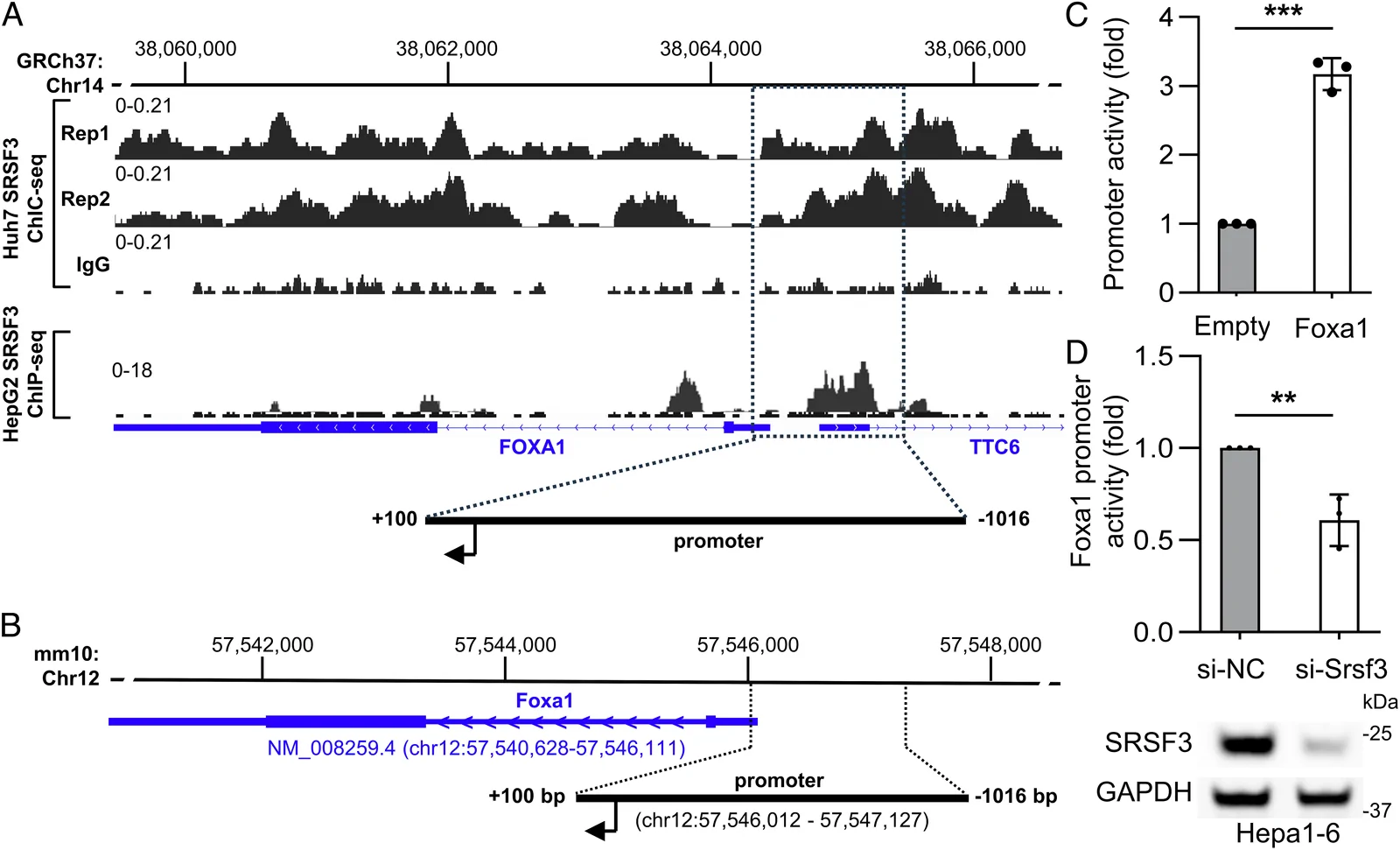
SRSF3 binds Foxa1 promoter and regulates Foxa1 promoter activity. (*A*) SRSF3 binds the FOXA1 promoter region in Huh7 and HepG2 cells. Chromatin immune-cleavage sequencing (ChIC-seq) data generated from Huh7 cells (NCBI GSE331053) using an anti-SRSF3 antibody (Abcam, Cat. # ab125124) were used to profile reads-distribution around the FOXA1 promoter region. The libraries were sequenced by Nova-X and aligned to the reference genome using Bowtie2 with default parameters. Genome-wide coverage tracks with uniquely mapped reads were generated using normalized reads counts (CPM). Rep 1 and Rep2 in ChIC-seq were two separate repeats with anti-SRSF3 antibody along with IgG control. The HepG2 ChIP-data originated from NCBI GSE120104 Sample GSM3393528 (77) using anti-SRSF3 antibody (MBL Cat.# RN080PW) was used to profile reads-distribution around the FOXA1 promoter region. (*B*) Schematic of the Foxa1 gene (blue) in the mouse genome (mm10). The Foxa1 promoter region (black line), spanning −1,016 to +100 bp relative to the annotated transcription start site (arrow) from Hepa1-6 cells, was cloned upstream of a Firefly luciferase reporter in a plasmid pVM174 (*SI Appendix*, Fig. S19 *B* and *C*). (*C*) The cloned Foxa1 promoter region displays promoter activities in promoting Firefly luciferase expression. The promoter activity in pVM174 was compared with a promoter-less (empty) vector pGL3-basic in transfected Hepa1-6 cells together with cotransfection of a Renilla luciferase expression plasmid pRL-TS (86) using a dual-luciferase assay and normalizing to Renilla luciferase activity. (*D*) SRSF3 is necessary for Foxa1 promoter activity. SRSF3 on Foxa1 promoter activity was evaluated in Hepa1-6 cells by single transfection of 40 nM of a nontargeting siRNA (si-NC) or SRSF3-specific siRNA (si-Srsf3). The cells at 24 h after siRNA treatment were transfected by the pVM174. The promoter activity was measured 24 h after plasmid transfection by luciferase assays and normalized to total cell number determined by CCK-8 (Cell Counting Kit-8) assay. The immunoblot (below) shows Srsf3 knockdown efficiency. Data are presented as mean ± SD from three independent experiments. Statistical significance was assessed using Student’s t test (***P < 0.001; **P < 0.01).

Given that RNA-seq reads-coverage showed a uniformed RNA reads-reduction of ERα (Fig. 5*A*) and Foxa in Srsf3 KO liver cancer (Fig. 6*A*), we assume Srsf3 KO in hepatocytes most likely prevents transcription of Esr1 and Foxa genes and their downstream targets. Because FOXA1 binding to the Esr1 promoter by ChIP-seq in estrogen-treated MCF7 cells has been reported (76) (*SI Appendix*, Fig. S18), we examined SRSF3 interaction with FOXA1 promoter and revealed SRSF3-specific association with the FOXA1 promoter region in Huh7 cells by using ChIC-seq and in HepG2 cells by using ChIP-seq (Fig. 7*A*). The same HepG2 region was also associated with RNA polymerase II subunit G and other RBPs (*SI Appendix*, Fig. S19*A*) (77). By inserting the 1.2 kb DNA fragment from mouse Foxa1 promoter region (1,016 nts upstream and 100 nts downstream of the Foxa1 transcription start site) into a luciferase reporter (Fig. 7*B* and *SI Appendix*, Fig. S19 *B* and *C*), we subsequently demonstrated that Foxa1 promoter activity requires cellular Srsf3 in Hepa1-6 cells because Srsf3 KD significantly reduced Foxa1 promoter activity in the assays (Fig. 7 *C* and *D*).

## Discussion

**Fig. 8.**
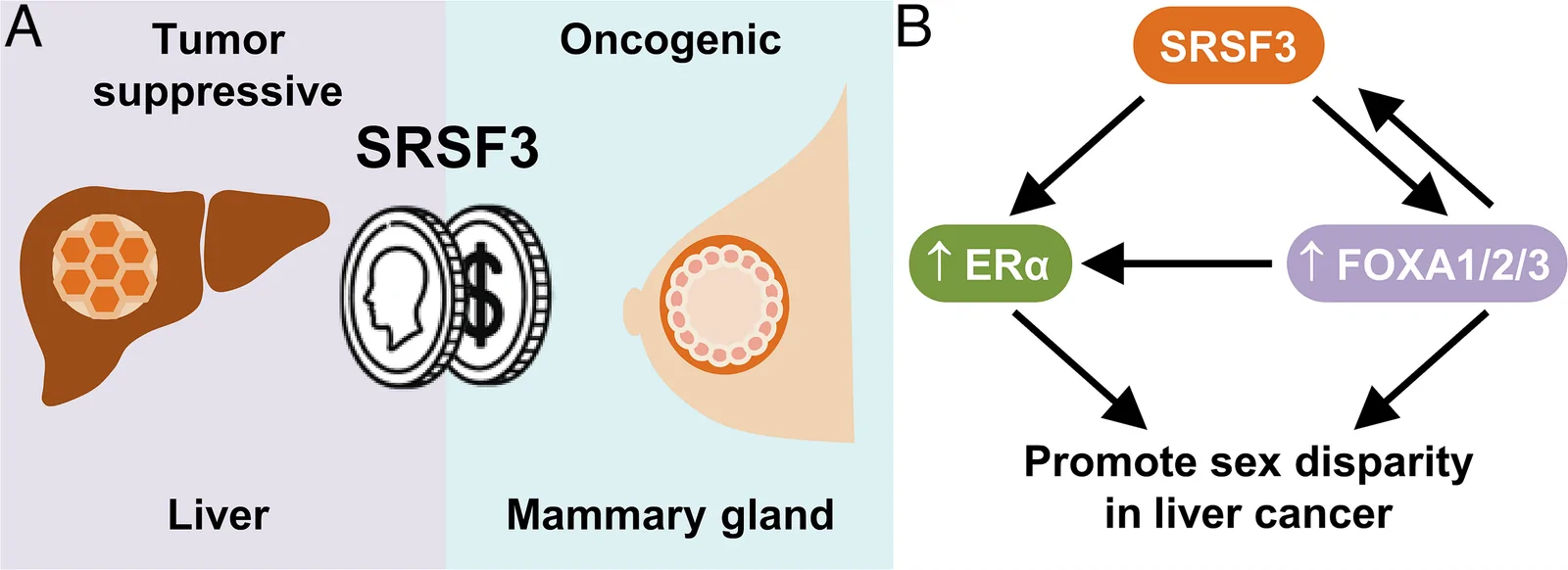
Diagram illustration of SRSF3 functions in cancer development. (*A*) SRSF3 behaves as one coin with two sides and has dual roles in cancer development. SRSF3 functions as a tumor suppressor promoting sex disparity in the development of DEN-induced liver cancer but acts as an oncogene in mammary glands enhancing breast carcinogenesis in Erbb2 mice. Diagrams highlight SRSF3 exhibiting context-dependent and tissue-specific functions in tumorigenesis. (*B*) SRSF3 acts as a key regulator in the expressions of ERα and FOXA family genes (FOXA1, FOXA2, and FOXA3) to promote sex disparity in development of liver cancer.

As an important splicing factor, SRSF3 has profound functions in health and its aberrant expression has been associated with many diseases and cancers (4). In this report, we further demonstrated that SRSF3 even at its physiological level functions as a tumor-suppressor in DEN-induced liver cancer, but a tumor-promotor in the development of Erbb2 breast cancer through differential regulation of RNA transcription and alternative RNA splicing of a different set of genes in two different organs (Fig. 8*A*). This surprising observation makes SRSF3 as another gene which functions with two different faces in a tissue- or cell type–dependent manner for its differential tumor suppressive or oncogenic potentials, as reported for TGF beta (78, 79), NF-kB (80), PTEN (81, 82), PML (83, 84), BRD4 (85), etc.

SRSF3 is tumor suppressive in DEN-induced liver cancer. We found that Srsf3 KO in mouse hepatocytes could promote the development of DEN-induced liver cancer, consistent with the report of Srsf3 KO enhancing liver cancer incidence in aged mice (38). Srsf3 at a physiological level is critical for hepatocyte maturation and metabolic function (36) and prevention of DNA damage (87). Interestingly, despite its tumor suppressive function, SRSF3 is highly expressed in HCC patients (88). We found an increased SRSF3 protein expression in 110 HCC samples (grade 2 and 3) (*SI Appendix*, Fig. S1*A*), as seen in many other cancer tissues (4, 7, 20). The increased expression of SRSF3 is essential for cancer cell proliferation (20) and shorter LIHC survival, but loss of SRSF3 expression leads to cell apoptosis (20) and senescence (21), and genome instability (87). The increased expression of tumor-suppressors (89) is common in many cancer types, including CDK inhibitor p16Ink4a in glioma and HPV-induced cancers (90–92), CDK4/6 inhibitor p18Ink4c in cervical cancer and glioblastoma (93, 94), and CDK2 and PCNA inhibitor p21 in many types of cancers (95).

However, the increased expression of SRSF3 is correlated with breast cancer progression and 5-y overall survival (20), making our initial discovery of SRSF3 as a proto-oncogene responsible for cancer cell proliferation and tumor maintenance (20). SRSF3 is responsible for 23% of alternative RNA splicing events in 682 invasive breast ductal carcinoma patients and regulates HER2 RNA splicing to produce oncogenic Δ16HER2 (96, 97). Higher expression of SRSF3 was linked with shorter survival time and poorer prognosis (98). Consistently, conditional Srsf3 KO in mouse mammary glands harmed c-neu (Erbb2) expression and the development of Erbb2 breast cancer. RNA-seq profiling showed Srsf3 regulation of mammary gland expression of 425 genes including Mfsd4a, Pcbp4, Ahnak2, and Ctif, and of 1,118 RNA splicing events in 899 genes, including exon exclusion of Slain2, Fam221a, and Ralgapa1, intron splicing of Yipf2, and alternative 5′ or 3′ splice site usage of Mynn, Depdc5, and Tmem161b. Altogether, our data indicate that the altered gene transcription and alternative RNA splicing by conditional Srsf3 KO in mouse mammary glands were the contributing factors for reduction of Erbb2 breast cancer.

Srsf3 in mouse hepatocytes was found to regulate the expression of 1,944 genes and 677 splicing events in 575 genes, including the expression of Hnf1α, Hmgcr, Srebp1, Srebp2, and Scap, and alternative RNA splicing of Fn1, Myo1b, Insr, which were previously reported (36–38). Srsf3 KO in mouse hepatocytes, but not in mouse mammary glands, promoted the expression of Sox4, E2f1, Trpv4, and Trim6, and alternative RNA splicing of Arap1, Pabpc4, Clip1, Hcfc1r1, Tgfb2, and Kin. We identified that 24 out of 41 genes responsive to Srsf3 KO exhibited differentially opposite expression directions between liver cancer and breast cancer. Srsf3 KO in mouse hepatocytes increased the expression of tumor suppressor Mfsd4a (57) and protumor RNA translation initiator Eif4a2 (62, 63), but decreased their expression in mouse mammary glands. Others, including Cox8a, Ndufb8, and Atad1, which are the genes involved in oxidative phosphorylation pathway (37), and nuclear export factor NXF1 and Aly/REF (99, 100), also exhibited striking opposite regulation directions by Srsf3 KO from DEN-induced liver cancer to Erbb2 breast cancer. These gene expression patterns exemplify how Srsf3 could act as a tumor suppressor in DEN-induced liver cancer, but a proto-oncogene in Erbb2 breast cancer. Thus, our comparative studies by using two different cancer models could illustrate such sharp contrast regulation and outcomes by Srsf3, which would be buried or misinterpreted if only one cancer model was applied.

Liver cancer has an incidence and mortality rate twice higher in men than in women (32). Male mice are also prone to the development of liver cancer over female mice in DEN-induced liver carcinogenesis (34). Surprisingly, we showed that Srsf3 KO decreases gender disparity in the development of liver cancer. It has been known that loss of ERα or Foxa1 and Foxa2 expression in female liver increases DEN-induced liver cancer in female mice (34, 35). What controls the expression of Foxa family genes remains unknown. Surprisingly, Srsf3 KO in our study decreases the expression of both ERα and Foxa1-3 and Foxa downstream genes Lifr and Egfr but increases oncogenic Myc expression both in female and male livers. Further studies showed that SRSF3 may transcriptionally control the expression of FOXA family and ESR1 genes (Fig. 8*B*) with a feedback loop of this regulation between SRSF3-FOXA1. This presumption was based on 1) SRSF3 specifically associating with the FOXA1 promoter region, 2) this SRSF3-association with this region overlapping with POL2RG and other RBPs (77), 3) SRSF3 being responsible for FOXA1 promoter activity in luciferase assays. Altogether, we believe that SRSF3, FOXA, ERα, LIFR, EGFR, and Myc are all contributing factors to sex disparity in the development of liver cancer.

## Materials and Methods

### Tissue-Specific Srsf3 KO, Tumor Induction, Pathology, and Biochemical Analyses.

Liver-specific Srsf3-KO mice were generated by crossing Srsf3-floxed mice with Alb-Cre transgenic mice. Liver cancer was induced by a single i.p. DEN injection. Mammary-specific Srsf3-KO mice were generated by crossing Srsf3-floxed mice with MMTV-Cre mice and further outbred with MMTV-Erbb2 transgenic mice for mammary tumorigenesis. Tumor development was monitored weekly by palpation for 18 mo. Tissues and blood were collected for histopathology and liver function analyses, respectively.

### RNA-Sequencing, Bioinformatics, and Validation.

Liver and mammary tissue total RNA-seq were performed by Illumina platform and the sequence reads obtained were mapped to the mouse mm10 genome, followed by differential gene expression and alternative splicing analyses, and then validated by NanoString nCounter assays, RT-qPCR, immunoblotting, and immunohistochemistry.

### Gene Functional Assays.

These studies were conducted in mouse Hepa1-6 and NF639 cells and human Huh7, HepG2, SKBR3, and MCF7 cells by siRNA and immunoblotting assays. Cell proliferation, lipid accumulation, gene expression, and RNA splicing were evaluated, respectively, by CCK-8 assays, Oil Red O staining, RT-PCR, 5′RACE, and RT-qPCR. SRSF3 regulation of Foxa1 expression was investigated by ChIC-seq, ChIP-seq, and promoter-reporter assays. Statistics were Student’s *t* test, Fisher’s exact test, two-way ANOVA, and Kaplan–Meier survival analysis.

Additional experimental details are provided in *SI Appendix*, *Materials and Methods*.

## Supplementary Material

Appendix 01 (PDF)

Dataset S01 (XLSX)

Dataset S02 (XLSX)

Dataset S03 (XLSX)

Dataset S04 (XLSX)

Dataset S05 (XLSX)

Dataset S06 (XLSX)

## Data Availability

Raw data of RNA-seq and ChIC-seq supporting the findings in this study have been deposited in the NCBI GEO database under accession numbers GSE276011 (RNA-seq) (101) and GSE331053 (ChIC-seq) (102).

## References

[1] R. K. Bradley, O. Anczuków, RNA splicing dysregulation and the hallmarks of cancer. Nat. Rev. Cancer **23**, 135–155 (2023). 10.1038/s41568-022-00541-7 PMC10132032 36627445

[2] O. Anczuków, A. R. Krainer, Splicing-factor alterations in cancers. RNA **22**, 1285–1301 (2016). 10.1261/rna.057919.116 PMC4986885 27530828

[3] C. J. David, J. L. Manley, Alternative pre-mRNA splicing regulation in cancer: Pathways and programs unhinged. Genes Dev. **24**, 2343–2364 (2010). 10.1101/gad.1973010 PMC2964746 21041405

[4] R. Jia, Z. M. Zheng, Oncogenic SRSF3 in health and diseases. Int. J. Biol. Sci. **19**, 3057–3076 (2023). 10.7150/ijbs.83368 PMC10321290 37416784

[5] R. Jia, M. Ajiro, L. Yu, P. McCoy Jr., Z. M. Zheng, Oncogenic splicing factor SRSF3 regulates ILF3 alternative splicing to promote cancer cell proliferation and transformation. RNA **25**, 630–644 (2019). 10.1261/rna.068619.118 PMC6467003 30796096

[6] M. Ajiro, S. Tang, J. Doorbar, Z. M. Zheng, Serine/arginine-rich splicing factor 3 and heterogeneous nuclear ribonucleoprotein A1 regulate alternative RNA splicing and gene expression of human papillomavirus 18 through two functionally distinguishable cis elements. J. Virol. **90**, 9138–9152 (2016). 10.1128/JVI.00965-16 PMC5044842 27489271

[7] M. Ajiro, R. Jia, Y. Yang, J. Zhu, Z. M. Zheng, A genome landscape of SRSF3-regulated splicing events and gene expression in human osteosarcoma U2OS cells. Nucleic Acids Res. **44**, 1854–1870 (2016). 10.1093/nar/gkv1500 PMC4770227 26704980

[8] Z. Wang , Exon-centric regulation of pyruvate kinase M alternative splicing via mutually exclusive exons. J. Mol. Cell. Biol. **4**, 79–87 (2012). 10.1093/jmcb/mjr030 PMC3493165 22044881

[9] R. Jia , Control of the papillomavirus early-to-late switch by differentially expressed SRp20. J. Virol. **83**, 167–180 (2009). 10.1128/JVI.01719-08 PMC2612334 18945760

[10] V. Majerciak, M. Lu, X. Li, Z. M. Zheng, Attenuation of the suppressive activity of cellular splicing factor SRSF3 by Kaposi sarcoma-associated herpesvirus ORF57 protein is required for RNA splicing. RNA **20**, 1747–1758 (2014). 10.1261/rna.045500.114 PMC4201827 25234929

[11] H. Lou, K. M. Neugebauer, R. F. Gagel, S. M. Berget, Regulation of alternative polyadenylation by U1 snRNPs and SRp20. Mol. Cell. Biol. **18**, 4977–4985 (1998). 10.1128/mcb.18.9.4977 PMC109082 9710581

[12] N. L. Maciolek, M. T. McNally, Serine/arginine-rich proteins contribute to negative regulator of splicing element-stimulated polyadenylation in rous sarcoma virus. J. Virol. **81**, 11208–11217 (2007). 10.1128/JVI.00919-07 PMC2045511 17670832

[13] Y. Huang, J. A. Steitz, Splicing factors SRp20 and 9G8 promote the nucleocytoplasmic export of mRNA. Mol. Cell **7**, 899–905 (2001). 10.1016/s1097-2765(01)00233-7 11336712

[14] Y. Huang, R. Gattoni, J. Stévenin, J. A. Steitz, SR splicing factors serve as adapter proteins for TAP-dependent mRNA export. Mol. Cell **11**, 837–843 (2003). 10.1016/s1097-2765(03)00089-3 12667464

[15] V. C. Auyeung, I. Ulitsky, S. E. McGeary, D. P. Bartel, Beyond secondary structure: Primary-sequence determinants license pri-miRNA hairpins for processing. Cell **152**, 844–858 (2013). 10.1016/j.cell.2013.01.031 PMC3707628 23415231

[16] K. Kim, T. D. Nguyen, S. Li, T. A. Nguyen, SRSF3 recruits DROSHA to the basal junction of primary microRNAs. RNA **24**, 892–898 (2018). 10.1261/rna.065862.118 PMC6004053 29615481

[17] K. D. Fitzgerald, B. L. Semler, Re-localization of cellular protein SRp20 during poliovirus infection: Bridging a viral IRES to the host cell translation apparatus. PLoS Pathog. **7**, e1002127 (2011). 10.1371/journal.ppat.1002127 PMC3136463 21779168

[18] J. Kim , Splicing factor SRSF3 represses the translation of programmed cell death 4 mRNA by associating with the 5’-UTR region. Cell Death Differ. **21**, 481–490 (2014). 10.1038/cdd.2013.171 PMC3921596 24292556

[19] H. Jumaa, G. Wei, P. J. Nielsen, Blastocyst formation is blocked in mouse embryos lacking the splicing factor SRp20. Curr. Biol. **9**, 899–902 (1999). 10.1016/s0960-9822(99)80394-7 10469594

[20] R. Jia, C. Li, J. P. McCoy, C. X. Deng, Z. M. Zheng, SRp20 is a proto-oncogene critical for cell proliferation and tumor induction and maintenance. Int. J. Biol. Sci. **6**, 806–826 (2010). 10.7150/ijbs.6.806 PMC3005347 21179588

[21] Y. Tang , Downregulation of splicing factor SRSF3 induces p53β, an alternatively spliced isoform of p53 that promotes cellular senescence. Oncogene **32**, 2792–2798 (2013). 10.1038/onc.2012.288 PMC6503963 22777358

[22] M. L. Ankö, L. Morales, I. Henry, A. Beyer, K. M. Neugebauer, Global analysis reveals SRp20- and SRp75-specific mRNPs in cycling and neural cells. Nat. Struct. Mol. Biol. **17**, 962–970 (2010). 10.1038/nsmb.1862 20639886

[23] R. Karni , The gene encoding the splicing factor SF2/ASF is a proto-oncogene. Nat. Struct. Mol. Biol. **14**, 185–193 (2007). 10.1038/nsmb1209 PMC4595851 17310252

[24] O. Anczuków , The splicing factor SRSF1 regulates apoptosis and proliferation to promote mammary epithelial cell transformation. Nat. Struct. Mol. Biol. **19**, 220–228 (2012). 10.1038/nsmb.2207 PMC3272117 22245967

[25] O. Anczuków , SRSF1-regulated alternative splicing in breast cancer. Mol. Cell **60**, 105–117 (2015). 10.1016/j.molcel.2015.09.005 PMC4597910 26431027

[26] J. X. Du , Splicing factor SRSF1 promotes breast cancer progression via oncogenic splice switching of PTPMT1. J. Exp. Clin. Cancer Res. **40**, 171 (2021). 10.1186/s13046-021-01978-8 PMC8122567 33992102

[27] E. R. Malone, M. Oliva, P. J. B. Sabatini, T. L. Stockley, L. L. Siu, Molecular profiling for precision cancer therapies. Genome Med. **12**, 8 (2020). 10.1186/s13073-019-0703-1 PMC6961404 31937368

[28] G. J. Goodall, V. O. Wickramasinghe, RNA in cancer. Nat. Rev. Cancer **21**, 22–36 (2021). 10.1038/s41568-020-00306-0 33082563

[29] H. Fink , Global and regional cancer burden attributable to modifiable risk factors to inform prevention. Nat. Med. **32**, 1306–1315 (2026). 10.1038/s41591-026-04219-7 41634393

[30] K. Kurokawa , Downregulation of serine/arginine-rich splicing factor 3 induces G1 cell cycle arrest and apoptosis in colon cancer cells. Oncogene **33**, 1407–1417 (2014). 10.1038/onc.2013.86 23503458

[31] R. Jia , HnRNP L is important for the expression of oncogene SRSF3 and oncogenic potential of oral squamous cell carcinoma cells. Sci. Rep. **6**, 35976 (2016). 10.1038/srep35976 PMC5093901 27808105

[32] F. Bray , Global cancer statistics 2022: GLOBOCAN estimates of incidence and mortality worldwide for 36 cancers in 185 countries. CA Cancer J. Clin. **74**, 229–263 (2024). 10.3322/caac.21834 38572751

[33] I. Shimizu , Suppressive effect of oestradiol on chemical hepatocarcinogenesis in rats. Gut **42**, 112–119 (1998). 10.1136/gut.42.1.112 PMC1726966 9505896

[34] W. E. Naugler , Gender disparity in liver cancer due to sex differences in MyD88-dependent IL-6 production. Science **317**, 121–124 (2007). 10.1126/science.1140485 17615358

[35] Z. Li, G. Tuteja, J. Schug, K. H. Kaestner, Foxa1 and Foxa2 are essential for sexual dimorphism in liver cancer. Cell **148**, 72–83 (2012). 10.1016/j.cell.2011.11.026 PMC3266536 22265403

[36] S. Sen, H. Jumaa, N. J. Webster, Splicing factor SRSF3 is crucial for hepatocyte differentiation and metabolic function. Nat. Commun. **4**, 1336 (2013). 10.1038/ncomms2342 PMC4314219 23299886

[37] D. Kumar , Degradation of splicing factor SRSF3 contributes to progressive liver disease. J. Clin. Invest. **129**, 4477–4491 (2019). 10.1172/JCI127374 PMC6763247 31393851

[38] S. Sen, M. Langiewicz, H. Jumaa, N. J. Webster, Deletion of serine/arginine-rich splicing factor 3 in hepatocytes predisposes to hepatocellular carcinoma in mice. Hepatology **61**, 171–183 (2015). 10.1002/hep.27380 PMC4280350 25132062

[39] S. Ali, R. C. Coombes, Endocrine-responsive breast cancer and strategies for combating resistance. Nat. Rev. Cancer **2**, 101–112 (2002). 10.1038/nrc721 12635173

[40] Y. Xu , ERα is an RNA-binding protein sustaining tumor cell survival and drug resistance. Cell **184**, 5215–5229.e17 (2021). 10.1016/j.cell.2021.08.036 PMC8547373 34559986

[41] H. Ke , Loss of TDP43 inhibits progression of triple-negative breast cancer in coordination with SRSF3. Proc. Natl. Acad. Sci. U.S.A. **115**, E3426–E3435 (2018). 10.1073/pnas.1714573115 PMC5899436 29581274

[42] L. Guo , TDP43 promotes stemness of breast cancer stem cells through CD44 variant splicing isoforms. Cell Death Dis. **13**, 428 (2022). 10.1038/s41419-022-04867-w PMC9065105 35504883

[43] L. Yu, V. Majerciak, R. Jia, Z. M. Zheng, Revisiting and corrections to the annotated SRSF3 (SRp20) gene structure and RefSeq sequences from the human and mouse genomes. Cell Insight **2**, 100089 (2023). 10.1016/j.cellin.2023.100089 PMC10134197 37193066

[44] W. J. Muller, E. Sinn, P. K. Pattengale, R. Wallace, P. Leder, Single-step induction of mammary adenocarcinoma in transgenic mice bearing the activated c-neu oncogene. Cell **54**, 105–115 (1988). 10.1016/0092-8674(88)90184-5 2898299

[45] H. Jumaa, J. L. Guénet, P. J. Nielsen, Regulated expression and RNA processing of transcripts from the Srp20 splicing factor gene during the cell cycle. Mol. Cell. Biol. **17**, 3116–3124 (1997). 10.1128/mcb.17.6.3116 PMC232164 9154810

[46] H. Jumaa, P. J. Nielsen, The splicing factor SRp20 modifies splicing of its own mRNA and ASF/SF2 antagonizes this regulation. EMBO J. **16**, 5077–5085 (1997). 10.1093/emboj/16.16.5077 PMC1170142 9305649

[47] X. Y. Tan , WNT2-SOX4 positive feedback loop promotes chemoresistance and tumorigenesis by inducing stem-cell like properties in gastric cancer. Oncogene **42**, 3062–3074 (2023). 10.1038/s41388-023-02816-1 37634009

[48] J. Liu , SOX4 maintains the stemness of cancer cells via transcriptionally enhancing HDAC1 revealed by comparative proteomics study. Cell Biosci. **11**, 23 (2021). 10.1186/s13578-021-00539-y PMC7821488 33482915

[49] M. Hagiwara , MUC1-C activates the BAF (mSWI/SNF) complex in prostate cancer stem cells. Cancer Res. **81**, 1111–1122 (2021). 10.1158/0008-5472.CAN-20-2588 PMC8026569 33323379

[50] C. L. Gilchrist , TRPV4-mediated calcium signaling in mesenchymal stem cells regulates aligned collagen matrix formation and vinculin tension. Proc. Natl. Acad. Sci. U.S.A. **116**, 1992–1997 (2019). 10.1073/pnas.1811095116 PMC6369759 30674675

[51] T. Sato, F. Okumura, T. Ariga, S. Hatakeyama, TRIM6 interacts with Myc and maintains the pluripotency of mouse embryonic stem cells. J. Cell Sci. **125**, 1544–1555 (2012). 10.1242/jcs.095273 22328504

[52] A. M. Jaworska, N. A. Wlodarczyk, A. Mackiewicz, P. Czerwinska, The role of TRIM family proteins in the regulation of cancer stem cell self-renewal. Stem Cells **38**, 165–173 (2020). 10.1002/stem.3109 PMC7027504 31664748

[53] S. Shen , MATS: A Bayesian framework for flexible detection of differential alternative splicing from RNA-Seq data. Nucleic Acids Res. **40**, e61 (2012). 10.1093/nar/gkr1291 PMC3333886 22266656

[54] G. Song , E3 ubiquitin ligase RNF128 promotes innate antiviral immunity through K63-linked ubiquitination of TBK1. Nat. Immunol. **17**, 1342–1351 (2016). 10.1038/ni.3588 27776110

[55] D. Ray , Isoforms of RNF128 regulate the stability of mutant P53 in Barrett’s esophageal cells. Gastroenterology **158**, 583–597.e1 (2020). 10.1053/j.gastro.2019.10.040 PMC7429981 31715145

[56] Y. Zhang , Characterizing isoform switching events in esophageal adenocarcinoma. Mol. Ther. Nucleic Acids **29**, 749–768 (2022). 10.1016/j.omtn.2022.08.018 PMC9437810 36090744

[57] H. Yang , MFSD4A inhibits the malignant progression of nasopharyngeal carcinoma by targeting EPHA2. Cell Death Dis. **13**, 332 (2022). 10.1038/s41419-022-04793-x PMC9001682 35410462

[58] M. Kanda , Metastatic pathway-specific transcriptome analysis identifies MFSD4 as a putative tumor suppressor and biomarker for hepatic metastasis in patients with gastric cancer. Oncotarget **7**, 13667–13679 (2016). 10.18632/oncotarget.7269 PMC4924669 26872374

[59] M. Ito , TIP30 deficiency increases susceptibility to tumorigenesis. Cancer Res. **63**, 8763–8767 (2003). 14695192

[60] J. Pecha , Deletion of Tip30 leads to rapid immortalization of murine mammary epithelial cells and ductal hyperplasia in the mammary gland. Oncogene **26**, 7423–7431 (2007). 10.1038/sj.onc.1210548 17533366

[61] A. Li , TIP30 loss enhances cytoplasmic and nuclear EGFR signaling and promotes lung adenocarcinogenesis in mice. Oncogene **32**, 2273–2281, 2281e.1–2281e.12 (2013). 10.1038/onc.2012.253 PMC3460142 22733137

[62] S. Lyu , High expression of eIF4A2 is associated with a poor prognosis in esophageal squamous cell carcinoma. Oncol. Lett. **20**, 177 (2020). 10.3892/ol.2020.12038 PMC7471732 32934744

[63] C. Xue, X. Gu, G. Li, Z. Bao, L. Li, Expression and functional roles of eukaryotic initiation factor 4A family proteins in human cancers. Front. Cell Dev. Biol. **9**, 711965 (2021). 10.3389/fcell.2021.711965 PMC8640450 34869305

[64] S. Lykke-Andersen, T. H. Jensen, Nonsense-mediated mRNA decay: An intricate machinery that shapes transcriptomes. Nat. Rev. Mol. Cell Biol. **16**, 665–677 (2015). 10.1038/nrm4063 26397022

[65] M. Huth , NMD is required for timely cell fate transitions by fine-tuning gene expression and regulating translation. Genes Dev. **36**, 348–367 (2022). 10.1101/gad.347690.120 PMC8973849 35241478

[66] M. Kalra, J. Mayes, S. Assefa, A. K. Kaul, R. Kaul, Role of sex steroid receptors in pathobiology of hepatocellular carcinoma. World J. Gastroenterol. **14**, 5945–5961 (2008). 10.3748/wjg.14.5945 PMC2760195 18932272

[67] T. Nakatani, G. Roy, N. Fujimoto, T. Asahara, A. Ito, Sex hormone dependency of diethylnitrosamine-induced liver tumors in mice and chemoprevention by leuprorelin. Jpn. J. Cancer Res. **92**, 249–256 (2001). 10.1111/j.1349-7006.2001.tb01089.x PMC5926710 11267934

[68] C. M. Shachaf , MYC inactivation uncovers pluripotent differentiation and tumour dormancy in hepatocellular cancer. Nature **431**, 1112–1117 (2004). 10.1038/nature03043 15475948

[69] S. Y. Jiang, R. Y. Shyu, M. Y. Yeh, V. C. Jordan, Tamoxifen inhibits hepatoma cell growth through an estrogen receptor independent mechanism. J. Hepatol. **23**, 712–719 (1995). 10.1016/0168-8278(95)80038-7 8750171

[70] Y. Zhao, Z. Li, Interplay of estrogen receptors and FOXA factors in the liver cancer. Mol. Cell. Endocrinol. **418**, 334–339 (2015). 10.1016/j.mce.2015.01.043 PMC4524798 25661537

[71] E. E. Swinstead , Steroid receptors reprogram FoxA1 occupancy through dynamic chromatin transitions. Cell **165**, 593–605 (2016). 10.1016/j.cell.2016.02.067 PMC4842147 27062924

[72] B. R. Zhou , Structural insights into the cooperative nucleosome recognition and chromatin opening by FOXA1 and GATA4. Mol. Cell. **84**, 3061–3079.e10 (2024). 10.1016/j.molcel.2024.07.016 PMC11344660 39121853

[73] D. P. Aden, A. Fogel, S. Plotkin, I. Damjanov, B. B. Knowles, Controlled synthesis of HBsAg in a differentiated human liver carcinoma-derived cell line. Nature **282**, 615–616 (1979). 10.1038/282615a0 233137

[74] V. A. Arzumanian, O. I. Kiseleva, E. V. Poverennaya, The curious case of the HepG2 cell line: 40 years of expertise. Int. J. Mol. Sci. **22**, 13135 (2021). 10.3390/ijms222313135 PMC8658661 34884942

[75] Y. Li , Loss of splicing factor SRSF3 impairs lipophagy through ubiquitination and degradation of Syntaxin17 in hepatocytes. J. Lipid Res. **64**, 100342 (2023). 10.1016/j.jlr.2023.100342 PMC10020108 36764525

[76] A. Nardone , A distinct chromatin state drives therapeutic resistance in invasive lobular breast cancer. Cancer Res. **82**, 3673–3686 (2022). 10.1158/0008-5472.CAN-21-3186 PMC9588703 35950920

[77] R. Xiao , Pervasive chromatin-RNA binding protein interactions enable RNA-based regulation of transcription. Cell **178**, 107–121.e18 (2019). 10.1016/j.cell.2019.06.001 PMC6760001 31251911

[78] R. Derynck, R. J. Akhurst, A. Balmain, TGF-beta signaling in tumor suppression and cancer progression. Nat. Genet. **29**, 117–129 (2001). 10.1038/ng1001-117 11586292

[79] L. Yang, H. L. Moses, Transforming growth factor beta: Tumor suppressor or promoter? Are host immune cells the answer? Cancer Res. **68**, 9107–9111 (2008). 10.1158/0008-5472.CAN-08-2556 PMC2741321 19010878

[80] N. D. Perkins, The diverse and complex roles of NF-κB subunits in cancer. Nat. Rev. Cancer **12**, 121–132 (2012). 10.1038/nrc3204 22257950

[81] M. Tesio, A. Trinquand, E. Macintyre, V. Asnafi, Oncogenic PTEN functions and models in T-cell malignancies. Oncogene **35**, 3887–3896 (2016). 10.1038/onc.2015.462 26616857

[82] S. Shojaee , PTEN opposes negative selection and enables oncogenic transformation of pre-B cells. Nat. Med. **22**, 379–387 (2016). 10.1038/nm.4062 PMC5178869 26974310

[83] P. Wimmer , PML isoforms IV and V contribute to adenovirus-mediated oncogenic transformation by functionally inhibiting the tumor-suppressor p53. Oncogene **35**, 69–82 (2016). 10.1038/onc.2015.63 25772236

[84] F. Renner, R. Moreno, M. L. Schmitz, SUMOylation-dependent localization of IKKepsilon in PML nuclear bodies is essential for protection against DNA-damage-triggered cell death. Mol. Cell **37**, 503–515 (2010). 10.1016/j.molcel.2010.01.018 20188669

[85] S. Y. Wu , Opposing functions of BRD4 isoforms in breast cancer. Mol. Cell **78**, 1114–1132.e10 (2020). 10.1016/j.molcel.2020.04.034 PMC7362310 32446320

[86] S. Tang, K. Yamanegi, Z. M. Zheng, Requirement of a 12-base-pair TATT-containing sequence and viral lytic DNA replication in activation of the Kaposi’s sarcoma-associated herpesvirus K8.1 late promoter. J. Virol. **78**, 2609–2614 (2004). 10.1128/JVI.78.5.2609-2614.2004 PMC369211 14963167

[87] D. Kumar , Hepatocyte deletion of IGF2 prevents DNA damage and tumor formation in hepatocellular carcinoma. Adv. Sci. (Weinh.) **9**, e2105120 (2022). 10.1002/advs.202105120 PMC9313545 35615981

[88] H. Wang , Alteration of splicing factors’ expression during liver disease progression: Impact on hepatocellular carcinoma outcome. Hepatol. Int. **13**, 454–467 (2019). 10.1007/s12072-019-09950-7 31140152

[89] C. J. Sherr, J. M. Roberts, Inhibitors of mammalian G1 cyclin-dependent kinases. Genes Dev. **9**, 1149–1163 (1995). 10.1101/gad.9.10.1149 7758941

[90] W. H. Liggett Jr., D. Sidransky, Role of the p16 tumor suppressor gene in cancer. J. Clin. Oncol. **16**, 1197–1206 (1998). 10.1200/JCO.1998.16.3.1197 9508208

[91] C. H. Chung , P16 protein expression and human papillomavirus status as prognostic biomarkers of nonoropharyngeal head and neck squamous cell carcinoma. J. Clin. Oncol. **32**, 3930–3938 (2014). 10.1200/JCO.2013.54.5228 PMC4251957 25267748

[92] C. Romagosa , p16(Ink4a) overexpression in cancer: A tumor suppressor gene associated with senescence and high-grade tumors. Oncogene **30**, 2087–2097 (2011). 10.1038/onc.2010.614 21297668

[93] X. Wang, C. Meyers, M. Guo, Z. M. Zheng, Upregulation of p18Ink4c expression by oncogenic HPV E6 via p53-miR-34a pathway. Int. J. Cancer **129**, 1362–1372 (2011). 10.1002/ijc.25800 PMC3086996 21128241

[94] D. A. Solomon , Identification of p18 INK4c as a tumor suppressor gene in glioblastoma multiforme. Cancer Res. **68**, 2564–2569 (2008). 10.1158/0008-5472.CAN-07-6388 PMC4394609 18381405

[95] T. Abbas, A. Dutta, p21 in cancer: Intricate networks and multiple activities. Nat. Rev. Cancer **9**, 400–414 (2009). 10.1038/nrc2657 PMC2722839 19440234

[96] H. Gautrey , SRSF3 and hnRNP H1 regulate a splicing hotspot of HER2 in breast cancer cells. RNA Biol. **12**, 1139–1151 (2015). 10.1080/15476286.2015.1076610 PMC4829299 26367347

[97] H. Proud , HER2∆16 directs luminal cell identity and estrogen receptor signaling in HER2+ breast cancer. Nat. Commun. **17**, 7532 (2026). 10.1038/s41467-026-74435-9 PMC13408682 42297801

[98] S. Shen, Y. Wang, C. Wang, Y. N. Wu, Y. Xing, SURVIV for survival analysis of mRNA isoform variation. Nat. Commun. **7**, 11548 (2016). 10.1038/ncomms11548 PMC4906168 27279334

[99] Y. Yang , Nuclear transport proteins: Structure, function, and disease relevance. Signal Transduct. Target. Ther. **8**, 425 (2023). 10.1038/s41392-023-01649-4 PMC10636164 37945593

[100] Y. Zhao, C. Xing, H. Peng, ALYREF (Aly/REF export factor): A potential biomarker for predicting cancer occurrence and therapeutic efficacy. Life Sci. **338**, 122372 (2024). 10.1016/j.lfs.2023.122372 38135116

[101] L. Yu, A. Lobanov, Z. M. Zheng, RNA-seq analysis of Srsf3 conditional knockout mouse breast tumor and liver tumor tissues. Gene Expression Omnibus. https://www.ncbi.nlm.nih.gov/geo/query/acc.cgi?acc=GSE276011. Deposited 29 August 2024.

[102] L. Yu, M. Ajiro, V. Majerciak, Z. M. Zheng, Y. He, SRSF3 plays a key role in liver cancer sex disparity by facilitating hepatocyte expression of ERα and FOXA1. Gene Expression Omnibus. https://www.ncbi.nlm.nih.gov/geo/query/acc.cgi?acc=GSE331053. Deposited 15 May 2026. 10.1073/pnas.2536317123 PMC13643318 42809395

