# Supplementary material for: SRSF3 plays a key role in liver cancer sex disparity by facilitating hepatocyte expression of ERα and FOXA
Source: Proc Natl Acad Sci U S A. 2026 Sep 29;123(40):e2536317123. doi: 10.1073/pnas.2536317123 (PMC13643318; doi:10.1073/pnas.2536317123)
Supplement: Supplementary file 1 [file pnas.2536317123.sapp.pdf]

Supplemental Information

Materials and Methods

Generation of transgenic mice

Srsf3^{tm1Pjln} mice (Srsf3$^{flox/flox}$ BALB/c) were provided from Hassan Jumaa (1). Albumin promoter-regulated Cre (Alb-Cre) expressing strain (C57BL/6) and MMTV promoter-regulated Cre (MMTV-Cre) expressing strain (FVB) were provided from Rui-Hong Wang and Chuxia Deng. MMTV promoter-regulated rat mutant c-neu strain, FVB-Tg (MMTV-Erbb2) NK1Mul/J, was purchased from the Jackson Laboratory (Bar Harbor, ME). Animal study was approved by the Institutional Animal Care and Use Committee of NCI-Frederick (protocol numbers 14-101, 17-101, 19-101, 22-101) and of NIH (HAMB-001).

All animals were maintained in accordance with the procedures outlined in the Guide for Care and Use of Laboratory Animals (National Research Council; 2011; National Academy Press; Washington, D.C.). NCI-Frederick animal facility is accredited by AAALAC International and follows the Public Health Service Policy for the Care and Use of Laboratory Animals.

Induction and monitoring tumor formation

Liver cancer formation was induced by intraperitoneal (i.p) injection of diethylnitrosamine (DEN) (25 mg/kg of body weight) at 15 days of mouse age (2). Breast cancer formation was induced by mouse mammary gland c-neu expression from MMTV promoter. Weekly surveys for liver and breast tumor formation were conducted by palpation by facility veterinarians in a blind manner for individual genotypes up to 78

weeks. Animals presenting tumors exceeding 20 mm in dimension, necrotic tumors, cutaneous ulceration, or multiple tumors collectively weighing more than 10% of the animal's body weight were euthanized via CO_2 asphyxiation. The mammary gland and liver tissues from sacrificed mice were collected for further pathology examinations. This procedure was conducted at humane endpoints in accordance with institutional Animal Care and Use Committee (ACUC) guidelines for tumor collection and biochemical analysis. Breast tissues and liver tissues at the age of 6 months and terminal life stage were collected for pathology analysis.

Total RNA sequencing and data processing

Total RNA was extracted separately from the Srsf3 WT and Srsf3 knockout (KO) liver and breast tumors using TriPure reagent (Roche, # 11667165001). The sequencing libraries were constructed following Illumina Stranded Total RNA protocol (Illumina, RS-122-2201). For liver RNA-seq, paired-end 125-bp read-length sequencing with a depth of 100 million reads per sample was performed on the Illumina platform according to the manufacturer's instructions. For breast RNA-seq, the paired-end read-length was 150-bp with a depth of 100 million reads per sample. The obtained sequence reads in fastq format were mapped to the mouse reference genome (mm10), and Limma-voom was used for quantile normalization and calculation of differentially expressed genes. Genes with significantly differential expression from Srsf3 KO and Srsf3 WT cancer ($p \leq 0.05$ with adjusted FDR $p \leq 0.05$), were considered and collected for further analyses. rMATs (3) software was used for differential alternative RNA splicing analysis. Instant Clue software (version 0.12.1) and ClustVis online heatmap software

(https://biit.cs.ut.ee/clustvis/) were used to generate the volcano plots and heatmaps, respectively.

NanoString nCounter gene expression validation

Four samples of total RNA per group from four different groups in each tumor model, including Srsf3 WT, Srsf3 homogenous knockout mice at 6th month tumor induction stage in both liver and breast group, were examined by NanoString nCounter gene expression analysis. Top upregulated or downregulated genes as well as genes not on the top list but have important functions identified by RNA-seq analysis in breast and liver cancer were chosen for validation (*SI Appendix,* Dataset S3). Probe sets for breast and liver tissues were custom designed and synthesized by NanoString company. Raw counts were extracted and normalized to three chosen housekeeping genes using NanoString nSolver 3.0 digital analyzer software.

Cell lines and siRNAs

Mouse liver cancer cell line Hepa1-6 cells and human liver cancer cell line Huh7 and HepG2 were obtained from T. Jake Liang lab (NIDDK/NIH). Mouse breast cancer cell line NF639 expressing c-neu oncogene, human breast cancer cell lines SKBR3 (HER2+) and MCF7 (ER+) cells were purchased from ATCC. SKBR3 cells were maintained in McCoy's 5A Medium (Thermo Fisher Scientific) with 10% fetal bovine serum (FBS, Cytiva). All the other cell lines were maintained in Dulbecco’s modified Eagle’s medium (DMEM) (Thermo Fisher Scientific) with 10% FBS at 37°C under a 5% CO_2 atmosphere.

Inducible SRp20 (SRSF3) expressing tumorigenic MEF/3T3 Tet-Off Cell Line (MEF-T7-SRp20) was established in our previous study (4) and is available from Kerafast (www.kerafast.com/item/1504/srp20-expressing-tumorigenic-mef3t3-tet-off-cell-line-mef-t7-srp20). Briefly, the MEF/3T3 Tet-Off cells (Clontech) were transfected with a T7-SRp20 expression RevTRE vector (pJR17) having T7-SRp20 under the control of tetracycline and stable selection was made under 200 µg/mL hygromycin and 1 µg/mL doxycycline conditions. To induce T7-SRSF3 expression, cells were maintained in regular DMEM supplemented with 200 µg/mL hygromycin B and 100 µg/mL G418 in the absence of doxycycline during cell passage (normally taking 3 passages to see inducible T7-SRSF3 expression).

For siRNA transfection, cells at 24 h of cell passage were transfected with 40 nM of siRNA, and total protein and RNA were extracted from the harvested cells 48 h after a single transfection or at 96 h after two sequential transfections at a 48 h interval. LipoJet In Vitro Transfection Kit (Ver. II) (SignaGen Laboratories, # SL100468) was used for Hepa1-6, Huh7, HepG2, and MCF7 transfections. Lipofectamin RNAiMAX Transfection Reagent (Thermo Fisher Scientific, # 13778075) was used for NF639 cell transfection. SKBR3 transfection kit (Altogen Biosystems, # 6905) were used for SKBR3 cell transfection. siRNAs from Dharmacon used in this study: mouse Srsf3 (# M-059214-01) and human SRSF3 (# M-030081-00). Non-targeting control siRNA (# D-001210-01) served as a negative control.

Cell proliferation assays

Cell proliferation was assessed using the Cell Counting Kit-8 (CCK-8) (Dojindo Molecular Technologies, #CK-04-11) according to the manufacturer's instructions. Relative cell viability was determined by measuring absorbance at 450 nm from three independent biological replicates.

Lipid droplets staining

For lipid droplet staining, cells were washed twice with PBS and fixed in 4% paraformaldehyde for 15 min at room temperature. Cells were subsequently stained with BODIPY 493/503 (Invitrogen, #D3922) for 30 min at room temperature. Oil Red O staining was performed using a commercial kit (Abcam, #ab150678), with cells incubated in Oil Red O solution for 3 h at room temperature according to the manufacturer's instructions.

RT-PCR and RT-qPCR

For reverse transcription-coupled polymerase chain reaction (RT-PCR), 1 μg of DNase-treated total RNA was reverse transcribed with MuLV RTase in the presence of random hexamers (Thermo Fisher Scientific), and PCR-amplified with AmpliTaq (Thermo Fisher Scientific). Primer sets used for RT-PCR were listed in *SI Appendix,* Dataset S6. The PCR products were separated on a 1.5% agarose gel by electrophoresis and imaged using ChemiDoc Touch System (Bio-Rad Laboratories, Inc, Hercules, CA). The PCR band intensity was analyzed and quantified by using Bio-Rad Image Lab Touch Software for PSI calculation. PSI, percent spliced-in of the alternative exon(s); small size band (E) (% exclusion = exclusion/sum of inclusion + exclusion);

large size band (I) (% inclusion = inclusion/sum of inclusion + exclusion). The comparison was made between the sum of both isoforms of inclusion and exclusion in WT and KO tissue after being normalized to Gapdh RNA in RT-PCR.

Quantitative RT-PCR (RT-qPCR) was conducted by TaqMan real-time PCR system (Thermo Fisher Scientific) for Erα (# Mm00433149_m1), Sox4 (# Mm00486320_s1), E2f1(# Mm00432939_m1),Trpv4 (# Mm00499025_m1), Trim6 (# Mm07305537_m1), Foxa1 (# Mm00484713_m1), Foxa2 (# Mm00839704_mH), Foxa3 (# Mm00484714_m1), Lifr (# Mm00442942_m1), Egfr (# Mm01187858_m1), Myc (# Mm00487804_m1), Htatip2 (#Mm00457476_m1) and Gapdh (# Mm99999915_g1). The threshold cycle (C_T) values of qRT-PCR data from 3 repeats were analyzed by the $2^{-\Delta\Delta CT}$ method and are presented as bar graphs with means ± standard deviations (SD).

5' rapid amplification of cDNA ends (RACE)

Total RNA was extracted using the TriPure reagent (Roche, # 11667165001). 5' RACE assays were performed using the SMARTer RACE cDNA Amplification Kit (Takara, #634858) according to the manufacturer’s instructions, with 1 µg of total RNA per reaction as the template. Primer sequences are listed in *SI Appendix* Dataset S6. RACE products were analyzed by agarose gel electrophoresis, purified from the gel, and subjected to Sanger sequencing.

Immunoblot, antibodies, tissue pathology, and immunohistochemistry (IHC)

For Western blot, SDS-PAGE was performed in 4-12% Bis-Tris NuPAGE gel (Thermo Fisher Scientific) and proteins were transferred to a PVDF membrane and then

immublotted with specific antibodies following blocking by 5% skim milk in 1 ×TBS (Tris-buffered saline). Human tissue microarrays of liver cancer and breast cancer were purchased from TissueArray.Com LLC (https://www.tissuearray.com) (Derwood, MD) and IHC was performed by standard HRP-DAB staining protocol for paraffin-embedded tissues fixed in 10% neutral buffered formalin with a counter staining by haematoxylin and eosin. Following antibodies were used for protein detection in Western blot and immunohistochemistry: anti-estrogen receptor α (ERα) rabbit polyclonal antibody (MC-20) (Santa Cruz Biotechnology, # sc-542), rabbit polyclonal anti-SRSF3 antibody (Abcam, # ab125124), anti-SRSF1 mouse monoclonal antibody (clone 96) (Thermo Fisher scientific, # 32-4500), anti-HER2/ErbB2 rabbit monoclonal antibody (Cell Signaling Technology, # 2165) for rat and mouse c-neu/Erbb2, anti-Eif4a2 rabbit polyclonal antibody (Abcam, # ab31218), anti-Foxa1 goat polyclonal antibody (Abcam, # ab5089), anti-capase-7 mouse monoclonal antibody (MBL international, #clone 4G2), anti-capase-3 rabbit polyclonal antibody (Calbiochem, #235412), anti-β-tubulin antibody (Sigma, # T5201), anti-β-actin mouse monoclonal antibody (AC-15) (Santa Cruz Biotechnology, # sc-69879), and anti-GAPDH rabbit monoclonal antibody (Cell Signaling Technology, # 2118) for GAPDH.

Plasma chemistry analysis

Plasma samples from DEN-induced Srsf3-WT or Srsf3-KO liver cancer mice at a year of age were subjected to serological assays to quantify plasma alanine transaminase (ALT), alkaline phosphatase, amylase activity, urea nitrogen and bilirubin

by VetScan VS2 system (ABAXIS), or plasma estradiol concentration by ELISA, which was performed by Ani Lytics, Inc. (Gaithersburg, MD).

Foxa1 promoter activity assays

The mouse Foxa1 promoter region spanning -1016 bp/+100 bp from annotated transcriptional start site (TSS) corresponding to ch12:57547127-57546012 region in mm10 genome was amplified using oVM620 (chr12: 57547127-57547107; B, KpnI; 5-GATAGGTACC/TAGCGTGGCAGATGCGTTCCA-3) and oVM616 primers (chr12: 57546012-57546031; F; HindIII; 5-ATGCCAAGCTT/TGCGAAGCGAGTGCAGTTGA-3) on total cell DNA extracted from mouse liver cancer Hepa1-6 cells. The Foxa1 promoter fragment obtained was cloned into pGL3-Basic (Promega) vector upstream of *Firefly* luciferase (FL) via *Kpn*I and *Hind*III sites and the constructed plasmid pVM174 was evaluated for Foxa1 promoter activity in Hepa1-6 cells. Briefly, Hepa1-6 cells (1.5 $x10^5$/well) were plated in 12-well plates to allow the cells to grow for 24 h. The cells were then transfected with a mixture of 250 ng of pVM174 and 25 ng of *Renilla* luciferase (RL) vector pRL-TS(5). The empty pGL3-basic vector was used as a negative control. The transfected cells were harvested 24 h after transfection, and the Foxa1 promoter activity represented by FL emitted light units was determined by Dual-Luciferase Reporter Assay System (Promega, E1910) and normalized to RL-emitted light units. To investigate the regulatory role of SRSF3 on Foxa1 promoter activity, Hepa1-6 cells (1.5 $x10^5$/well) were reverse transfected with 40 nm Srsf3-targeting siRNA (si-SRSF3) or negative control siRNA (si-Control) in a 12-well plate. The transfected cells at 24 h were transfected again with a mixture of 250 ng of Foxa1 promoter

luciferase reporter pVM174 and 25 ng of pRL-TS. The Foxa1 promoter activity was determined 24 h after transfection and normalized to cell proliferation assessed by Cell Counting Kit-8 (CCK-8, Dojindo, CK04-11). All experiments were independently repeated three times, with five replicates per condition.

Chromatin immuno-cleavage sequencing (ChIC-seq) and ChIP-seq analysis

SRSF3 ChIC-seq libraries were generated based on a modified small-cell-number ChIC-seq protocol (Keji Zhao Lab) (6) using pApG-DNase in place of protein A-MNase. Briefly, formaldehyde-fixed cells were prepared by incubating cultured cells with 1/15 volume of 16% methanol-free formaldehyde for 10 min at room temperature, followed by quenching with 1/10 volume of 1.25 M glycine for 5 min. Cells were washed twice with PBS and stored at −80 °C until use. For chromatin preparation, fixed cells were thawed on ice and permeabilized in RIPA buffer containing 0.2% SDS and 150 mM NaCl for 10 min on ice. Cells were washed once and resuspended in antibody-binding buffer (10 mM Tris-HCl pH 7.5, 150 mM NaCl, 0.1% Triton X-100). Anti-SRSF3 antibody (Abcam, #ab124125)–pApG-DNase complexes were assembled on ice for 30 min prior to use (0.4mL pApG-DNase was mixed with 1.5 µg primary antibody in 20 mL PBS). Approximately 200,000 cells were incubated with the antibody–pApG-DNase complexes in cold room for 1 h with gentle rotation. Following binding, samples were washed 3 times using either high-salt wash buffer (10 mM Tris-HCl pH 7.5, 300 mM NaCl, 1% Triton X-100) or Antibody binding buffer. Chromatin digestion was initiated by resuspending samples in reaction buffer containing 20 mM Tris-HCl pH 7.4, 10 mM NaCl, 2 mM $CaCl_2$, and 0.1% Triton X-100, followed by incubation at 37 °C for 3 min.

Reactions were terminated with stop buffer containing 20 mM Tris-HCl pH 8.0, 10 mM EGTA, 20 mM NaCl, and 0.2% SDS. Proteinase K was added and samples were incubated at 65 °C for reverse crosslinking and protein digestion overnight. DNA was purified using MinElute columns (Qiagen). Purified DNA fragments were subjected to NEBNext® DNA library prep kits according to the manufacture protocol. Libraries were amplified using Phusion High-Fidelity PCR Master Mix with indexed primers. PCR cycle numbers were adjusted according to cell numbers. Amplified libraries were size-selected (140–350 bp) by agarose gel electrophoresis, purified, and quantified using a Qubit dsDNA HS assay prior to sequencing. The libraries were sequenced by Nova-X and the sequence reads obtained were initially aligned to the reference genome(hg38) using Bowtie2 with default parameters and finally to the reference genome (hg19) to correlate with the published GSM3393528 (7). To retain uniquely mapped reads, PCR duplicates were removed and low-quality reads were filtered out. Genome-wide coverage tracks were generated using normalized read counts (CPM).

SRSF3 ChIP-seq with 2 replicated experiments in HepG2 cells was performed using anti-SRSF3 antibody (MBL Cat# RN080PW). ChIP-seq reads were aligned to the GRCh37 human genome using BWA. PCR duplicates and reads of low quality were filtered out. Signal tracks were generated with MACS2 and bigWig applications from UCSC genome browser. The data were deposited at NCBI GEO Series GSE120104 Sample GSM3393528 (7). The reads distribution was visualized in the Integrated Genome Browser (IGV, Broad Institute).

Statistics

Log-rank test and Kaplan-Meier plot were conducted using SigmaPlot (Systat Software Inc.). IHC images were analyzed by Aperio ImageScope (Leica Biosystems). The Fisher's exact test was used to compare the breast and liver tumor formation rates between Srsf3 WT and KO mice at 6 months. Gene expression levels across different groups, assessed through RT-qPCR, were evaluated using Student's *t*-test. For the cell proliferation assay, statistical significance was determined by two-way ANOVA with Bonferroni correction using data from three independent experiments. Biological differences with $p < 0.05$ were considered statistically significant.

References

1. H. Jumaa, G. Wei, P. J. Nielsen, Blastocyst formation is blocked in mouse embryos lacking the splicing factor SRp20. *Curr Biol* **9**, 899–902 (1999).
2. W. E. Naugler *et al.*, Gender disparity in liver cancer due to sex differences in MyD88-dependent IL-6 production. *Science* **317**, 121–124 (2007).
3. S. Shen *et al.*, MATS: a Bayesian framework for flexible detection of differential alternative splicing from RNA-Seq data. *Nucleic Acids Res* **40**, e61 (2012).
4. R. Jia, C. Li, J. P. McCoy, C. X. Deng, Z. M. Zheng, SRp20 is a proto-oncogene critical for cell proliferation and tumor induction and maintenance. *Int J Biol Sci* **6**, 806–826 (2010).
5. S. Tang, K. Yamanegi, Z. M. Zheng, Requirement of a 12-base-pair TATT-containing sequence and viral lytic DNA replication in activation of the Kaposi's sarcoma-associated herpesvirus K8.1 late promoter. *J Virol* **78**, 2609–2614 (2004).
6. W. L. Ku *et al.*, Single-cell chromatin immunocleavage sequencing (scChIC-seq) to profile histone modification. *Nat Methods* **16**, 323–325 (2019).
7. R. Xiao *et al.*, Pervasive Chromatin-RNA Binding Protein Interactions Enable RNA-Based Regulation of Transcription. *Cell* **178**, 107–121.e118 (2019).
8. L. Yu, V. Majerciak, R. Jia, Z. M. Zheng, Revisiting and corrections to the annotated SRSF3 (SRp20) gene structure and RefSeq sequences from the human and mouse genomes. *Cell Insight* **2**, 100089 (2023).
9. H. Jumaa, G. Wei, P. J. Nielsen, Blastocyst formation is blocked in mouse embryos lacking the splicing factor SRp20. *Curr Biol* **9**, 899–902 (1999).
10. L. Porras, H. Ismail, S. Mader, Positive Regulation of Estrogen Receptor Alpha in Breast Tumorigenesis. *Cells* **10,** 2966 (2021).
11. A. Nardone et al., A Distinct Chromatin State Drives Therapeutic Resistance in Invasive Lobular Breast Cancer. *Cancer Res* **82**, 3673–3686 (2022).

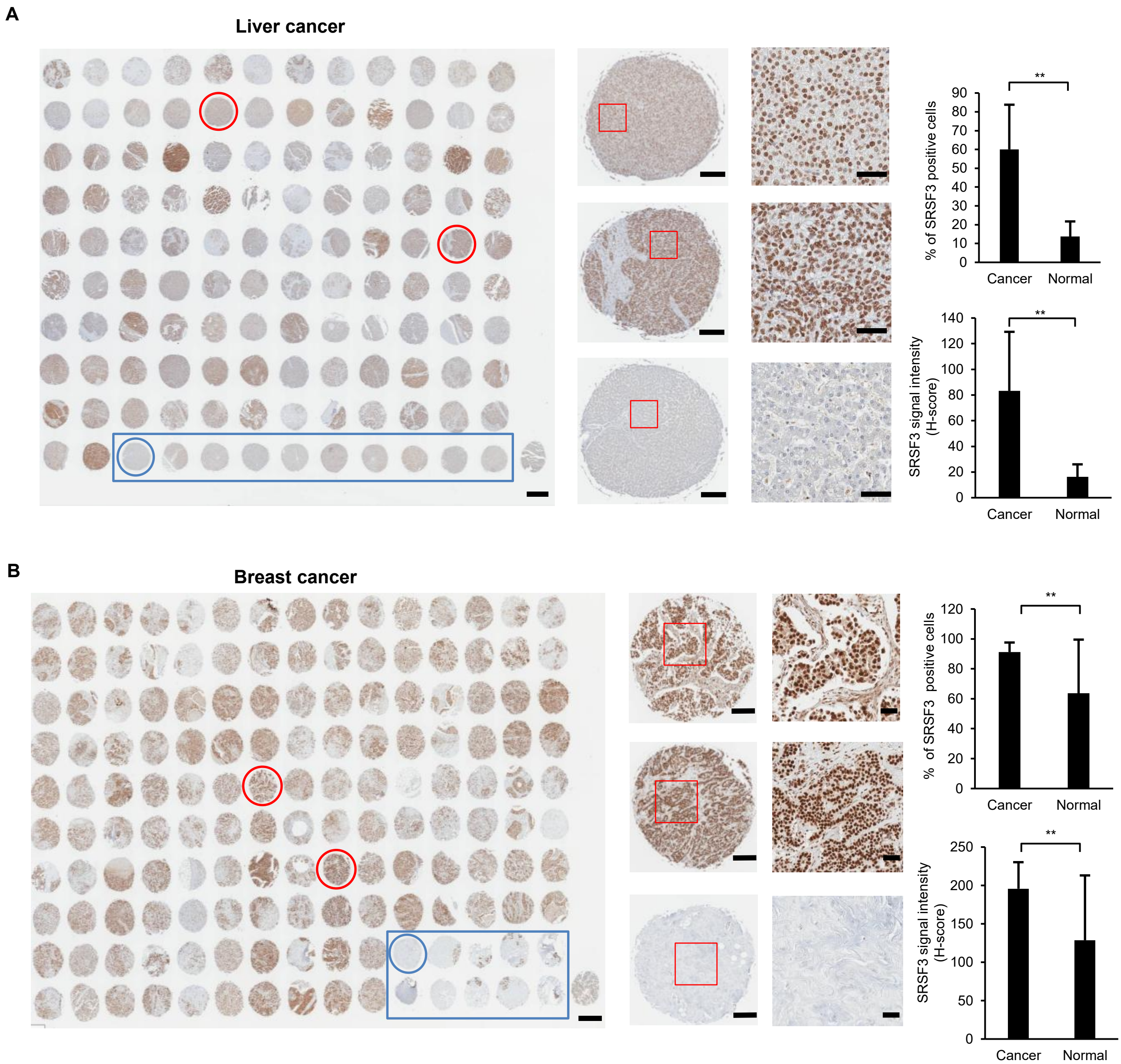

Fig. S1. SRSF3 overexpressed in both liver cancer and breast cancer tissues. (A) SRSF3 immunohistology staining of 110 unpaired, single human liver cancer tissues and 10 normal liver tissues (tissue array information could be found in https://www.tissuearray.com/tissue-arrays/Liver/BC03119b). (B) SRSF3 immunohistology staining of 70 paired human breast cancer tissues and 5 paired normal tissues (tissue array information could be found in https://www.tissuearray.com/BR1504b). For both panel A and panel B, Scale bars from left to right, 1mm, 200μm, 50μm. Normal tissues were highlighted in blue rectangle. SRSF3 signal was quantified by HALO software (https://indicalab.com/halo/). Statistical comparisons were computed by unpaired, two-tailed Student's t test. ** p<0.01.

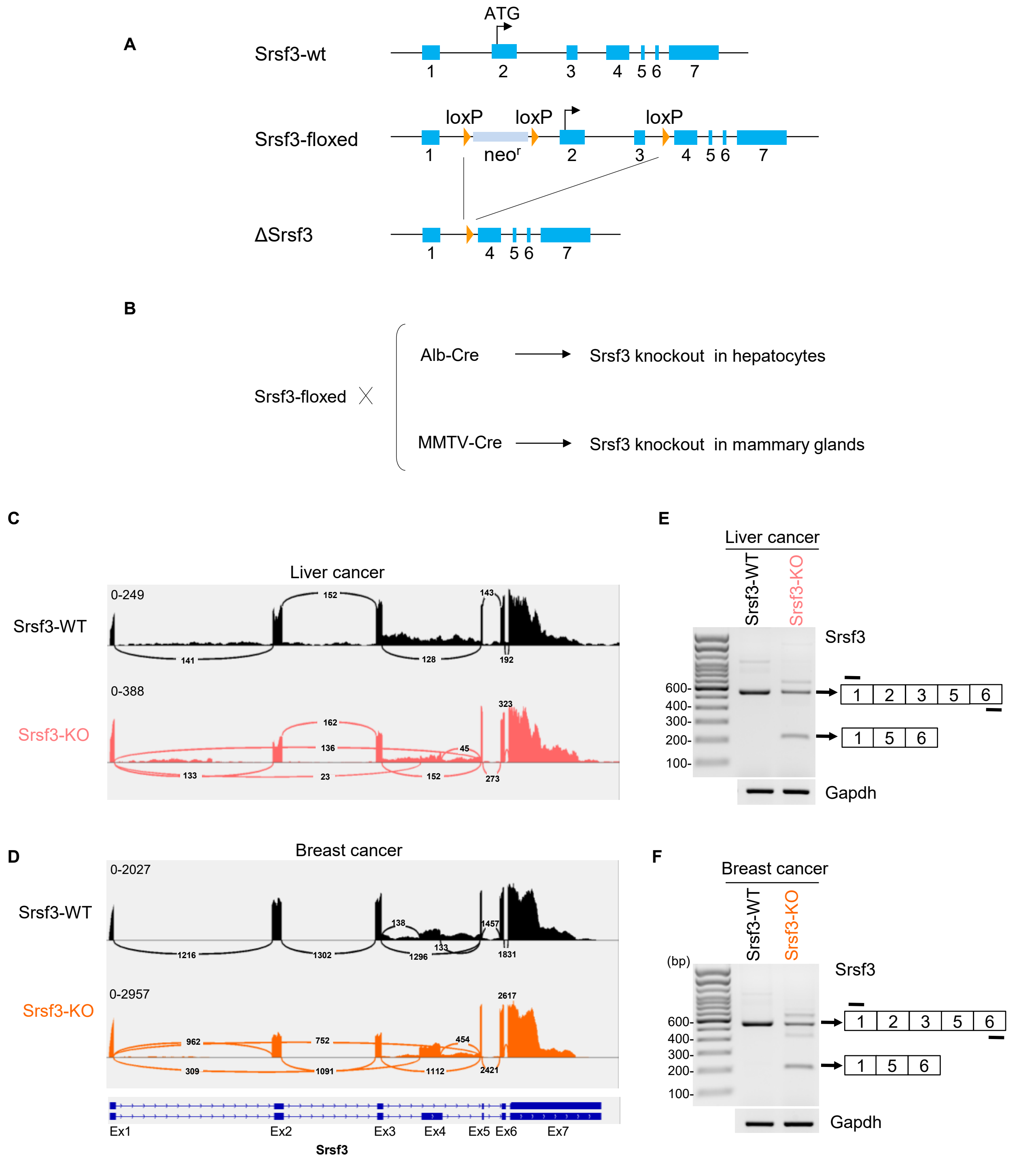

Fig. S2. Strategies implemented for tissue-specific conditional knockout (KO) of mouse Srsf3 in liver and breast tissues. (A) Diagrams of Srsf3 gene structure and KO strategy by Cre-loxP technology. Srsf3 gene consists of seven exons with the start codon AUG in the second exon (8). In the Srsf3-floxed allele (Srsf3-floxed) as described (9), loxP sequences are inserted in the first and third introns, leading to excise the Srsf3 exon 2 and 3 from genomic DNA when Cre recombinase is expressed in cells to induce Srsf3 knockout due to loss of start codon (ΔSrsf3) in exon 2. Floxed Neo^R, neomycin-resistant gene, in the SRSF3 intron 1 under control by LoxP for cell selection. (B) Tissue-specific Srsf3 knockout is achieved by crossing Srsf3-floxed mice with Cre recombinase-expressing mice under an albumin promoter (Alb-Cre) or a murine mammary tumor virus promoter (MMTV-Cre), selectively active in hepatocytes or mammary gland tissues, respectively.(C, D) Sashimi plots showing the alternative splicing of Srsf3 gene in representative Srsf3 WT and KO liver cancer (C) and breast cancer tissues (D). The numbered arches represent splice junction reads detected from IGV. (E, F) RT-PCR validation for Srsf3 KO in liver cancer (E) and breast cancer tissues (F). Cre-LoxP recombination or KO of Srsf3 exons 2 + 3 mediated by tissue-specific Cre was confirmed by indicated primers from exon 1 and exon 6 (dash lines). Gapdh RNA served as a loading control.

A

Srsf3 KO (♀)

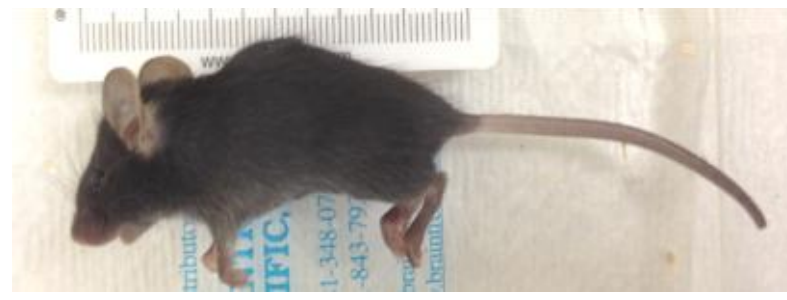

Srsf3 WT (♀)

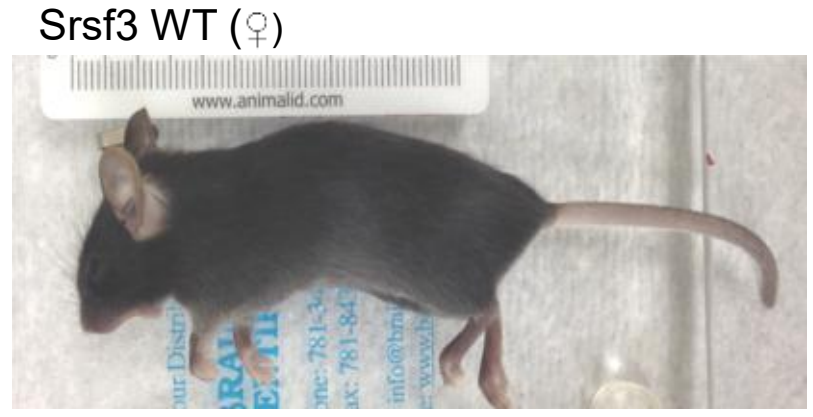

B

Liver cancer induction

Srsf3-WT ♂, ♀
Srsf3-hetKO ♂, ♀
Srsf3-KO ♂, ♀

× DEN (diethylnitrosamine) i.p. injection (25 mg/kg) at 15 days of age → Liver cancer observation (up to 18 months)

Breast cancer induction

Srsf3-WT ♀
Srsf3-hetKO ♀
Srsf3-KO ♀

× MMTV-Erbb2 (c-neu) (V664E mutant) → Breast cancer observation (up to 18 months)

C

Early death incidence of the mice after DEN injection

genotype/gender	Incidence		Death days after birth (mean ± SE)
Srsf3 WT, DEN ♂ (n=76)	0/76	(0%)	N/D
Srsf3 hetKO, DEN ♂ (n=17)	0/17	(0%)	N/D
Srsf3 KO, DEN ♂ (n=38)	9/38*	(23.7%)	31.8 ± 1.7
Srsf3 WT, DEN ♀ (n=92)	0/92	(0%)	N/D
Srsf3 hetKO, DEN ♀ (n=19)	0/19	(0%)	N/D
Srsf3 KO, DEN ♀ (n=54)	13/54*	(24.1%)	37.5 ± 6.5

Fig. S3. Summary of early phenotypes of liver Srsf3-KO mice. (A) Pictures represent typical liver Srsf3-KO and WT female mice at 2 weeks after birth. (B) Liver cancer induction in mice with liver-specific Srsf3 KO was performed by the intraperitoneal (i.p.) injection of diethylnitrosamine (DEN) at 25 mg/kg at 15 days of age. Mammary cancer induction was assessed by crossing Srsf3-WT, -hetKO (heterozygous knockout), -KO female mice with male mice carrying a homozygous V664E mutant of rat c-neu/Erbb2 of which expression was under control by a MMTV promoter (MMTV). Tumor formation was surveyed by palpation weekly up to 18 months of age.
(C) Early death incidence of Srsf3-WT, -hetKO, and -KO mice by days after one dose DEN injection on day 15 of mouse age. *Some of the survived mice with DEN injection were euthanized for serology and pathology examination in the middle term of the study.

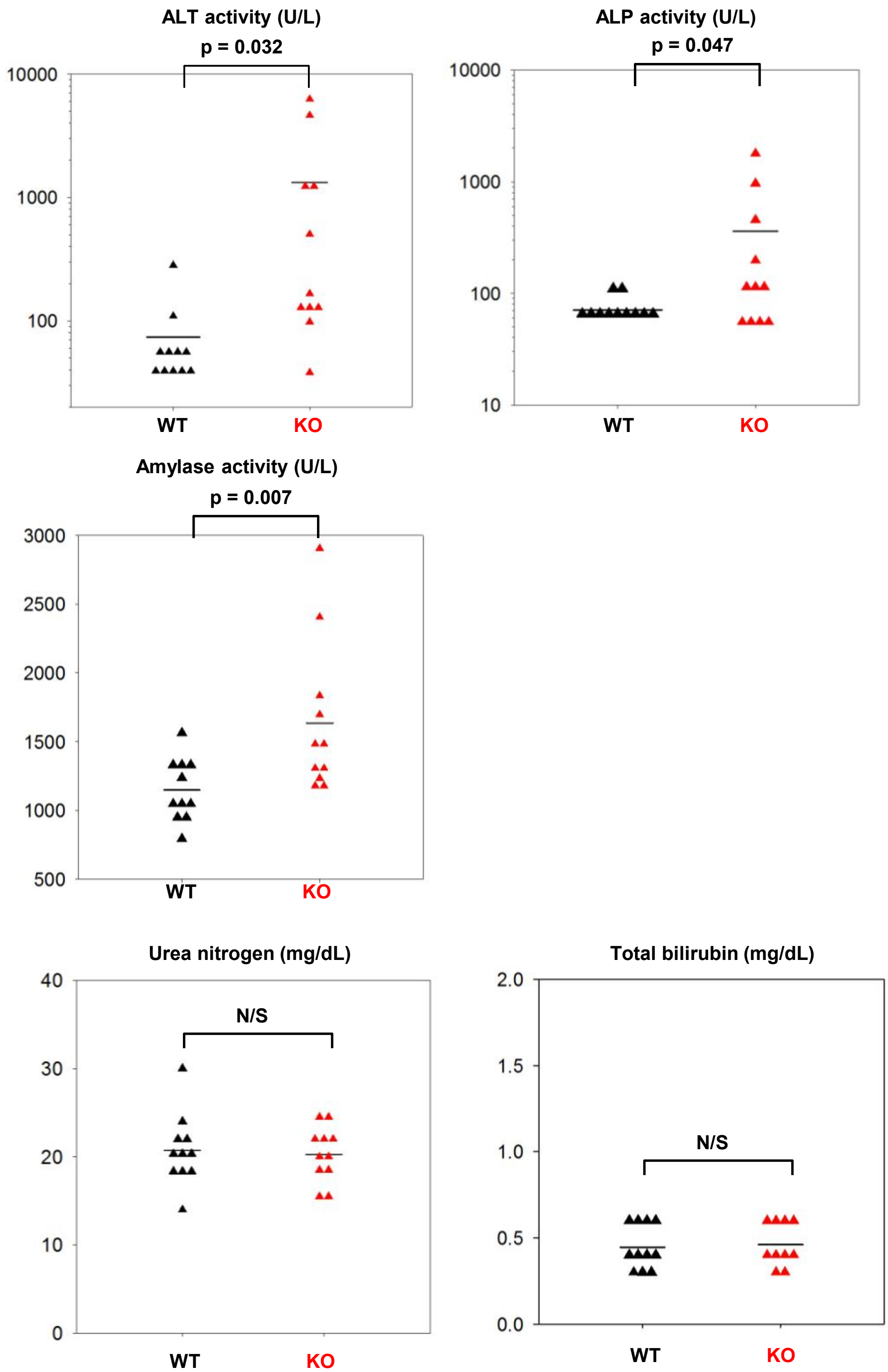

Fig. S4. Srsf3 KO affects mouse liver function changes in the 1-year-old animals. Blood samples were collected from DEN-injected Srsf3 WT and liver Srsf3 KO mice at a year of mouse age and serum ALT, alkaline phosphatase, amylase activity, urea nitrogen and bilirubin were measured in a blood chemistry laboratory. A p-value was calculated by Student's t-test. N/S, not significant.

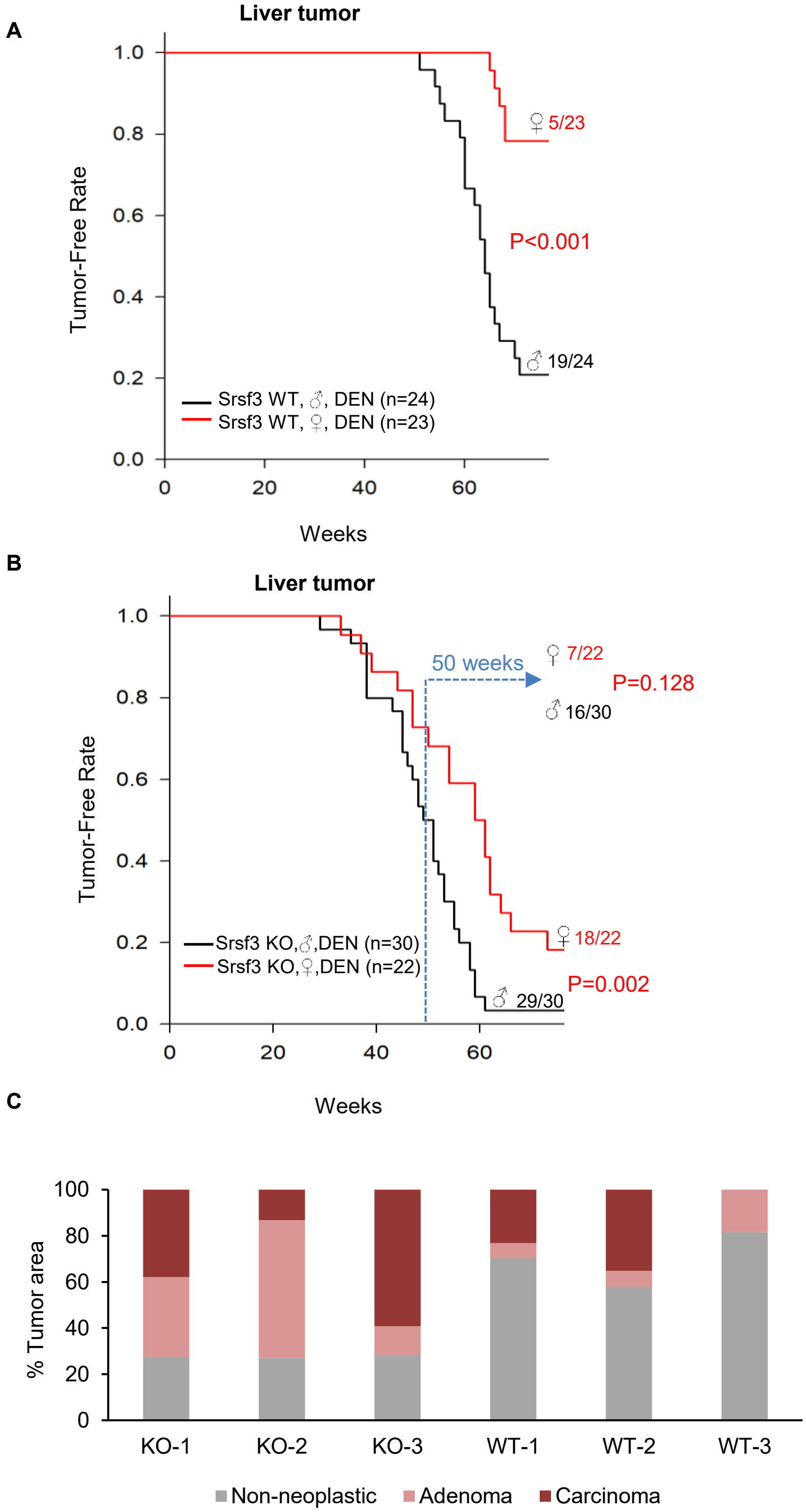

**Tumor area (adenoma+carcinoma)**	p-value
Srsf3 KO vs. Srsf3 WT	0.006

Fig. S5. Gender disparity in DEN-induced liver cancer incidence between male and female mice with or without liver Srsf3 KO. Kaplan-Meier plots were applied for the incidence of palpable liver tumors in Srsf3-WT (A) and Srsf3-KO mice (B) after DEN injection at day 15 of mouse age. N= number of animals in each group. P-values were determined by log-rank test between indicated groups. (C) Quantification of tumor area with pathology diagnosis in female Srsf3 KO and WT liver tumors on H&E-staining slides. A p-value was calculated by Student's t-test.

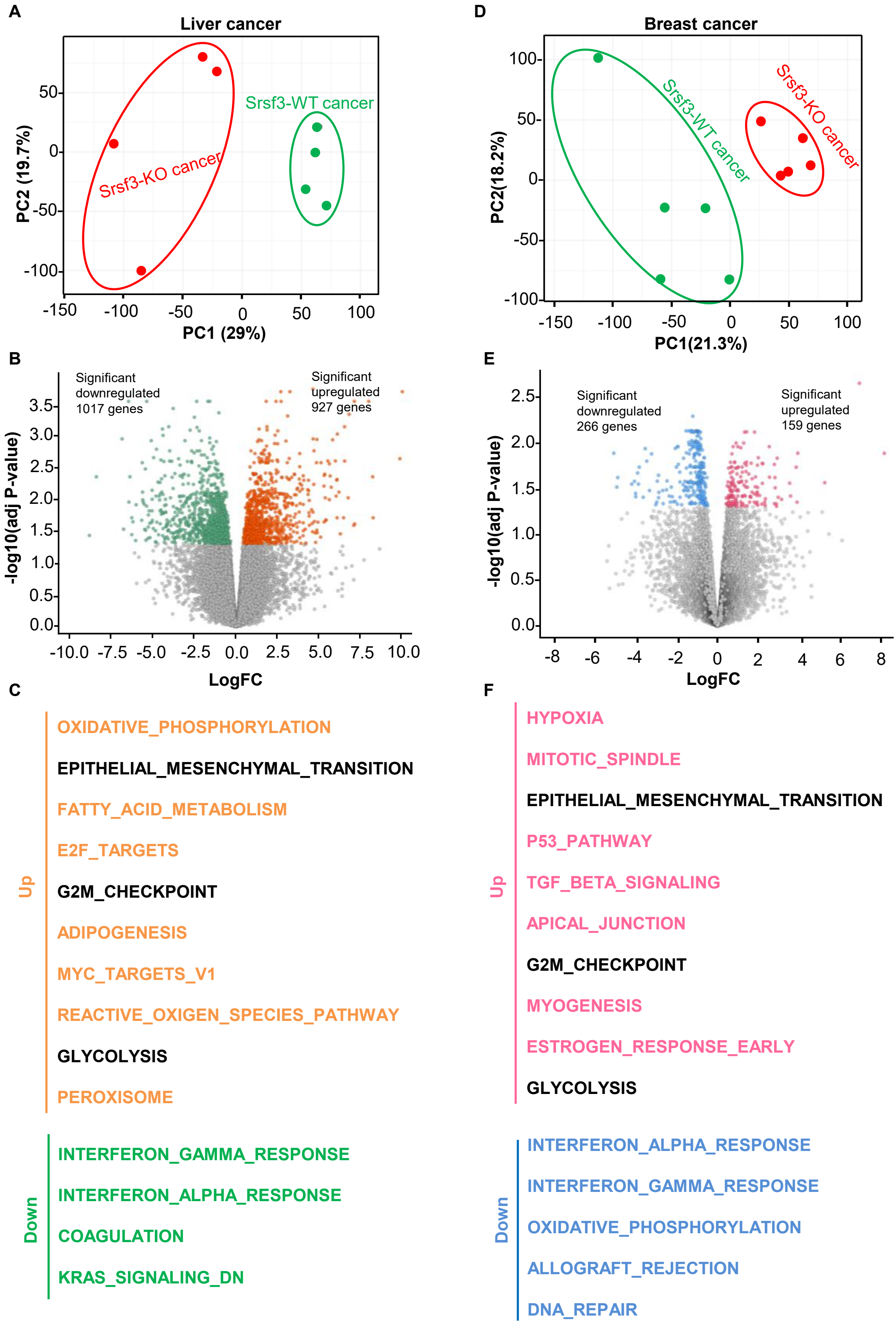

Fig. S6. RNA-seq analysis of Srsf3 associated genes in Srsf3-KO and Srsf3-WT liver and breast cancers. Principal component analysis (PCA) of Srsf3 WT (green) and Srsf3 KO (red) liver cancer (A) and breast cancer (D) by RNA-seq analysis. Four liver cancer tissues and five mammary gland cancer tissues were submitted to RNA-seq analysis in each group. (B and E) Volcano plot showing the down-regulated and upregulated genes (adjusted $p \leq 0.05$) in Srsf3 KO cancer compared to Srsf3 WT cancer. (C and F) Top activated and inhibited pathways in Srsf3-KO over Srsf3-WT liver (C) and breast (F) cancers by Gene Set Enrichment Analysis (GSEA) performed with the Hallmark gene sets. The Srsf3 KO-upregulated pathways in black color are common in both liver and breast cancer tissues

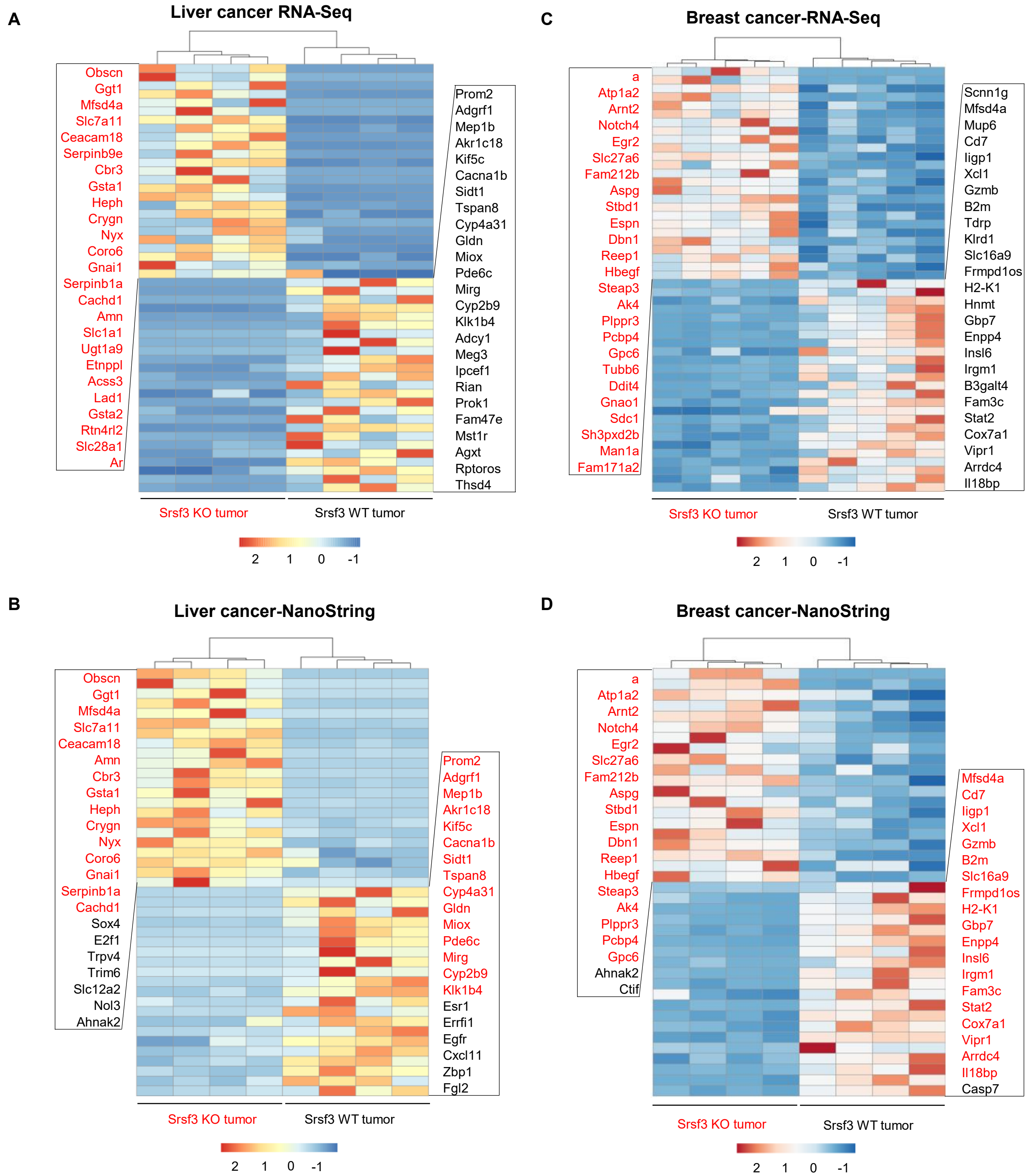

Fig. S7. Identification of the top Srsf3 KO-regulated gens by RNA-seq and validation by NanoString analysis in liver cancer and breast cancer. (A and C) Hierarchical clustering and heatmap of top 25 genes significantly upregulated and downregulated by Srsf3 KO in liver cancer (**A**) (Adjusted p ≤ 0.05, RPKM >=2) and in breast cancer (**C**) (Adjusted p ≤ 0.05, RPKM >=5) by RNA-seq analysis. (**B** and **D**) Selective validation by NanoString technology of the RNA-seq identified genes (in red) on the top listed up- or down-regulated expression and the genes (in black) with altered expression not on the top list, but with important functions by Srsf3 KO in liver cancer (**B**) and in breast cancer (**D**).

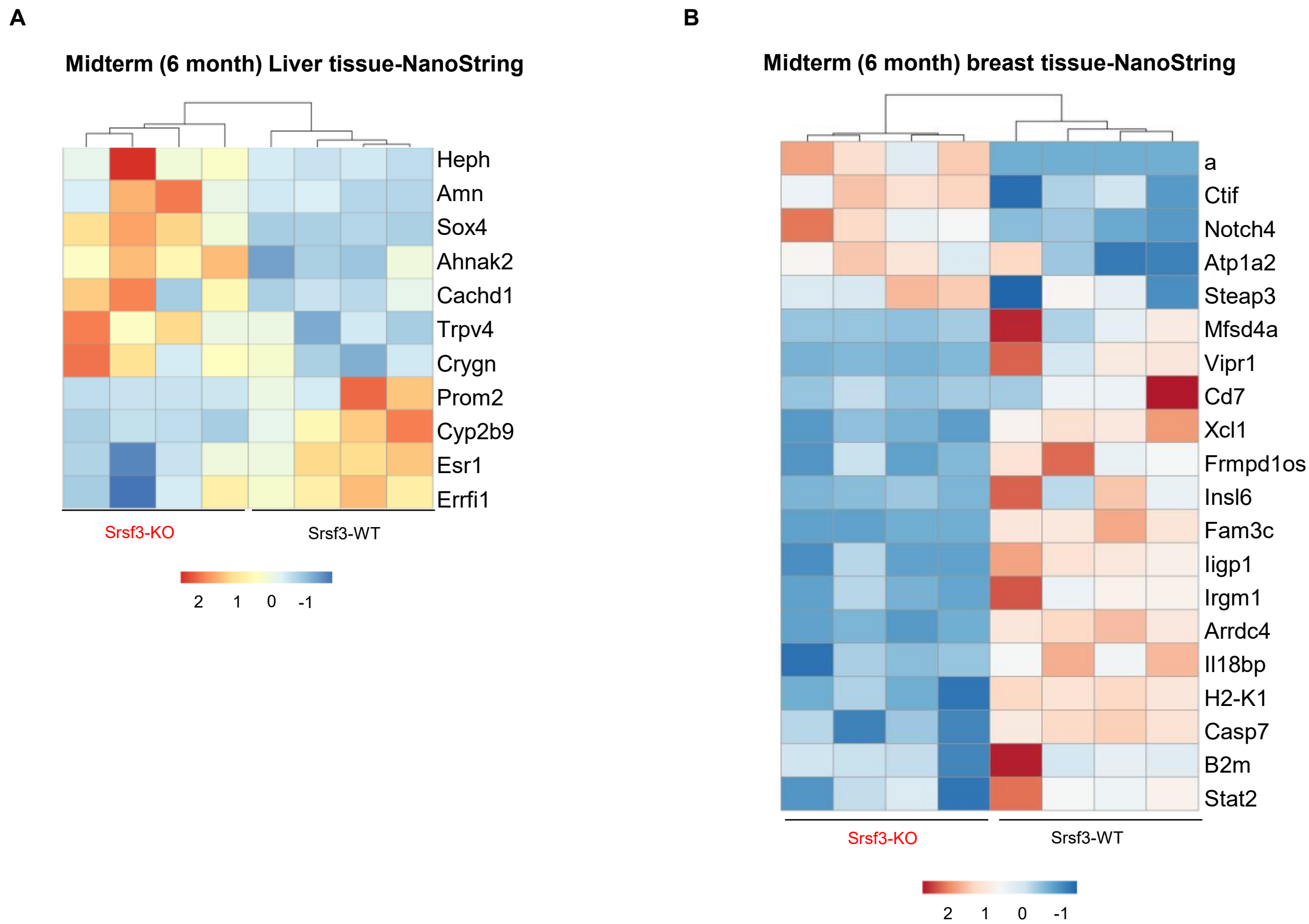

Fig. S8. Srsf3 KO in liver and breast tissues affects differential expression of a subset of genes. Total RNA isolated from the indicated mouse tissues with or without Srsf3 KO at six months of mouse age were used for NanoString RNA analysis. Mice donated liver tissues for this study had i.p. DEN injection at day 15 of mouse age. Heatmap shows differential expression of Srsf3 target genes identified in liver (A) and breast (B) tissues at this stage of animals.

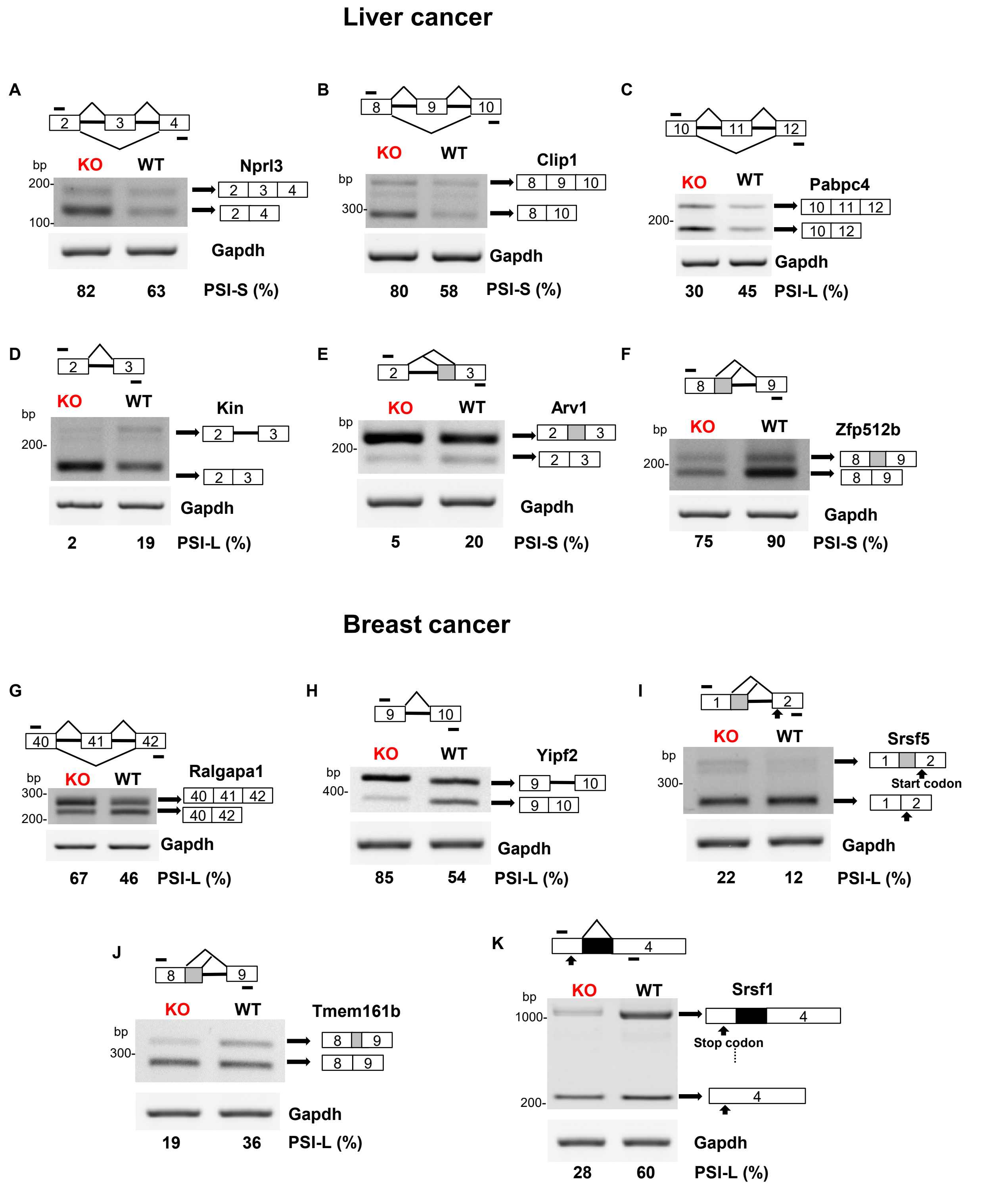

Fig. S9. Validation of significant RNA splicing events in differential expression of the Srsf3 KO-targeted genes in liver cancer (A-F) and breast cancer (G-K) tissues. Total RNA from the Srsf3-WT or srsf3-KO cancer tissues were analyzed by RT-PCR to validate the altered RNA splicing events identified by rMATS (p<=0.05, FDR<=0.05). (A-F) Srsf3 KO in liver cancer tissues led to skipping of Nprl3 exon 3 (A), Clip1 exon 9 (B), and Pabpc4 exon11 (C), but decrease Kin intron 2 retention (D), alternative 3′ ss usage in the Arv1exon 3 (E), and alternative 5′ ss usage in the Zfp512b exon 8 (F). (G-K) Srsf3 KO in breast cancer tissues led to increase Ralgapa1 exon 41 inclusion (G), intron 9 retention of Yipf2 (H), and alternative 5′ ss usage of Srsf5 exon 1 (I) but decrease alternative 5′ ss usage in Tmem161b exon 8 (J) and a cryptic intron inclusion within Srsf1 exon 4 (K). The primers used in RT-PCR are shown as bars above (forward primer) and below (reverse primer) each pre-mRNA diagram. Arrows indicate a translation start or stop codon. GAPDH served as a loading control. PSI, percent spliced-in of the alternative exon(s) or splice site (% inclusion = inclusion/sum of inclusion + exclusion).

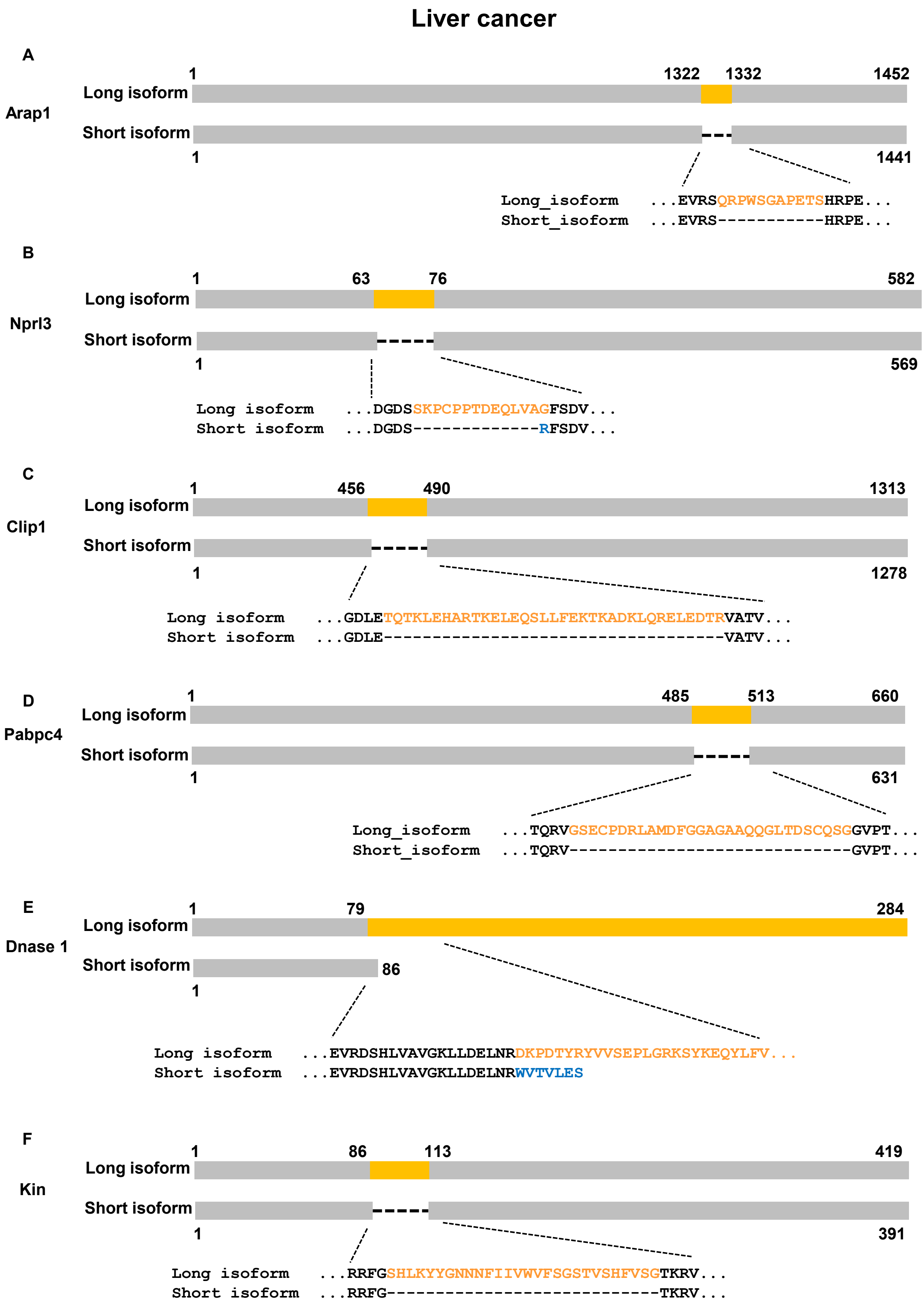

Liver cancer
A
Arap1
1
1322
1332
1452
Long isoform
Short isoform
1
1441
Long_isoform ...EVRSQRPWSGAPETSHRPE...
Short_isoform ...EVRS-----------HRPE...
B
Nprl3
1
63
76
582
Long isoform
Short isoform
1
569
Long isoform ...DGDSSKPCPPTDEQLVAGFSDV...
Short isoform ...DGDS-------------RFSDV...
C
Clip1
1
456
490
1313
Long isoform
Short isoform
1
1278
Long isoform ...GDLETQTKLEHARTKELEQSLLFEKTKADKLQRELEDTRVATV...
Short isoform ...GDLE-----------------------------------VATV...
D
Pabpc4
1
485
513
660
Long isoform
Short isoform
1
631
Long_isoform ...TQRVGSECPDRLAMDFGGAGAAQQGLTDSCQSGGVPT...
Short_isoform ...TQRV-----------------------------GVPT...
E
Dnase 1
1
79
284
Long isoform
Short isoform
86
1
Long isoform ...EVRDSHLVAVGKLLDELNRDKPDTYRYVVSEPLGRKSYKEQYLFV...
Short isoform ...EVRDSHLVAVGKLLDELNRWVTVLES
F
Kin
1
86
113
419
Long isoform
Short isoform
1
391
Long isoform ...RRFGSHLKYYGNNNFIIVWVFSGSTVSHFVSGTKRV...
Short isoform ...RRFG----------------------------TKRV...

Liver cancer

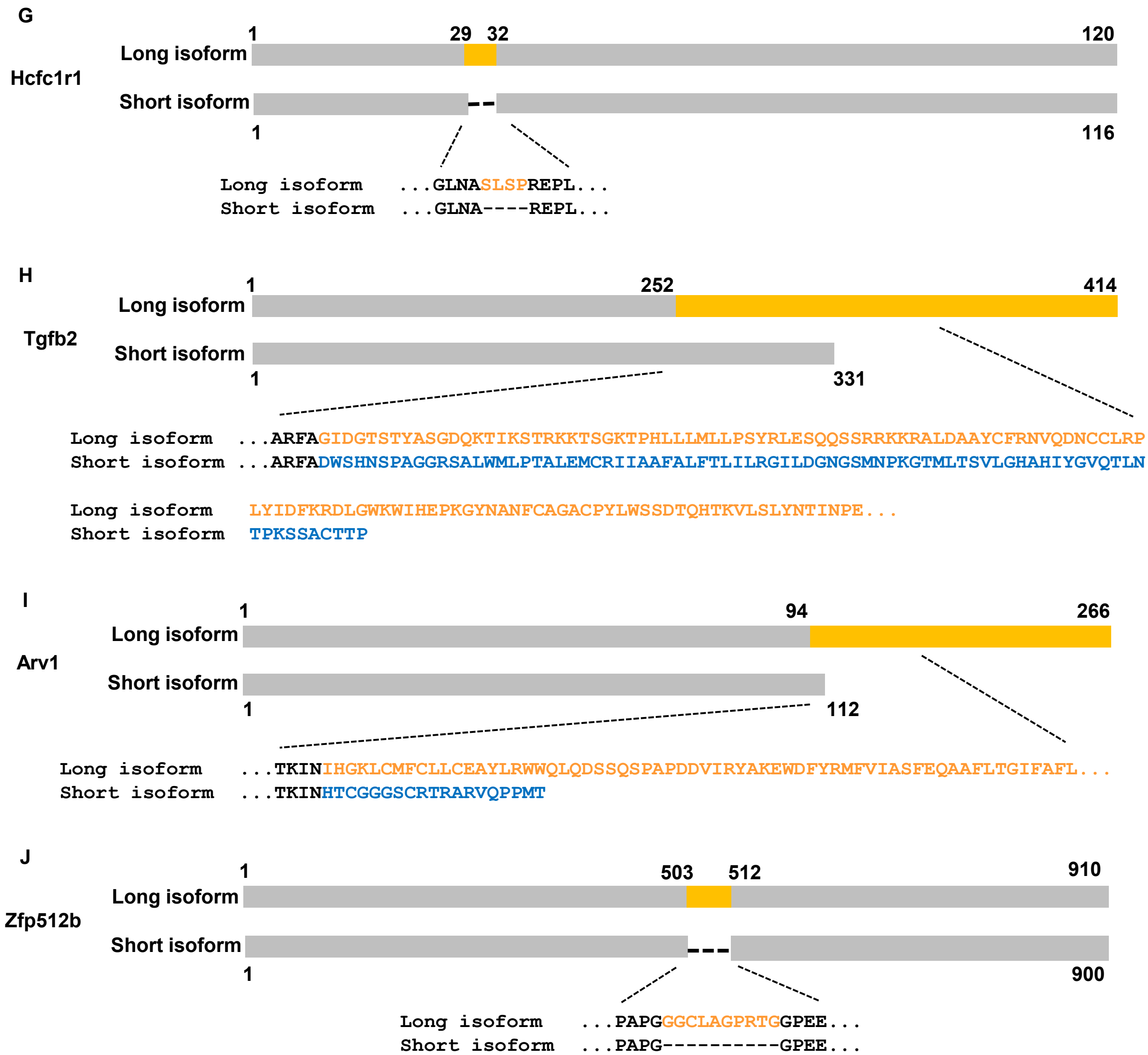

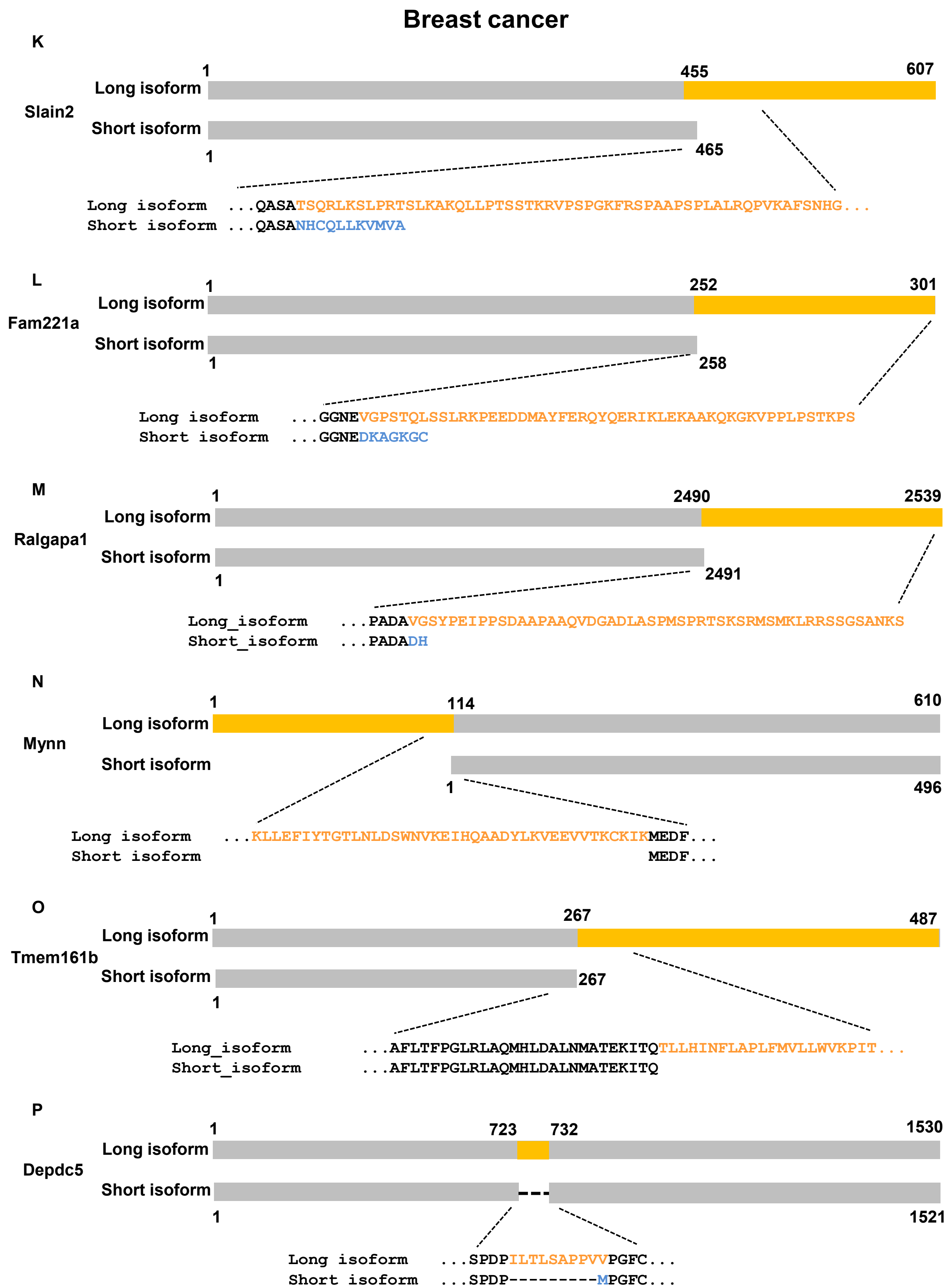

Fig. S10. Alignment of two protein isoforms encoded by validated Srsf3 KO-mediated RNA alternative RNA splicing of the targeted genes in liver cancer (A–J) and breast cancer (K–P). Two protein isoforms in each panel show the difference in amino acid sequences in the region derived from alternative RNA splicing, highlighted in yellow or blue, with black for their common amino acid residues.

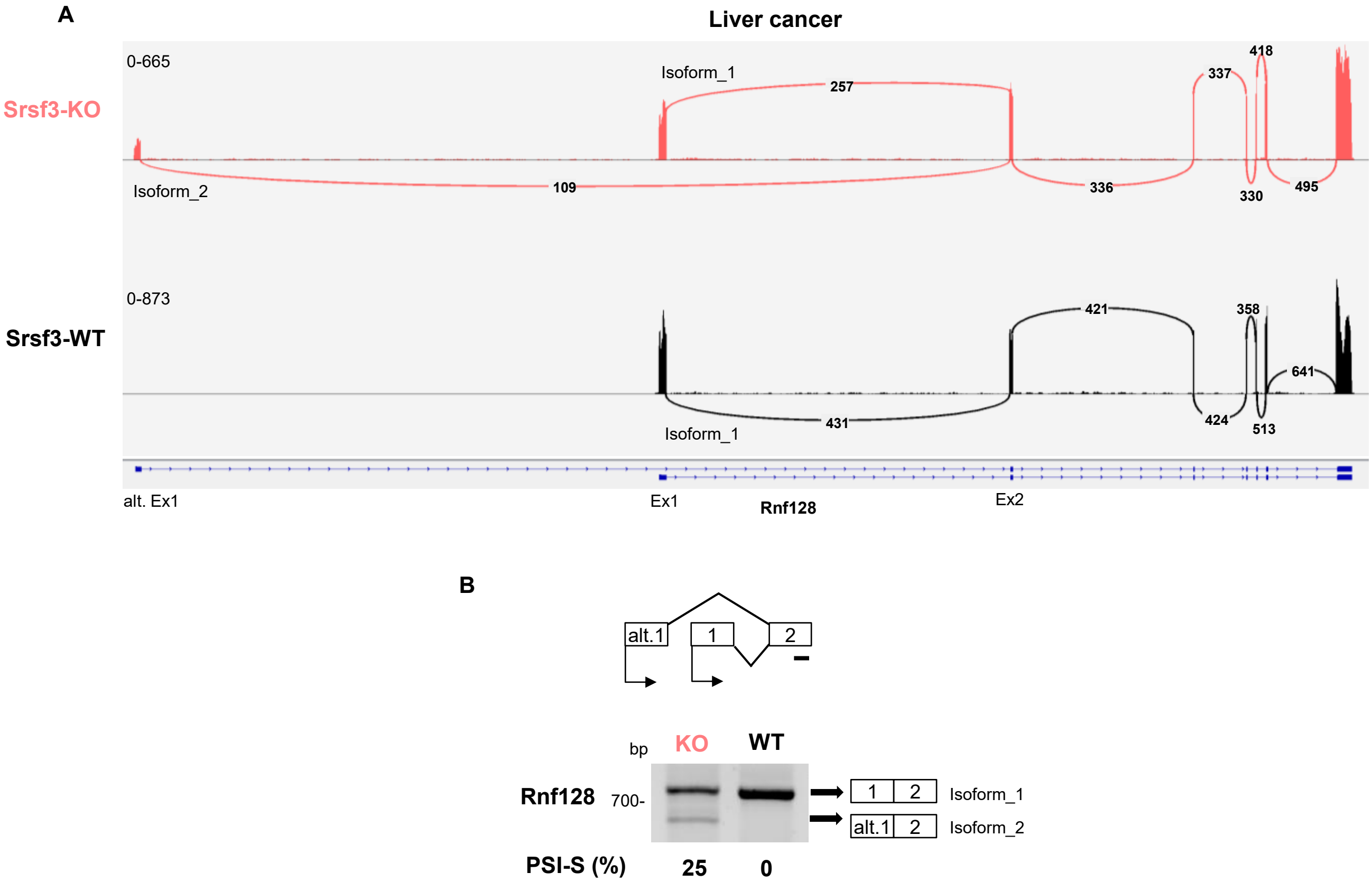

C

```
Isoform_1    MGPPPGIGVYCRGGCGAARLLAWCFLLALSPHAPGSRGAEAVWTAYLNVSWRVPHTGVNRTVWELSEEGVYGQD
Isoform_2    --------------------------MNRKTKHSFLQLLVFFTFLLQSTASFSMNAYVTVTYFNESSNYTKTER

Isoform_1    SPLEPVSGVLVPPDGPGALNACNPHTNFTVPTVWGSTVQVSWLALIQRGGGCTFADKIHLASERGASGAVIFNF
Isoform_2    CECGVYGLVSLVANAMGVVGIPKNGNYQACDYNTEFTNTAKPWIALIERGNCTFSEKIQTASRSNANAVVIYNS

Isoform_1    PGTRNEVIPMSHPGAGDIV.............. 428
Isoform_2    PETGNQTIQMANFGAGDIV.............. 402
```

Fig. S11. Srsf3 inhibits distal promoter usage of Rnf128 gene transcription in liver cancer. (A) Sashimi plots showing the alternative promoter usage and RNA splicing of Rnf128 gene expression in Srsf3 KO and WT liver cancer. (B) Validation of the Rnf128 promoter usage in Srsf3 KO and WT liver cancer tissues. A 5' RACE on total tissue RNA was applied to mapping the Rnf128 transcription start site (TSS) from mouse liver cancer tissues with a mouse Rnf128-specific primer oMA421 from exon 2. Two major RACE products from Srsf3 KO liver tumor and the isoform 1 product from the Srsf3 WT liver cancer were gel purified separately and sequenced by the Sanger sequencing. PSI, percent spliced-in of the alternative exon(s) or splice site (% inclusion = inclusion/sum of inclusion + exclusion). (C) The alignment of two different isoforms of Rnf128 protein.

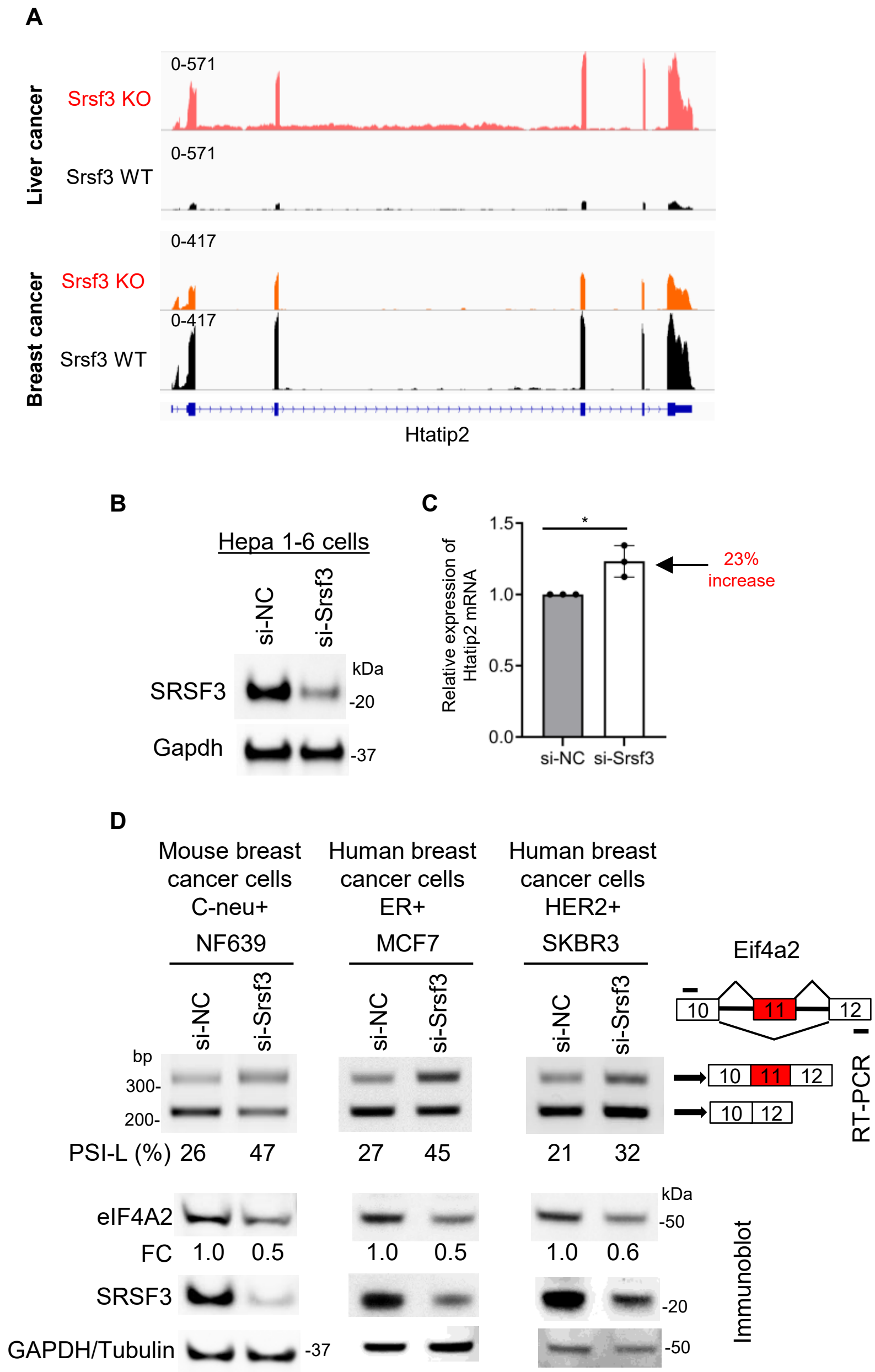

Fig. S12. (A) Srsf3 KO increases Htatip2 expression in liver cancer but decreases Htatip2 expression in breast cancer. RNA-seq reads coverage of Htatip2 was visualized by IGV. (B) Srsf3 KD in Hepa 1-6 cells, a murine hepatoma cell line, increases expression of Htatip2. Hepa1-6 cells were transfected with 40 nM of Srsf3-specific siRNA (si-Srsf3) or non-targeting siRNA control (si-NC) and harvested at 48 h upon transfection for analysis. Srsf3 KD efficiency was confirmed by Western blot using a Srsf3 7B4 monoclonal antibody with Gapdh serving as a sample loading control. (C) Srsf3 KD in Hepa 1-6 cells increases expression of Htatip2 examined by TaqMan RT-qPCR in three independent experiments. *, $p < 0.05$ by Student's t-test. (D) Srsf3/SRSF3 KD promotes exon 11 (a poison exon) inclusion in Eif4a2 RNA splicing and reduction of eIF4A2 protein in mouse and human breast cancer cells. Total cell RNA was extracted from the indicated cell lines transfected with 40 nM of the indicated siRNAs and harvested at 48 h after transfection and used for RT-PCR assays with a forward primer from exon 10 and backward primer from exon 12 (top panel). % spliced index of long form (PSI-L) was calculated as described in Fig. S9. Total cell lysates from the corresponding cell lines prepared at the 48 h after siRNA transfection were immunoblotted for the protein expression of eIF4A2, SRSF3, GAPDH (NF639), and Tubulin (MCF7 and SKBR3) with the corresponding antibodies (low panel). Fold change (FC) was calculated as described in Fig. 4F.

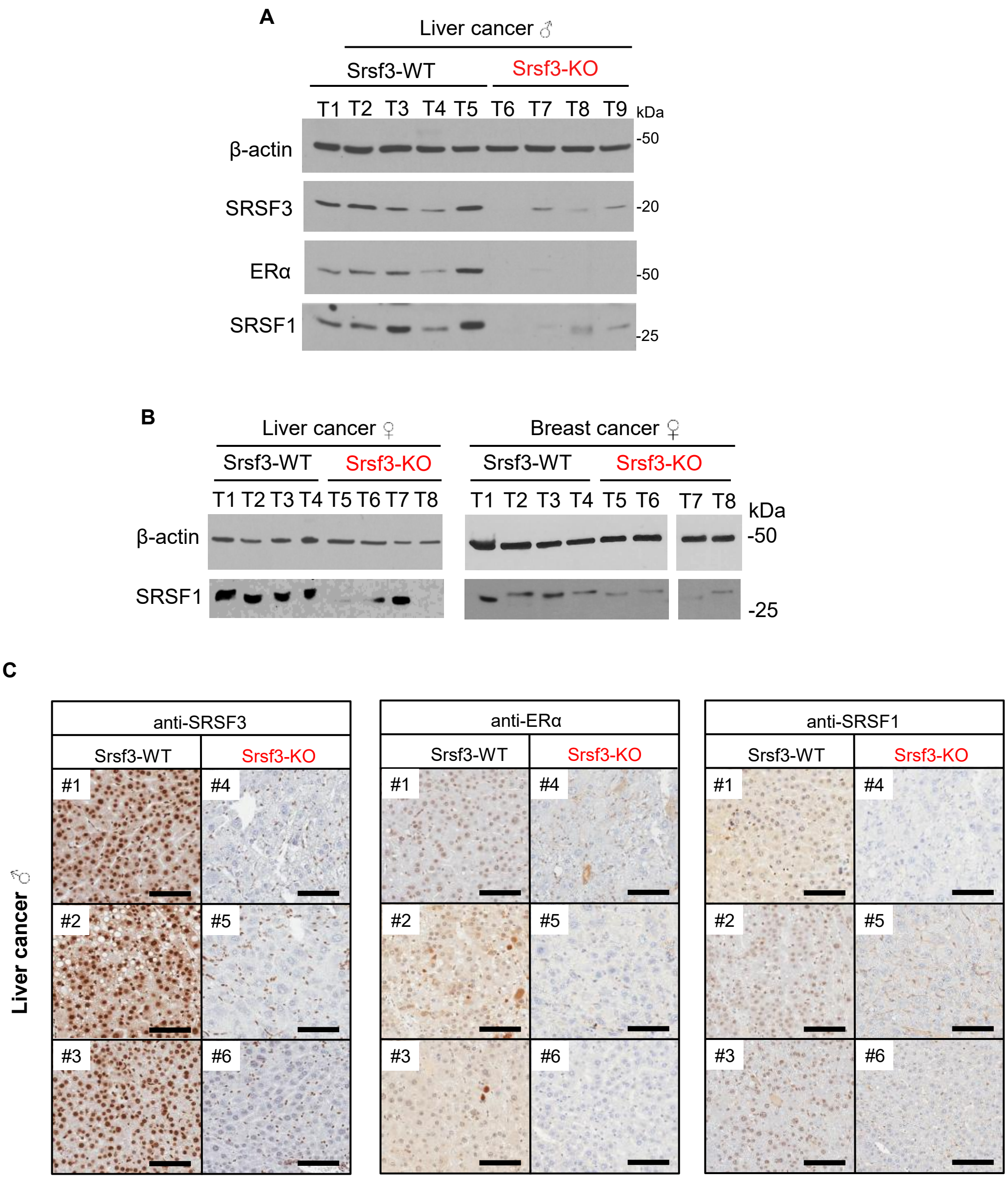

Fig. S13. Srsf3 KO decreases expression of liver ERα and SRSF1 proteins in male mice (♂) . (A) Decreased expression of ERα and SRSF1 proteins in Srsf3-KO liver cancer in male mice (♂) . Five mice with DEN-induced Srsf3 WT liver cancer tissues were compared with four mice with DEN-induced Srsf3 KO liver cancer tissues by Western blot using a corresponding antibody to SRSF3, ERα, or SRSF1, along with β-actin serving as a protein loading control. (B) Srsf3 KO reduces the expression of SRSF1 in female (♀) liver cancer and breast cancer tissues. Four cancer tissues in each group were immunoblotted by an anti-SRSF1 antibody. β-actin served as a protein loading control. (C) Immunohistology staining of SRSF3, ERα and SRSF1 protein in male (♂) Srsf WT and Srsf3 KO liver cancer tissues using a corresponding antibody as indicated. Three pairs of representative samples in each group were shown. Scale bar, 100 μm.

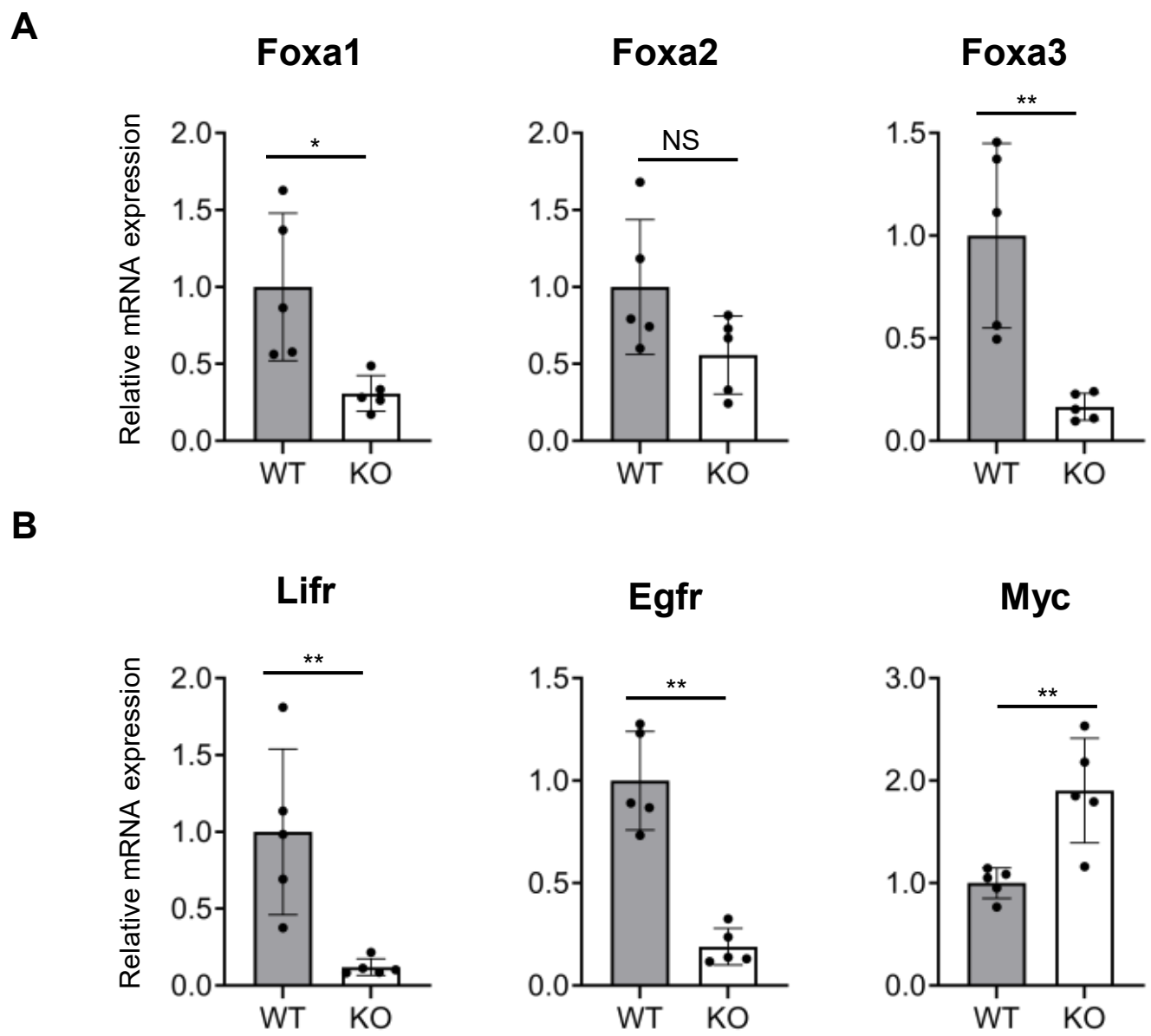

Fig. S14. RNA expression of Foxa1, Foxa 2, Foxa 3 (A) and Lifr, Egfr, Myc (B) in Srsf3-WT and Srsf3 KO male liver cancer tissues by quantitative RT-qPCR. Five samples were in each group. NS, not significant; *, $p < 0.05$; **, $p < 0.01$ by Student's t-test.

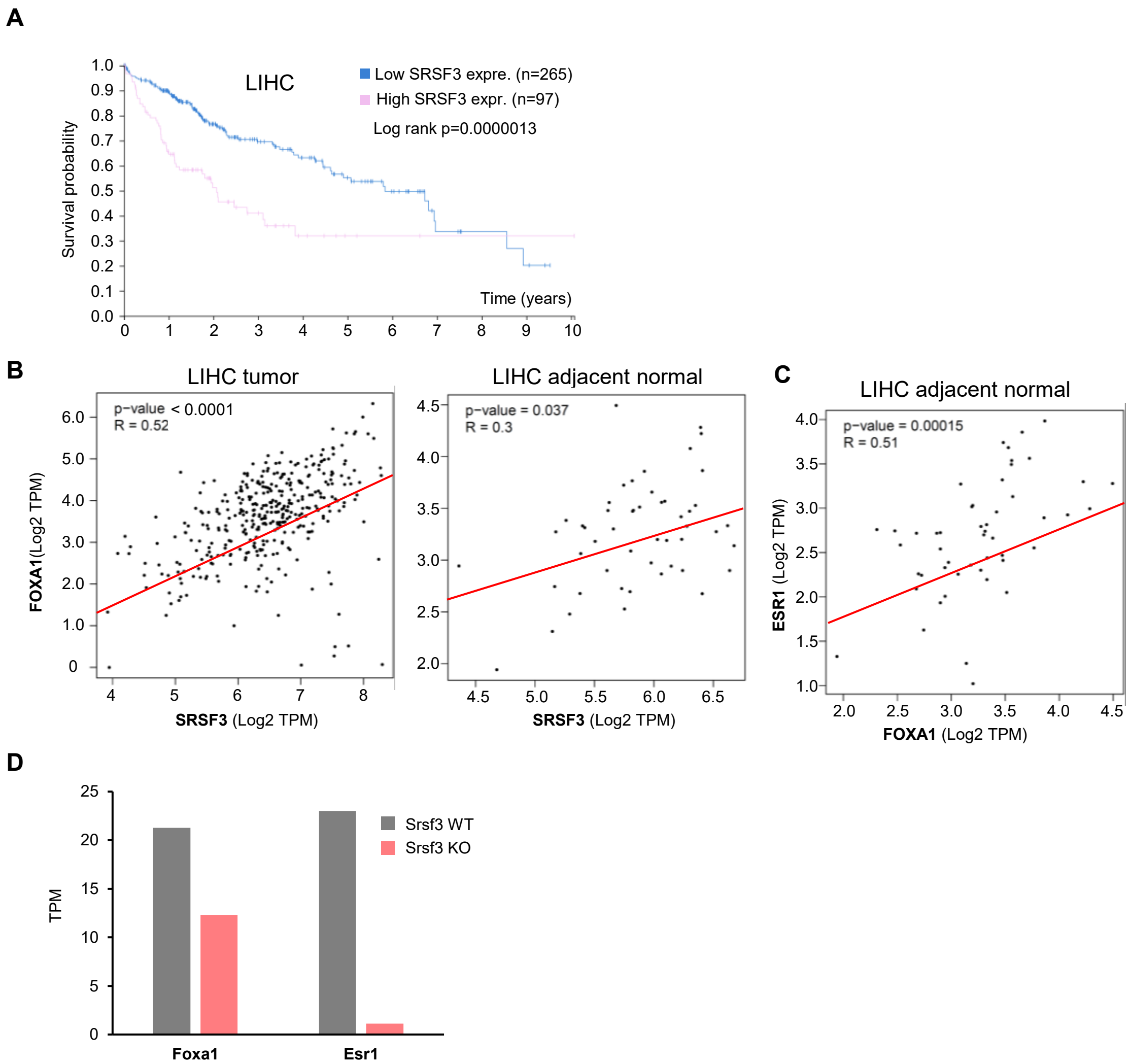

Fig. S15. SRSF3 and LIHC survival and expression correlation of SRSF3, FOXA1 and ESR1. (A) Kaplan–Meier overall survival analysis of patients with liver hepatocellular carcinoma (LIHC). The survival curve was generated using the Human Protein Atlas (https://www.proteinatlas.org) based on TCGA LIHC RNA-seq data. Patients were stratified into high (n = 97) and low (n = 265) SRSF3 expression groups. Statistical significance was determined by the log-rank test ($P = 1.3 \times 10^{-5}$). (B) Co-expression correlation of SRSF3 and FOXA1 mRNAs in LIHC tumor (left) and LIHC adjacent normal tissues (right) using TCGA RNA-seq data analyzed through GEPIA2 (http://gepia2.cancer-pku.cn). A positive correlation was observed in both LIHC tumors (left; R = 0.52, P < 0.0001) and LIHC adjacent normal tissues (right; R = 0.30, P = 0.037). (C) Co-expression correlation of FOXA1 and ESR1 mRNAs in LIHC adjacent normal tissues using TCGA RNA-seq data was analyzed through GEPIA2 as in (B), showing a significant positive correlation (R = 0.51, P = 0.00015). (D) RNA-seq analysis of four female mouse liver cancer for Foxa1 and Esr1 co-expression by comparing two Srsf3-KO liver cancer with two Srsf3-WT cancer tissues. Bar graphs show positive co-expression levels of Foxa1 and Esr1 quantified as TPM (transcripts per Million mapped reads) in relation to SRSF3. See the RT-qPCR validation of Foxa1 in Fig. 6B and Esr1 in Fig. 5B.

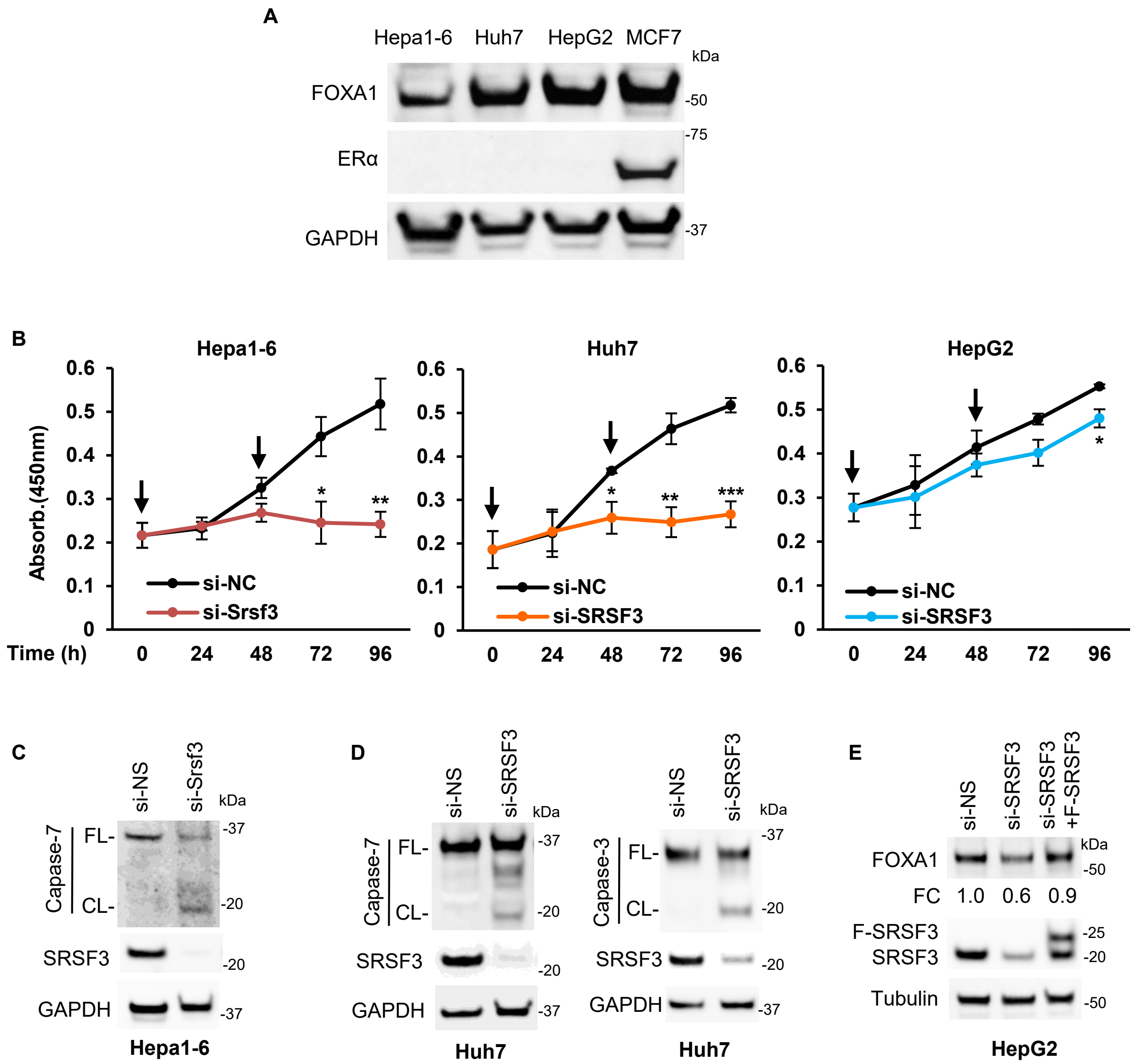

Fig. S16. FOXA1 and ERα detection, KD of SRSF3 expression, and rescuing FOXA1 expression by exogenous SRSF3. (A) Endogenous FOXA1 and ERα protein expression in liver and breast cancer cell lines. Protein levels of FOXA1 and ERα were analyzed by Western blot in mouse (Hepa1-6) and human (Huh7 and HepG2) liver cancer cells and human ERα-positive breast cancer cells (MCF7) 24 h after plating. GAPDH served as a loading control. (B) KD of SRSF3 expression in hepatocellular cancer-derived cells (mouse Hepa1-6 and human Huh7) inhibits cell growth but does only minimally for human hepatoblastoma-derived HepG2 cells. Cell proliferation was assessed by CCK-8 assay at the indicated time points following twice siRNA transfections (0 and 48 h; arrows) of 40 nM si-NC (a non-targeting siRNA control) or si-Srsf3 (Srsf3-specific siRNA) each time. Cell viability was measured by absorbance at 450 nm. Statistics was determined by two-way ANOVA from three independent experiments. *$p < 0.05$, **$p < 0.01$, ***$p < 0.001$. (C, D) Srsf3 KD induces cell apoptosis in Hepa1-6 and Huh7 cells. Western blot analysis showed activation and increased cleavage of caspase 7 in both cell lines (C, D) and of caspase 3 (D) in Huh7 cells when Srsf3 expression was knocked down. Cell transfection with indicated siRNAs (40 nM) was performed as in (B) and cell lysates were collected at 96 h and analyzed by Western blot with GAPDH serving as a loading control (C, D). (E) Rescue of FOXA1 expression by exogenous SRSF3 in HepG2 cells. HepG2 cells were transfected with 40 nM si-NC or si-SRSF3 at the time of plating. The cells at 24 h later were transfected with a FLAG-SRSF3 (F-SRSF3) expression plasmid (pMA50) and harvested 24 h after plasmid transfection for Western blot analysis. Tubulin served as a loading control. Relative FOXA1 expression was quantified after normalization to tubulin.

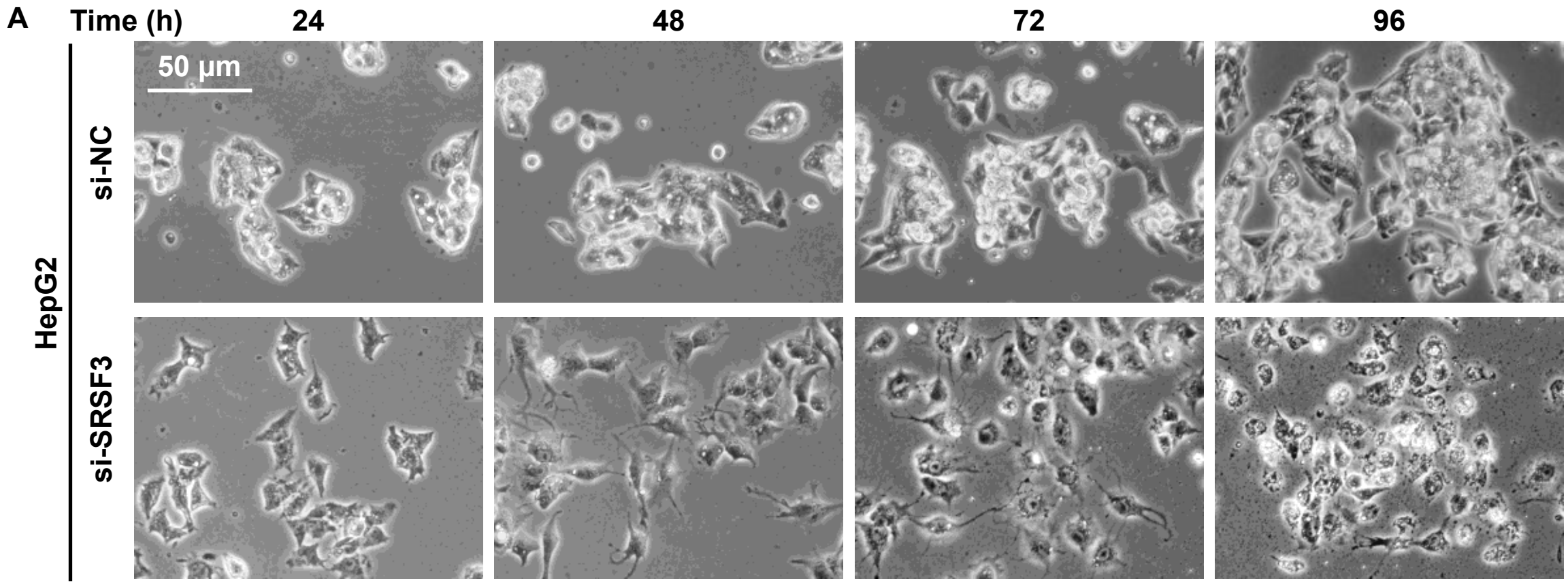

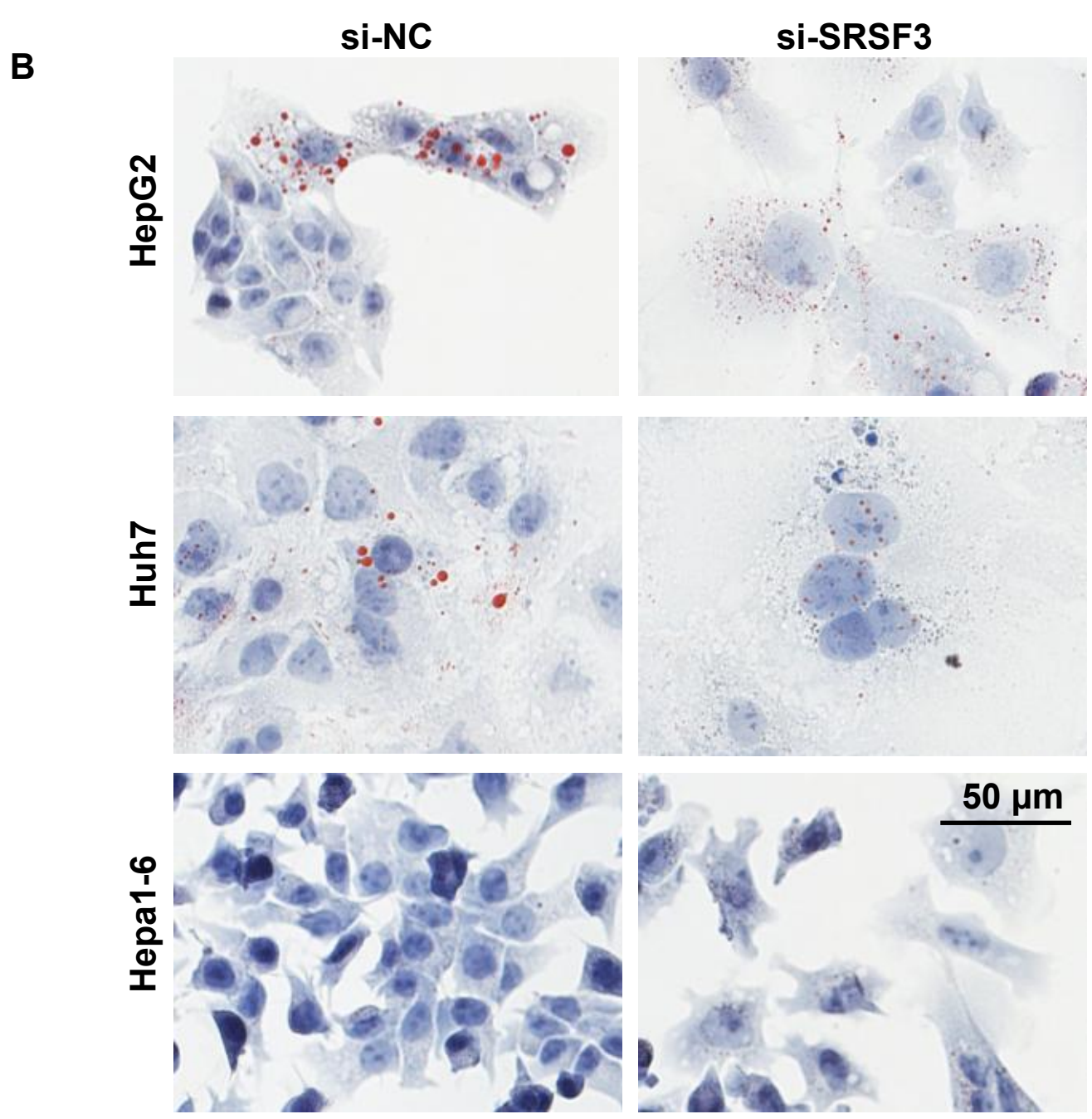

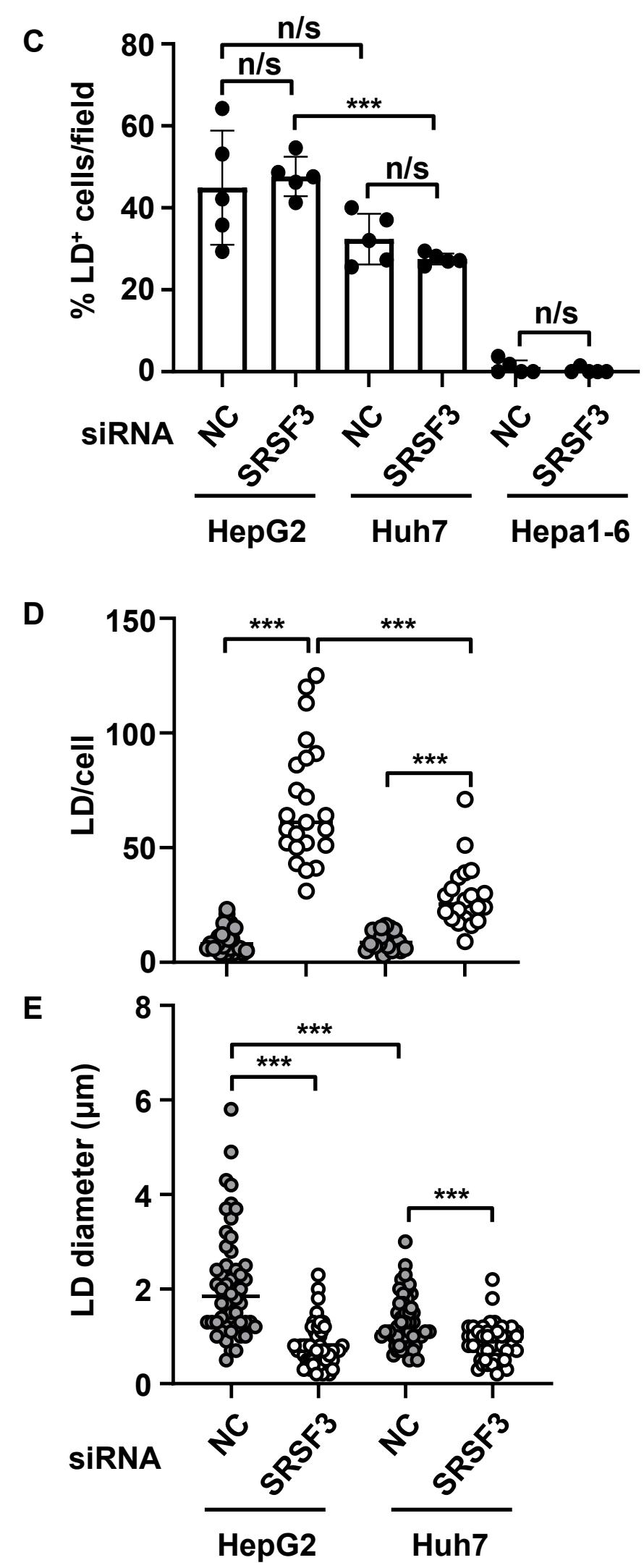

F

Feature	Treatment	HepG2	Huh7	Hepa1-6
LD+ cells (%) (n=5)	si-NC	43.8	33.0	1.1
	si-SRSF3	47.1	27.4	0.4
LD/cell (n=20)	si-NC	10.5	9.5	N/D
	si-SRSF3	70.8	28.8	N/D
LD diameter (n=50)	si-NC	2.1	1.3	N/D
	si-SRSF3	0.8	0.9	N/D

Fig. S17. Hepatoblastoma-derived human HepG2 cells are distinguishable from hepatocellular carcinoma-derived human Huh7 and mouse Hepa1-6 cells by cell morphology and lipid metabolism in response to SRSF3 KD. (A) Morphological changes in HepG2 cells following SRSF3 KD. Representative brightfield images are HepG2 cells transfected with 40 nM control siRNA (si-NC) or SRSF3-specific siRNA (si-SRSF3) and cultured for the indicated times (h). (B-F) Lipid droplet staining and measurements. HepG2, Huh7, and Hepa1-6 cells were plated on coverslips and treated with si-NC or si-SRSF3. Lipid droplets (LD) at 48 h after siRNA treatment were stained with Oil Red O (Abcam, ab150678) (B). Cells were counterstained with hematoxylin (Modified Mayer's solution). LD⁺ cells per microscopy field were calculated and averaged as mean ± standard deviation from five independent fields per sample (C). Number of LD per cell was counted and averaged from 20 individual cells in each sample group (D). LD size was measured in diameter of 50 individual LDs in the cells from each sample group using scanned images generated by a HALO Link software (E). Mean values from each measurement are summarized in a table panel (F). N/D, not determined. Statistical significance was assessed using Student's *t*-test for pairwise comparisons. ***, $p < 0.001$; n/s, not significant.

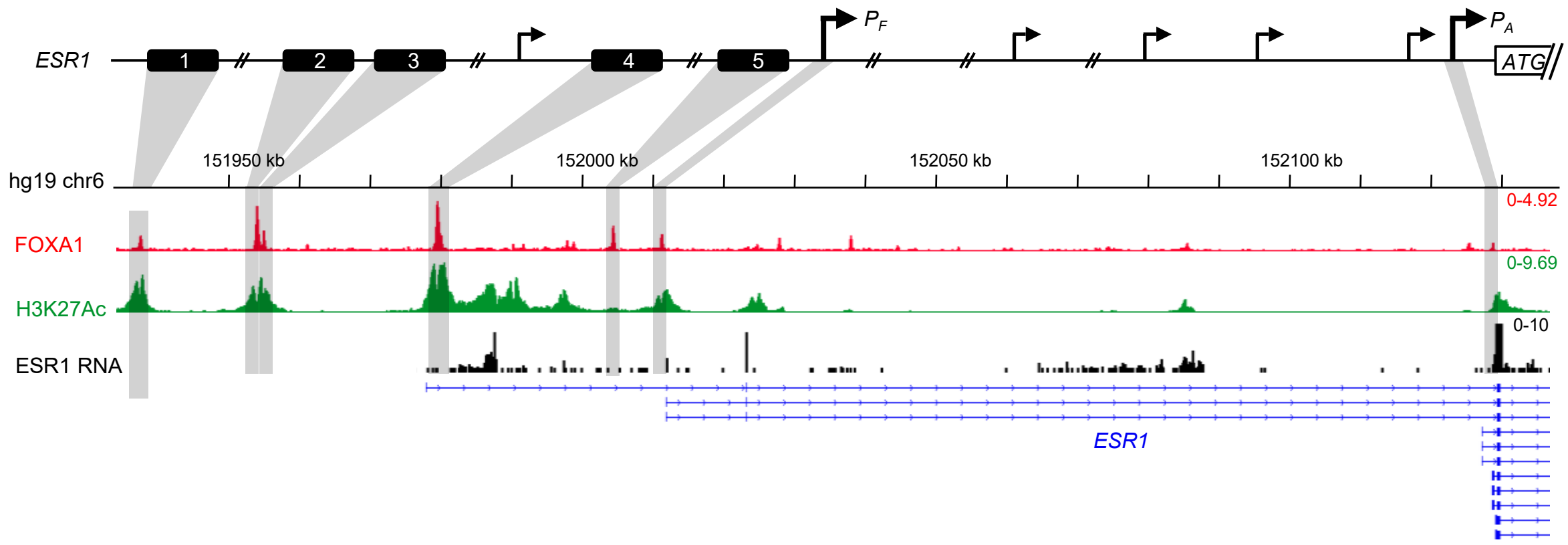

Fig. S18. Transcriptional regulation of human *ESR1* by FOXA1. Schematic representation of the transcription regulatory elements of human *ESR1* promoter (top), including the enhancers (numbered black boxes) and alternative promoter transcriptional start sites (arrows with P_A and P_F being two major ones) (10). The lower draws and graphs show ChIP-seq-determined FOXA1 occupancy (red) and associated H3K27 acetylation (H3K27Ac, green) at the chr6 *ESR1* promoter region in estrogen-treated MCF7 cells. The ESR1 RNA expression shown in black bars was determined by RNA-seq. All datasets used for the analyses were obtained from GSE152367 (11).

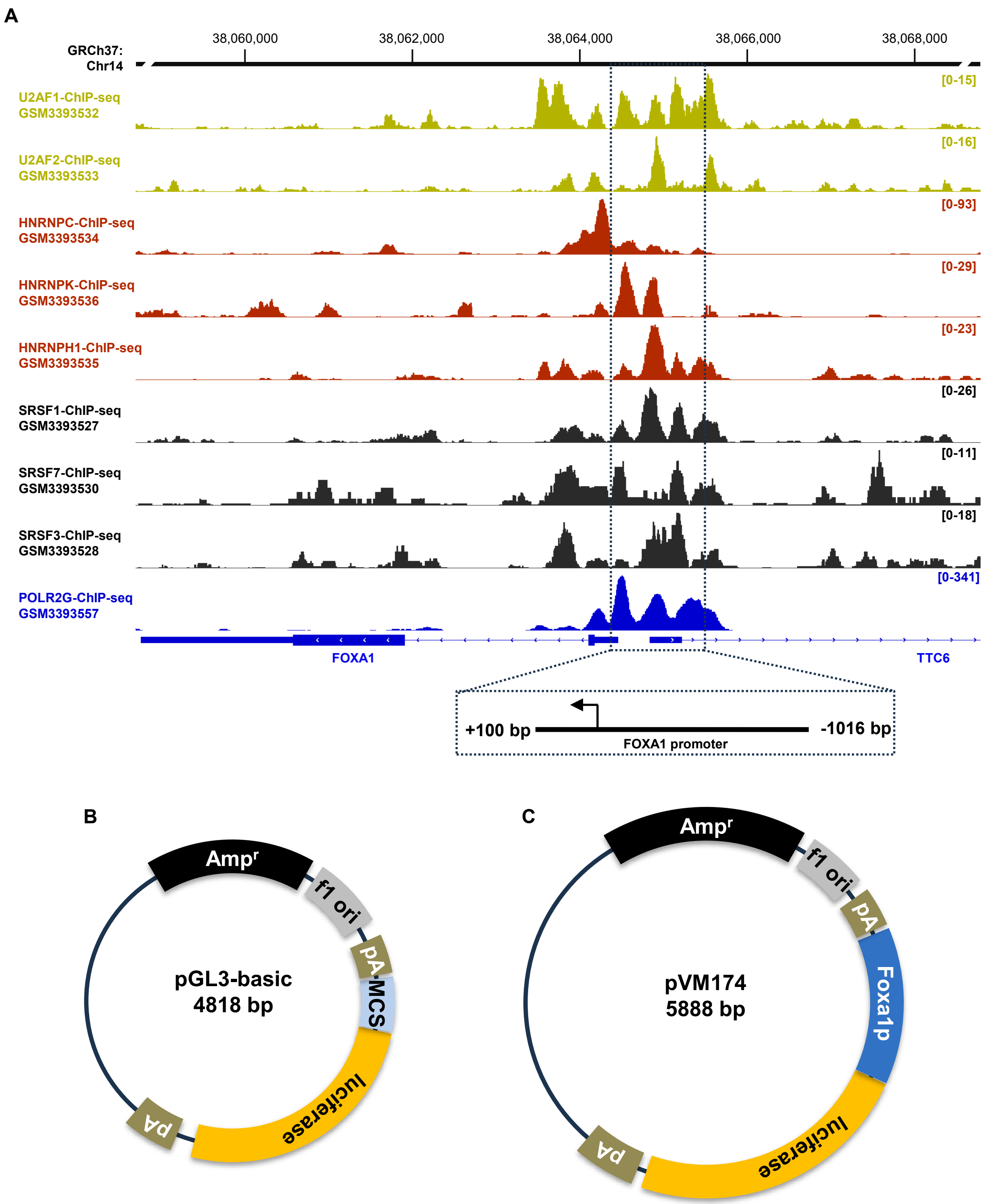

Fig. S19. SRSF3 association with the FOXA1 promoter region. (A) Binding profiles of several RNA-binding proteins to the FOXA1 promoter region including splicing factors U2AF1, U2AF2, hnRNP C, hnRNP K, hnRNP H1, SRSF1, SRSF7, SRSF3 and RNA polymerase II subunit G (POLR2G) are originated from the ChIP-seq data available at NCBI GEO Series (7). **(B** and **C**). Luciferase reporters used in the study. Promoter-less firefly luciferase plasmid pGL3-basic purchased from Promega was used as a negative control (**B**). The mouse Foxa1 promoter (Foxa1p, -1016/+100) region amplified from mouse hepatocellular cancer cell line Hepa1-6 cells was inserted at *Kpn I* and *Hind III* sites upstream of the firefly luciferase ORF in pGL3-basic MCS to generate plasmid pVM174 (**C**). Amp^r - ampicillin resistance gene; f1 ori - f1 origin of replication; MCS – multiple cloning sites; pA - polyadenylation site.
